# Crafting Stable Antibiotic Nanoparticles via Complex Coacervation of Colistin with Block Copolymers

**DOI:** 10.1021/acs.biomac.4c00337

**Published:** 2024-06-17

**Authors:** Thomas D. Vogelaar, Anne E. Agger, Janne E. Reseland, Dirk Linke, Håvard Jenssen, Reidar Lund

**Affiliations:** † Department of Chemistry, University of Oslo, P.O. Box 1033, Blindern, NO-0315 Oslo, Norway; ‡ Department of Biomaterials, Institute of Clinical Dentistry, University of Oslo, P.O. Box 1109, Blindern, NO-0317 Oslo, Norway; § Department of Biosciences, University of Oslo, P.O. Box 1066, Blindern, NO-0316 Oslo, Norway; ∥ Department of Science and Environment, Roskilde University, 4000 Roskilde, Denmark; ⊥ Hylleraas Centre for Quantum Molecular Sciences, University of Oslo, NO-0315 Oslo, Norway

## Abstract

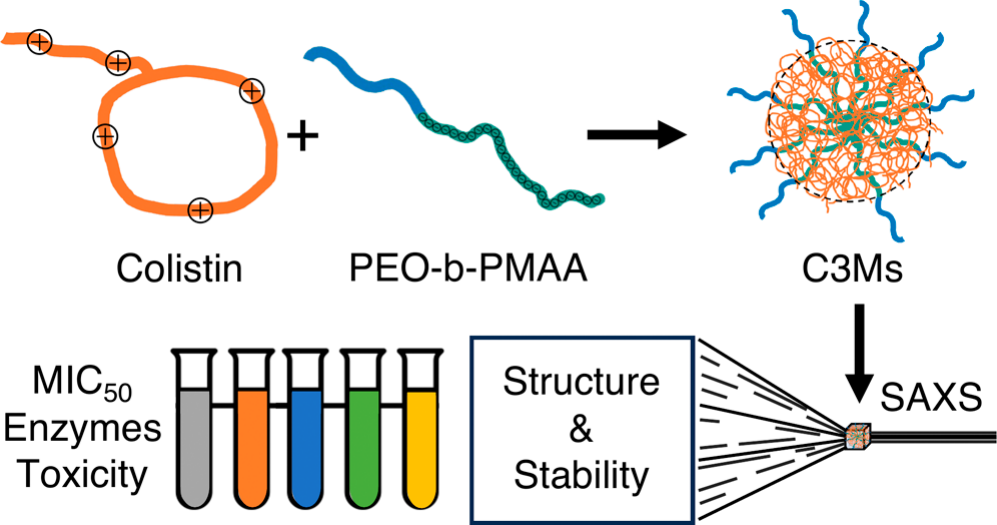

To combat the ever-growing increase of multidrug-resistant (MDR) bacteria, action must be taken in the development of antibiotic formulations. Colistin, an effective antibiotic, was found to be nephrotoxic and neurotoxic, consequently leading to a ban on its use in the 1980s. A decade later, colistin use was revived and nowadays used as a last-resort treatment against Gram-negative bacterial infections, although highly regulated. If cytotoxicity issues can be resolved, colistin could be an effective option to combat MDR bacteria. Herein, we investigate the complexation of colistin with poly(ethylene oxide)-*b*-poly(methacrylic acid) (PEO-*b*-PMAA) block copolymers to form complex coacervate core micelles (C3Ms) to ultimately improve colistin use in therapeutics while maintaining its effectiveness. We show that well-defined and stable micelles can be formed in which the cationic colistin and anionic PMAA form the core while PEO forms a protecting shell. The resulting C3Ms are in a kinetically arrested and stable state, yet they can be made reproducibly using an appropriate experimental protocol. By characterization through dynamic light scattering (DLS) and small-angle X-ray scattering (SAXS), we found that the best C3M formulation, based on long-term stability and complexation efficiency, is at charge-matching conditions. This nanoparticle formulation was compared to noncomplexed colistin on its antimicrobial properties, enzymatic degradation, serum protein binding, and cytotoxicity. The studies indicate that the antimicrobial properties and cytotoxicity of the colistin-C3Ms were maintained while protein binding was limited, and enzymatic degradation decreased after complexation. Since colistin-C3Ms were found to have an equal effectivity but with increased cargo protection, such nanoparticles are promising components for the antibiotic formulation toolbox.

## 1 Introduction

To address the ongoing rise of multidrug-resistant bacteria, proactive steps are essential for the advancement of antibiotic formulations.^1,2^ Over the last few years, antimicrobial peptides (AMPs) have received more attention as the new generation of bactericidal compounds.^2−4^ AMPs are naturally occurring peptides, part of the innate immune system in a broad range of organisms.^2^ Contrary to conventional antibiotics, the mode of action of AMPs is related to their amphiphilic nature, which results in an affinity toward the cell membrane, creating various disruptive effects.^2,5−7^ Their cationic properties create selective affinity toward net negatively charged bacterial membranes over more zwitterionic mammalian cells.^2−4^

One of the oldest classes of AMPs, polymyxins, has been used for decades in clinical practices.^8,9^ Polymyxin E, also known as colistin, derived from the Gram-positive *Paenibacillus* genus, was discovered in 1947. After extensive use in the 50s and 60s, it was found that colistin is nephrotoxic and even mildly neurotoxic. Consequently, its use was banned in the 1980s.^8^ However, due to a shortage of bactericidal compounds against Gram-negative bacteria, colistin has seen a partial resurgence and is nowadays used as a last-resort treatment against Gram-negative bacterial infections, administered either ectopically or intravenously.^8,10^ Colistin is composed of a fatty acid chain to which a cyclic decapeptide is coupled that contains five l-α-γ-diaminobutyric acid residues. These side groups are the cause of a positive net charge of +5 in physiological conditions. As colistin is cationic, it strongly interacts with anionic lipopolysaccharides (LPS) by electrostatic attraction and cation displacement on the outer membrane of Gram-negative bacteria, after which it efficiently kills the bacteria by penetration of the inner membrane.^11,12^ Even though colistin has the highest affinity for LPS, colistin is also able to increase the tubular epithelial cell membrane permeability of mammalian cells, resulting in cell swelling and lysis.^12^ This in turn leads to cytotoxic effects in humans.^4,12^ It is a general belief that if the cytotoxicity can be reduced, the use of colistin as an antibiotic will be significantly expanded as it is cheap and effective.^13,14^

Several strategies for colistin toxicity reduction have already been assessed over the years that mainly focus on facilitating or encapsulating colistin, aiming to decrease the undesired interactions of colistin causing toxicity.^13,15−21^ E.g., conjugation with hyaluronan-chitosan derivatives has been investigated,^15^ but also drug delivery systems like colistin loading into liposomes,^13^ chelating micelles,^16^ aerosolizable particles,^17^ nanostructured lipid carriers (NLCs),^18,19^ solid lipid nanoparticles (SLNs),^18^ poly(lactic-*co*-glycolic acid) (PLGA) nanoparticles,^20^ and complexation into coacervates.^21^ In the development of drug delivery methods for colistin, several factors need to be controlled, like solubility, retainability, bioavailability, and functionality. Currently, the main concerns with colistin drug delivery are its stability, chemical alteration, and poor water solubility, leading to low bioavailability.^18,22,23^ To resolve these challenges, going deeper into complex coacervation could be a viable strategy as it does not require any chemical modification of the drug and allows for effective encapsulation of water-soluble drugs.^24,25^ However, this requires sufficient colloidal stability of the formed complexes. Complexation of colistin could improve the retainability and stability by protecting the drug from degradation and enhancing its therapeutic efficacy in various pharmaceutical formulations. As an additional benefit, colistin effectivity can be improved as well, as most AMPs are known for their low *in vivo* stability due to protease degradation.^26^

In complex coacervation, microphase separation is induced due to electrostatic attraction and counterion release between oppositely charged polyelectrolytes, thus creating a phase with water-soluble complex coacervates.^27−29^ These charged polyelectrolytes are most often grafted, random, or block copolymers but can also be homopolymers, proteins, nutraceuticals, peptides, DNA, (m)RNA, or drugs.^24,27,30^ In complex coacervation drug delivery, a charged drug or charge-conjugated drug is complexed with another polyelectrolyte, which often is an ionic polymer or lipid.^27^ One of the more recent applications of this methodology is in vaccine technology, in which mRNA is complexed with cationic lipids into lipid nanoparticles.^31−33^

To ensure sufficient colloidal stability and prevent macroscopic phase separation, in many cases, a neutral hydrophilic block is conjugated to one of the charged species.^21,27,33,34^ When these charged polyelectrolytes are mixed in favorable charge conditions, the charged polyelectrolytes form a core, and the (neutral) hydrophilic block forms a shell. The shell protects the highly charged core from interaction with other highly charged cores, significantly increasing its stability.^27,28,34^ These structures are termed complex coacervate core micelles (C3Ms).^24,27,28,34^ Typically, to form C3Ms, block copolymers are used consisting of a charged block and a neutral hydrophilic block.^27^ Most often, the neutral block that provides colloidal stability through steric repulsion is poly(ethylene oxide) (PEO),^26,28,35,36^ but sometimes other polymer blocks are used, such as poly(vinyl alcohol) (PVA)^37^ or poly(acrylamide) (PAAm),^38^ to name a few. Previously, other proteins, drugs, and peptides, e.g., (helical) polypeptides,^39,40^ doxorubicin,^41^ lysozyme,^42,43^ myoglobin,^43^ bovine serum albumin (BSA),^44^ and many others have been successfully complexed into C3Ms.^23,24,27,45,46^ In contrast, complex coacervation involving AMPs is limited to a few studies.^21,25,47−51^ The AMPs polymyxin B,^48^ colistin,^21^ temporin-L,^47^ KSL-W,^51^ P6,^49^ and MSI-78^50^ have successfully been complexed into coacervates, with only the latter two into C3Ms, which are generally recognized for their enhanced stability compared to typical coacervates.^27^ Răileanu et al. complexed P6 with PEO-*b*-poly(acrylic acid) (PAA),^49^ while Wang et al. prepared C3Ms from the complexation of MSI-78 with methoxyPEO-*b*-poly(α-glutamic acid) (PGlu).^50^ As shown by these examples, in combination with the limited research on the detailed structural investigation of the AMP complexes, the complexation of AMPs is still widely unexplored, even though this structural analysis is potentially highly relevant in the development of new antibiotic formulations.

Herein, we describe the formulation of well-defined spherical and highly stable C3Ms based on the coassembly of colistin with poly(ethylene oxide)-*b*-poly(methacrylic acid) block copolymers (PEO-*b*-PMAA) without the use of any additional stabilizing agent, covalently bound species, or any other harmful or harsh chemicals. Our primary motivation was to create a stable and tunable drug delivery system in which colistin is complexed with another component to form C3Ms. Three different PEO-*b*-PMAA polymers were investigated for complexation with colistin at several charge fractions. We employed P1: PEO_45_-*b*-PMAA_41_, P2: PEO_45_-*b*-PMAA_81_, and P3: PEO_114_-*b*-PMAA_81_ (numbers indicate the degree of polymerization) to achieve nanoparticle complexes with optimal stability and a reproducible structure. The next step comprised the characterization of the properties of the formed C3Ms. We characterized the complex coacervate using scattering techniques: small-angle X-ray scattering (SAXS) and dynamic light scattering (DLS) to investigate nanostructure and stability, next to bacterial testing, enzymatic protection, human serum albumin (HSA) binding, and cell toxicity testing. We show that the colistin-C3Ms are highly stable and reproducible, with the highest colistin complexation effectivity found around charge matching. Similar antibiotic activity and cytotoxicity were found between colistin and colistin-C3Ms, while the complexation of colistin was found to improve the protection against enzymatic degradation, while limited protein binding effects were observed. With the increased protection of colistin, while maintaining its activity, colistin-C3Ms could potentially be a new option in the toolbox of antibiotic formulations for intravenous clinical purposes.

## 2 Experimental Section

### 2.1 Complex Coacervate Preparation

PEO-*b*-PMAA at different block lengths (Polymersource) and colistin sulfate (Sigma Aldrich) were dissolved in 0.05 M TRIS buffer (Sigma Aldrich) (pH = 7.4). Depending on the charge fraction (*f*_+_), the samples were diluted separately to two times the desired final concentration before mixing the polymer into colistin solution 1:1 volume-wise. It was made sure that the solution was mixed properly. Final concentrations of either 5.0, 2.5, 1.3, or 1.0 mg/mL were used for analysis and diluted even further when necessary.

### 2.2 ζ-Potential

ζ-Potential measurements were carried out using the Malvern Zetasizer Nano ZS with Malvern DTS1070 cuvettes. ζ-Potentials were measured between 12 and 20 times for every sample in triplicate at a total concentration of 1.0 mg/mL at 20 °C.

### 2.3 Dynamic Light Scattering (DLS)

Dynamic light scattering (DLS) experiments were performed using a DLS/SLS instrument from LS Instruments, equipped with a Cobolt high-performance DPSS laser 100 mW (660 nm). The samples were filtered either through 0.22 or 0.45 μm poly(vinylidene difluoride) (PVDF) filters (Millipore) directly into precleaned 2 mm NMR tubes. Complex coacervates were measured at 2.5 or 1.3 mg/mL at 20 °C, including regular checks to avoid multiple scattering and check for concentration-dependent effects. For stability measurements, DLS measurements were taken with one-day intervals in the first week, three-day intervals in the second and third week, and then weekly until three months if no aggregation was observed.

### 2.4 Small-Angle X-ray Scattering (SAXS)

The small-angle X-ray scattering profiles were measured at 20 °C using the BioSAXS beamline BM29^52^ at the European Synchrotron Radiation Facility (ESRF) in Grenoble, France. The automated sample changer loaded 50 μL for every sample into a quartz glass capillary of a diameter of 1 mm. Ten scattering frames of 1.0 s each were detected on the Pilatus 3 × 2 M detector, using an energy of 12.5 keV and a sample–detector distance of 2.81 m, measuring a *Q*-range of 0.007–0.55 Å^–1^. The background sample (0.05 M TRIS buffer, pH = 7.4) was measured between each sample measurement, and the capillary was cleaned between every measurement. Water was used as a primary standard to scale the data to absolute intensities. Every frame was checked for radiation damage, followed by averaging, buffer subtraction, and binning (from 1000 points to 280), resulting in the final scattering curves that are presented in this paper. Additional SAXS experiments were performed at our in-house Bruker NanoStar instrument (RECX, University of Oslo, Norway). The instrument uses Cu Kα radiation (λ = 1.54 Å) and yields scattering data in the *Q* range 0.01–0.3 Å^–1^. According to instrument standard procedures, the scattering intensity was corrected for detector sensitivity, electronic noise, and empty cell scattering, and calibrated to absolute units using water scattering, yielding the macroscopic differential scattering cross-section d∑/dΩ. Then the scattering contribution from the solvent was subtracted.

### 2.5 SAXS Data Modeling

Fixed partitioning, based on calculated charge matching in the core, was assumed. This significantly reduced the number of fit parameters in the model. With the model, it was possible to get a quantitative analysis of the complex coacervate structures. The fuzzy-surface complex coacervate model with broad interfaces and polydispersity consists of three separate contributions: complex coacervate scattering, free (noncomplexed) polymer and colistin scattering, and an additional structure factor to describe the physical interactions between polyelectrolytes in the core (more elaborate explanation of the model can be found in the Supporting Information). The total scattering function on an absolute scale can be described by eq 1:
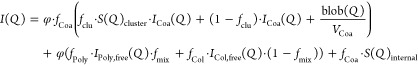
1In which φ is the volume fraction, *f*_Coa_ is the fraction of coacervates, *V*_Coa_ is the volume of one complex coacervate, *f*_clu_ is the fraction that is forming clusters, *f*_Poly_ is the free fraction of polymer, *f*_Col_ is the free fraction of colistin, and *f*_mix_ is the molar fraction of polymer in the aqueous phase surrounding the complex coacervates. These fractions are based on the aforementioned assumption that there is stoichiometric charge matching in the core of the complex coacervates.

For the first contribution, the complex coacervates, we used the form factor of a “fuzzy sphere”, i.e., spheres with graded interfaces described by the radius *R* and width of the interface, σ. Based on the trial fit analysis, we ignored the core–shell nature of the C3Ms since the contrast between the core formed with colistin and PMAA and the PEO shell was too small to allow for a clear separation. Therefore, we described the model of C3Ms with a sphere with a fuzzy interface instead. The form factor for fuzzy spheres (*P*(*Q*) = *A*_core_(*Q*)^2^) can be described, as follows, and was incorporated in the scattering function for complex coacervates (*I*_Coa_(*Q*))^53,54^ (eqs 2–6).
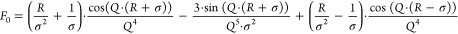
2
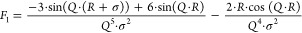
3
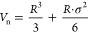
4
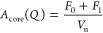
5
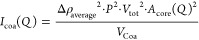
6where *I*_coa_(*Q*) is the scattering contribution from the complex coacervates, Δρ_average_ is the average scattering length density of the complex coacervates, *P* is the aggregation number (number of molecules per micelle), and *V*_tot_ is the total volume of the blobs. In addition to the form factor, the blob scattering^35,55−59^ of the polyelectrolytes in the core of the complex coacervates was considered (eq 7):
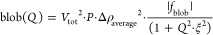
7where *f*_blob_ is the fraction of blobs and ξ is the blob correlation length. For the second contribution to the model, for the free component, noncomplexed, scattering of polymer and colistin, the Debye form factor for polymers and polyelectrolytes is used^60^ (eq 8).
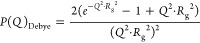
8In which *R*_g_ is the radius of gyration of the polyelectrolyte. The third contribution is the internal structure factor, indicated in Figure 2A. This feature is related to the charge correlations between the blobs of anionic PMAA and cationic colistin^59^ and is summarized in eq 9, which was built upon already existing models in the literature.^35,55−60^
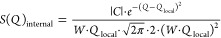
9where *C* is the fractal scattering, *W* is the relative width at high *Q*, and *Q*_local_ is the internal scattering location. In the presence of globular proteins, we approximated the scattering contribution using a prolate/oblate ellipsoid form factor (eqs 10–13). The ellipsoid of revolution has two minor core radii of *R* and major axis ε*R*, respectively. The form factor can be written as:
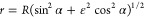
10
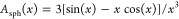
11
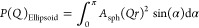
12The total contribution can then be written as:
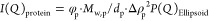
13where φ_*p*_ is the volume fraction of the globular protein, *M*_w,p_ is the molecular weight of the protein, *d*_p_ is the solution density, and Δρ_p_ is scattering length density incorporated by values based on the fitting of the data of pure globular protein samples at three different concentrations. In the case of the addition of another compound next to the complex coacervates, the fitted random chain (Debye equation in eq 8) or ellipsoidal scattering (form factor in eq 12) of these compounds was added to the scattering from the complex coacervate, and then fitted, while the other parameters were kept the same.

For the fit analysis, least-squares fit routines were applied keeping the following parameters free: the total aggregation number (*P*), the radius of the core including its density distribution, and polydispersity index (*R*_in_, σ_in_, PDI), the free fraction of colistin (*f*_Col_), and if necessary, in the presence of cluster formation, the number of complexes in a cluster (*N*_*clu*_) and *f*_dist_ the distance between complexes. These parameters mostly describe the low and intermediate *Q* range. After obtaining a rough fit of low and intermediate *Q*, to also fit the data well at high *Q*, the scattering from the blobs themselves was fitted. Moreover, the blob scattering parameters blob fraction (*f*_blob_) and blob correlation length (ξ) were fitted. Lastly, to describe the blob charge correlations (internal structure), the relative width high *Q* (*W*), the internal scattering location (*Q*_local_), and fractal scattering (*C*) were fitted. Finally, all parameters were fitted to the data simultaneously, resulting in the fits presented in this paper.

### 2.6 Assessing the Effect of Ionic Strength and pH on Colistin-C3Ms

The effect of ionic strength was analyzed in two different setups, the addition of NaCl (Sigma Aldrich) before mixing and after mixing, also known as salt annealing. P1-colistin-C3Ms at *f*_+_ = 0.50 were prepared in the same way but with the addition of 0.15 M NaCl in the buffer and mixing using a stopped-flow device, mixing at 6.7 mL/s. Salt annealing was performed by the addition of salt after preparation of the C3Ms at five different concentrations (0.05, 0.10, 0.15, 0.30, and 0.50 M NaCl). For the effect of pH changes, C3Ms were prepared in the same way but in five other pH buffers, citrate buffer at pH = 5.0, maleate buffer at pH = 6.0 and pH = 7.0, and TRIS at pH = 8.0 and pH = 8.7 (all buffers from Sigma Aldrich). All these samples were analyzed using SAXS and modeled accordingly.

### 2.7 Determination of Critical Micelle Concentration (CMC)

Using an optical tensiometer setup the CMC of C3Ms at *f*_+_ = 0.50 was determined. Drops were captured by a CCD camera with a tensiometer setup from Ramé-Hart, inc. The surface tension from the drops was analyzed by using “Drop image”. 100 Frames were captured for each measurement, after which the surface tension was modeled using the Young–Laplace equation and averaged. To determine the CMC, two regions were distinguished between 0.0 and 5.0 mg/mL total concentration. The region of fast surface tension decreases, and the part in which low surface tension decreases is observed. The transition point corresponds to the CMC.

### 2.8 Antimicrobial Properties of C3Ms and Colistin

To compare the antimicrobial properties of C3Ms to colistin two different methods were tested: disk diffusion assay (DDA) and agar broth dilution testing for MIC_50_ determination.

In the DDA, the susceptibility of *Escherichia coli* (DSM613), *Serratia indica* (NCIMB 8869), *Pseudomonas fluorescens* (DSM20030), *A. vinelandii* (DSM2290), and *Bacillus subtilis* (DSM10) was tested. Sterile filter disks (VWR) were impregnated (using an automatic pipette) with 3.0 μg of colistin from either colistin sulfate or colistin-C3Ms (at *f*_+_ = 0.50) in a concentration above the CMC (at 1.0 mg/mL) and dried under sterile air. Bacterial plates were prepared by freshly pouring bacterial medium until it set. Second, a layer of LB medium, that was premixed with 10 μL of bacteria from overnight cultures, was added. Then, the disks were added, and the plates were incubated for 24 h at 37 °C. The sizes of the inhibition zones were measured and averaged. Experiments were performed in triplicate. With the agar broth dilution testing, it was possible to determine the MIC_50_ values. LB liquid media in test tubes containing equal amounts of *E. coli* culture (10 μL of bacteria from overnight cultures to 5 mL of media) was mixed with either colistin or colistin-C3Ms with equal colistin for a concentration between 0.0 and 50 μg/mL. The test tubes were incubated for 24 h at 37 °C and their turbidity was measured with a McFarland turbidimeter. The agar broth dilution experiments were done in duplicate. To determine the MIC_50_ values, curves were averaged and then fitted with a sigmoid function.

### 2.9 Enzymatic Susceptibility

The enzymatic breakdown susceptibility was analyzed by adding proteinase K (3.4.21.64; Sigma Aldrich), subtilisin (3.4.21.62; Sigma Aldrich), and trypsin (3.4.21.4; Sigma Aldrich) to either colistin (at 3.4 mg/mL) or C3Ms at *f*_+_ = 0.50 (total concentration 5.0 mg/mL) at their suppliers’ recommended concentration/ratio for optimized breakdown (molar ratios of enzyme:colistin ≈ 1:130 for proteinase K, ≈ 1:70 for subtilisin and ≈ 1:180 for trypsin). We assessed the difference in susceptibility between noncomplexed colistin and C3Ms. Colistin solution was incubated with enzymes for 24 h at 37 °C, after which polymer solution (kept 24 h at 37 °C) was added while in the other case, premade C3Ms were incubated with enzymes for 24 h at 37 °C. These samples were analyzed using SAXS and modeled, including enzyme scattering (using the Debye form factor since the scattering was too small to use a prolate/oblate ellipsoid form factor, eq 8) to monitor the molecular weight of the complex coacervates after exposure to enzyme degradation.

### 2.10 HSA Binding Susceptibility

To assess the binding affinity of colistin-C3Ms at *f*_+_ = 0.50 with HSA, HSA was added to C3Ms (5.0 mg/mL) at two different molar ratios (1:10 and 1:20) at room temperature (20 °C), and then analyzed using SAXS and modeled including HSA (Sigma Aldrich) (using the prolate/oblate ellipsoid form factor, eq 12) scattering to monitor changes in structure and size of the formed complex coacervates in the presence of HSA.

### 2.11 Toxicity Assay

To be able to use the colistin-C3Ms, colistin, and PEO-*b*-PMAA needed for the toxicity assessment, the components were freeze-dried at a total concentration of 1 mg/mL by freezing solutions in liquid nitrogen, after which they were exposed to a vacuum for 24 h. Human embryonic kidney (HEK 293 cells) (ATCC, CRL-1573) were cultured in Dulbecco’s modified Eagle medium (DMEM), low glucose (Sigma Aldrich) supplemented with 100 U/mL penicillin and 100 μg/mL streptomycin (15140-122; Gibco, Waltham), 1% GlutaMAX (35050; Gibco), and 20% fetal bovine serum (F9665; Sigma Aldrich). Mesenchymal stem cells (MSC, PT-2501; Lonza) were cultured in Mesenchymal Stem Cell Basel Medium (PT-3238; Lonza)) supplemented with MSCGM SingleQuots (PT-4105; Lonza). Human gingival keratinocytes (HGK) (PCS-200-014; ATCC) were cultured in Dermal Cell Basal Medium (PCS-200-030; ATCC) supplemented with Keratinocytes Growth Kit (PCS-200-040; ATCC). Human umbilical cord endothelial cells (HUVEC, 200p-05n; Cell Applications) were cultured in Endothelial Cell Growth Medium (211-500; Cell Applications). The cell types were seeded in 12-well culture-treated plates at a density of 4 × 10^4^ cells/cm^2^. Upon confluency (set at time point 0), the cells were exposed to cell-specific growth medium containing formulations with a concentration of 1.0 mg/mL total concentration (0.7 mg/mL colistin) of *f*_+_ = 0.50 C3Ms or equivalent concentrations of colistin, and polymer as a control. The medium was changed for both control cells and stimulated cells every two days, 24 h before the harvest of the medium to ensure the same window of accumulated cell-secreted factors in the media. Cytotoxicity assays on lactate dehydrogenase (LDH) activity and caspase-3 level were performed at 24, 48, and 72 h and executed in triplicate. LDH activity in the cell culture media was evaluated using a cytotoxicity detection kit (11644793001; Roche). In short, 50 μL of the collected supernatant was mixed with 50 μL of the reaction mixture and incubated in a dark room at room temperature for 30 min. The absorbance was measured at 490 and 600 nm using the BioTek ELx800 Absorbance Microplate Reader. The caspase-3 activity in the cell culture media was evaluated using an enzyme-linked immunosorbent assay (K4221-100; BioVision). A 100 μL sample was added to each well, and the plate was incubated at 37 °C for 1.5 h. After incubation, 100 μL of biotin-detection antibody was added and incubated at 37 °C for 1 h. The plate was washed three times, after which 100 μL of horseradish peroxidase (HRP)-streptavidin conjugate was added, and the samples were incubated at 37 °C for 30 min. The wells plate was washed five times and 90 μL of 3,3′,5,5′-tetramethylbenzidine (TMB) substrate was mixed in, after which the plate was incubated at 37 °C for 30 min. After incubation, 50 μL stop solution was added. The absorbance was measured at 450 nm using the BioTek ELx800 Absorbance Microplate Reader. After harvesting, light microscopy at a magnification of 10 times was utilized to visualize morphology changes of different cell types upon treatments.

## 3 Results and Discussion

### 3.1 Charge Dependency and Stability

To analyze the charge dependency of the formation and stability of the colistin complex coacervates, we first investigated the range in which colistin complex coacervates could be formed. To quantify the charge ratios mixing charge fractions were calculated (eq 14), in which a value of 0.50 indicates the charge matching of colistin and PEO-*b*-PMAA.^27^
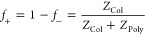
14

**Figure 1:**
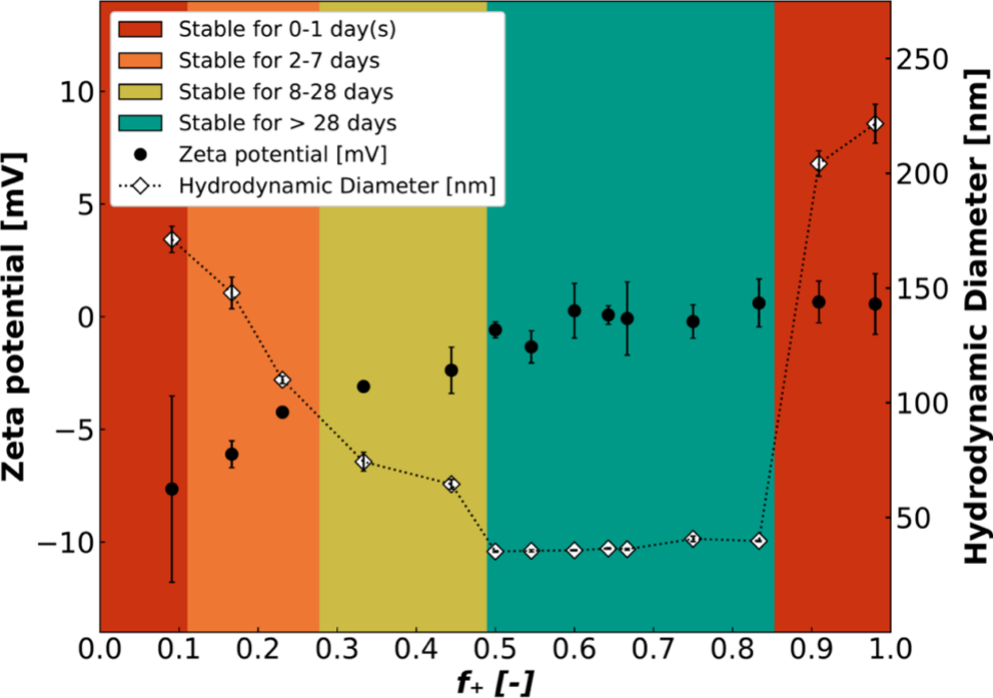
ζ-Potential measurements (filled black circles) and DLS size measurements right after mixing (open white diamonds), including error bars, combined with the stability of complex coacervates mixing PEO_45_-*b*-PMAA_41_ (P1) and colistin, at a range of charge fractions (*f*_+_) of 0.09 ≤ *f*_+_ ≤ 0.98 at a formation concentration of 5.0 mg/mL. The stability, measured by follow-up measurements over time with DLS (Figure S1), is indicated by the color coding of the background. 0–1-day(s) stability (red), 2–7-day stability (orange), 8–28-day stability (yellow), and > 28-day stability (green) are separated in the background. To improve visibility by creating sharp color borders a color spacing of 0.02–0.05 was taken horizontally.

In which *Z*_Col_ is the number of charges from colistin in the formulation and *Z*_Poly_ is the number of charges from PEO-*b*-PMAA in the formulation. For P1, we could prepare colistin complex coacervates for 0.09 ≤ *f*_+_ ≤ 0.98 at a total concentration of 5.0 mg/mL. Outside of this region, no complex coacervates were formed, as confirmed by DLS. To investigate the nature of these complex coacervates the ζ-potential was measured, right after mixing and combined with measured stability and sizes by DLS in Figure 1. The stability is based on the number of days after complexation after which aggregation was apparent, as seen in Figure S1 in the Supporting Information.

The region of 0.50 ≤ *f*_+_ ≤ 0.83 was found to be (size-)stable over a long term, i.e., more than 28 days, as indicated by the green background (Figure 1). The addition of colistin charge does not seem to affect the ζ-potential in the same way the addition of anionic P1-charge does. At *f*_+_ < 0.50, the ζ-potential deviates further from zero, resulting in quicker aggregation (from micro to macro phase separation). Therefore lower stability is observed, already visible by the presence of larger sizes at *f*_+_ < 0.50, right after mixing. In the stable region, the ζ-potential is closer to zero, while complex coacervates with a size of 35–40 nm are found. This indicates the beneficiality of complex coacervate neutrality in the system at a particular size to achieve the most stable systems. Here, the PEO corona is sufficient to provide colloidal stability via entropic (steric) repulsions between overlapping polymer brushes, like in other C3M formulations.^61,62^ It does not hold for *f*_+_ > 0.83. Even though the effective charge, the ζ-potential, is close to zero, the systems destabilized quickly (within two days). This is probably due to excess colistin charge, disrupting the homogeneous charge and resulting in the initialization of aggregation after mixing. At these charge fractions, the ionic strength might have exceeded the threshold energy for long-term stability in the systems, causing aggregation, even though the ζ-potential remains at zero when measured. The ζ-potential behavior for a wide range of *f*_+_ values differs from complex coacervate system to system.^27^ Therefore, the ζ-potential and DLS sizing alone do not give enough information about the charge fractions in the system to conclude on compositional characteristics.

P2-colistin and P3-colistin showed the same trends in stability, with a stable charge fraction region, but showed to have formed complex coacervates in a much smaller window of charge fractions, for P2 0.33 ≤ *f*_+_ ≤ 0.83, and for P3 0.50 ≤ *f*_+_ ≤ 0.83 (Figures S2 and S3). Additionally, the larger block lengths led to larger colistin complex coacervates at all charge fractions, with the most pronounced size change observed at the long-term stable conditions for all polymers at *f*_+_ = 0.50 (Table 1).

**Table 1:** Hydrodynamic Radii (*R*_*h*_) of Complex Coacervates from P1, P2, and P3 with Colistin at Long-Term Stable Conditions at *f*_+_ = 0.50, Measured by DLS with CONTIN Fits at 120°

polymers used for complexation with colistin at *f*_+_ = 0.50	*R*_h_ (DLS, averaged based on CONTIN fits at 120°, Figure S4) (nm)
P1 (PEO_45_-*b*-PMAA_41_)	18 ± 1
P2 (PEO_45_-*b*-PMAA_81_)	77 ± 3
P3 (PEO_114_-*b*-PMAA_81_)	87 ± 2

**Figure 2:**
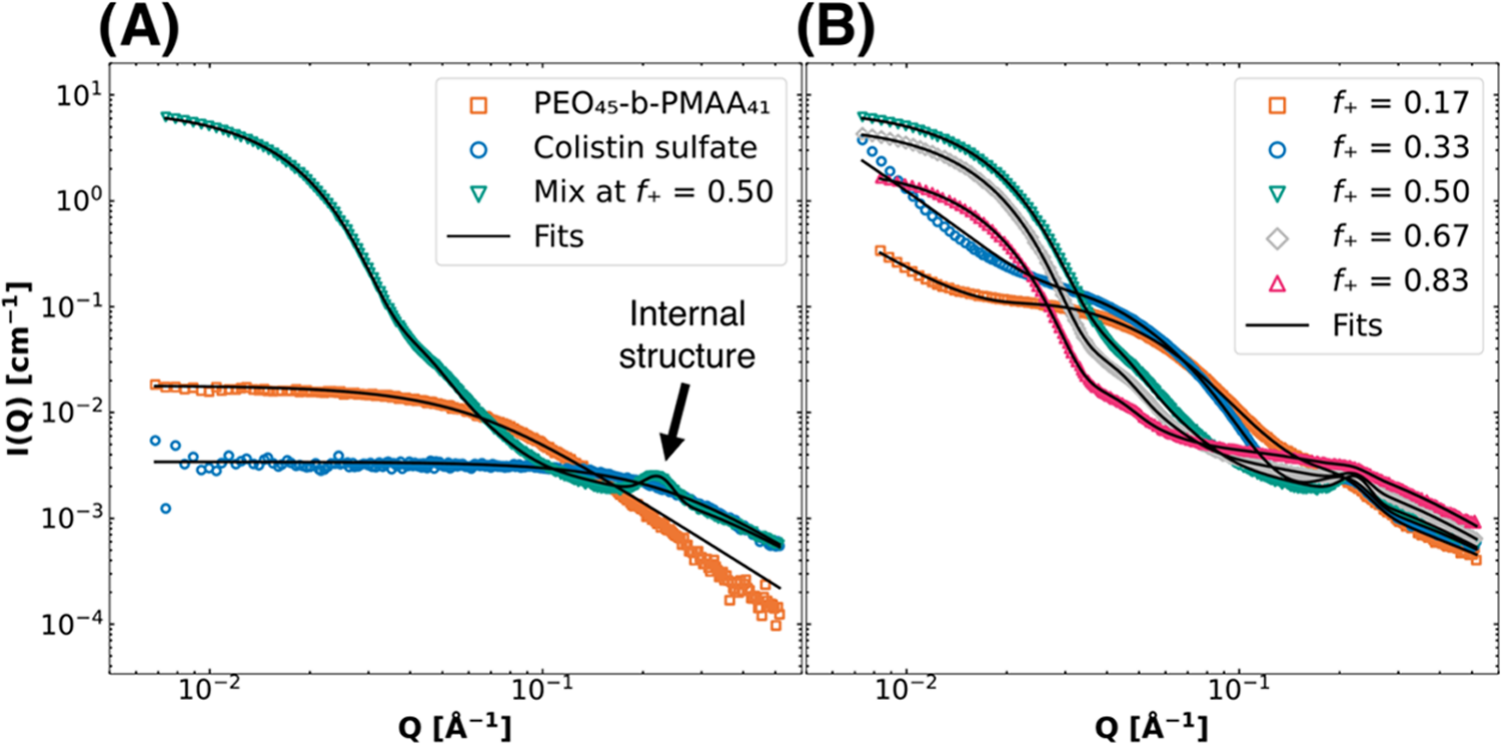
SAXS profiles of PEO_45_-*b*-PMAA_41_ (orange squares) at 1.6 mg/mL colistin sulfate (blue circles) at 3.4 mg/mL, and colistin complex coacervates at *f*_+_ = 0.50 (green triangles (pointing down)) at 5.0 mg/mL total concentration. The lines depict the results of fit analysis using the Debye model for PEO_45_-*b*-PMAA_41_ and colistin sulfate and the fuzzy-surface complex coacervate model for colistin complex coacervates (A). Scattering profiles are depicted of complex coacervates at *f*_+_ = 0.17 (orange squares), *f*_+_ = 0.33 (blue circles), *f*_+_ = 0.50 (green triangles (down)), *f*_+_ = 0.67 (gray diamonds), and *f*_+_ = 0.83 (pink triangles (up)) at a total concentration of 5.0 mg/mL including fitted curves (B). Scattering patterns in B are fitted using the fuzzy-surface complex coacervate model for the different charge fractions of complex coacervates.

The size and shape of nanoparticles used in therapeutics play a crucial role in their bioavailability and biochemical stability once they enter the bloodstream. Therefore, we need to consider the potential threat of spleen and liver accumulation for larger particles (above 100 nm diameter), as well as the renal filtration systems that clear up free unimers from unstable complex coacervate formulations.^63−66^ Based on its smallest size and largest stability region, we think P1-colistin complex coacervates would have the most promising therapeutic potential. Hence, they were analyzed further on their charge dependence and compositional characteristics using SAXS. SAXS provides possibilities to analyze and characterize samples in solution at the nanometer scale, allowing resolution of the shape, size, composition, and structure.^67^ The SAXS profiles of colistin complex coacervates at *f*_+_ = 0.50 (total concentration of 5.0 mg/mL) and the scattering profiles of their separate components are depicted in Figure 2A. In Figure 2B, the colistin complex coacervates in five different charge fractions (total concentration of 5.0 mg/mL), and their fits are shown. We analyzed the SAXS profiles of the complex coacervates using least-squares fit routines with a custom-designed fuzzy-surface complex coacervate model with graded interfaces (see the Experimental Section). The most important parameters fitted to the data were the aggregation number (*P*), the radius of the core (*R*_in_), the width of the interface of the core (σ_in_), the free fraction of colistin outside of the micelles (*f*_Col_) and the polydispersity index of the micelles (PDI). Based on these parameters, the concentration of encapsulated colistin (*c*_ColC3M_), the total radius of the core (*R*_tot_), molecular weight (*M*_w_), and volume fraction of water in the micelle (*f*_w_) could be calculated (calculations are given in the Supporting Information). Noncomplex coacervate structures were analyzed using fit models using either the Debye form factor for polymers and polyelectrolytes (Figure 2A) or the prolate/oblate ellipsoid form factor for globular proteins (Supporting Information).

Generally, in complex coacervation, three phases can be distinguished based on the charge fractions.^27,34^ First, the phase in which there is no formation of complex coacervates (single chains), second, the phase in which excess charge causes the formation of either anionic or cationic soluble complex particles (SCPs) and third, the phase where C3Ms are present. To figure out this phase behavior and to understand the colistin-C3M charge dependence, SAXS data was analyzed using the model in the whole range of 0.09 ≤ *f*_+_ ≤ 0.98, a larger range than the data shown in Figure 2B. The most relevant fit parameters of the results are summarized in Table 2.

**Table 2:** Most Relevant (fit) Parameters, Based on Fitting of Different Charge Fractions for C3Ms 0.09 ≤ *f*_+_ ≤ 0.98 Using the Fuzzy-Surface Complex Coacervate Model^a^

*f*_+_	*P*·10^3^	*R*_tot_ (nm)	σ_in_ (nm)	*f*_Col_	*c*_ColC3M_ (mg/mL)	*M*_w_ (Da)·10^6^	*f*_w_
0.09	0.16	3.8	0.0	0.02	0.8	0.26	0.0
0.17	0.15	4.1	0.0	0.07	1.4	0.25	0.0
0.23	0.15	4.2	0.0	0.07	1.8	0.26	0.0
0.33	0.12 ± 0.05	6.9 ± 0.9	3.0 ± 0.4	0.07 ± 0.01	2.36 ± 0.03	0.19 ± 0.08	0.27 ± 0.03
0.44	1.9 ± 0.6	15 ± 1	1.2 ± 0.4	0.10 ± 0.03	2.81 ± 0.08	3 ± 1	0.59 ± 0.06
0.50	1.4 ± 0.2	15.7 ± 0.3	1.7 ± 0.1	0.16 ± 0.01	2.84 ± 0.01	2.3 ± 0.4	0.74 ± 0.03
0.55	1.0 ± 0.1	15.6 ± 0.1	1.7 ± 0.1	0.21 ± 0.01	2.81 ± 0.04	1.6 ± 0.1	0.78 ± 0.02
0.60	1.2 ± 0.1	15.8 ± 0.2	1.9 ± 0.2	0.38 ± 0.02	2.34 ± 0.07	1.9 ± 0.2	0.80 ± 0.02
0.64	1.2 ± 0.1	16.2 ± 0.6	1.9 ± 0.2	0.48 ± 0.03	2.1 ± 0.1	2.0 ± 0.2	0.80 ± 0.01
0.67	1.12 ± 0.04	16.2 ± 0.2	2.0 ± 0.2	0.54 ± 0.01	1.86 ± 0.02	1.9 ± 0.1	0.78 ± 0.01
0.75	1.3 ± 0.1	16.3 ± 0.4	2.0 ± 0.3	0.71 ± 0.01	1.23 ± 0.05	2.1 ± 0.2	0.75 ± 0.01
0.83	1.5 ± 0.6	16.5 ± 0.3	2.1 ± 0.3	0.82 ± 0.01	0.84 ± 0.01	2 ± 1	0.80 ± 0.08
0.91	1.3 ± 0.2	17 ± 1	2 ± 2	0.91 ± 0.01	0.45 ± 0.02	2.2 ± 0.4	0.80 ± 0.01
0.98	1.4	19	3.8	0.98	0.08	2.4	0.72

a Averages were taken from the fits at three concentrations for *f*_+_ ≥ 0.33, while for *f*_+_ < 0.33 and *f*_+_ > 0.91, only 5.0 mg/mL was possible to be fitted. Since the concentration of colistin is also lower at lower total concentrations, the *c*_*ColC3M*_ decreases as well, but for comparison, the lower concentrations are scaled (normalized) to 5.0 mg/mL.

The data for charged matched mixtures are shown in Figure 2A and demonstrate the formation of spherical-like micelles upon mixing. While the two individual components display low-intensity scattering with a weak *Q*^–2^ dependence at high *Q*, the scattering curve from the mixed coacervate is much more pronounced with a Guinier plateau at low *Q* and steep decay at intermediate *Q* resembling spherical entities. In addition, there is a clear correlation peak at high *Q*, which indicates an optimal packing distance between the colistin and PMAA chains within the core. This has been found in fully polymeric coacervates and attributed to positional correlations between oppositely charged electrostatic blobs.^59^ This feature is the most pronounced feature at *f*_+_ = 0.50 and gradually washed out at off-stoichiometric charge balances. For mixtures with *f*_+_ < 0.50 (Figure 2B), we also observe upturns at low Q, revealing the formation of micellar clusters. To describe this, a cluster structure factor, based on a fraction of clustered micelles placed at a certain distance from each other, was used (Supporting Information). For 0.33 ≤ *f*_+_ ≤ 0.91, fits were obtained at three different concentrations (5.0, 2.5, and 1.3 mg/mL) and could be normalized and averaged because of apparent concentration independence within the experimental error limit (Figure S5, all fits found in Tables S1–S3). Outside of this region, it was not possible to measure at lower concentrations than 5.0 mg/mL because of the increased instability of the system.

The highest concentration of colistin in the complexed fraction (*c*_ColC3M_) was found at charge-matching conditions, close to *f*_+_ = 0.50, and decreasing in both directions further away from charge matching (Table 2). The presence of more anionic charge seems to affect the size and molecular weight of C3Ms, especially in the 0.09 ≤ *f*_+_ ≤ 0.33 region, where polymer charges are in excess. The particles formed in this region are smaller and contain fewer molecules, but from DLS we also detect larger aggregates (Figure 1). In theoretical models for complex coacervates, this region is also known as the anionic soluble complex particle (SCP^–^) region.^27^ The border between SCP^–^ and C3Ms is referred to as the critical excess anionic charge (CEAC) point, and in the case of P1-colistin-C3Ms, the CEAC lies between *f*_+_ = 0.33 and *f*_+_ = 0.44,^27,34^ which is clearly shown from the increase of *f*_w_. SCPs cannot contain a high volume of water since they are small and negatively charged, whereas C3Ms are stable because of the large volume fraction of water.

**Figure 3:**
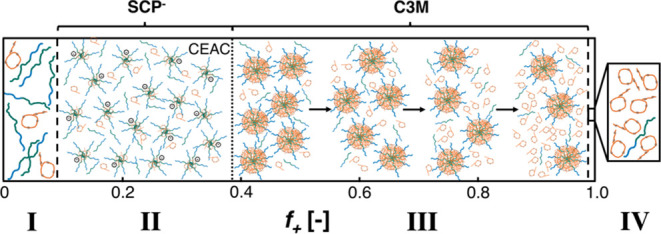
Graphical illustration of the effect of charge fractions on the formation of complex coacervates. At *f*_+_ < 0.09 (I), no complex coacervates are formed, followed by the SCP-phase (II), in which negatively charged small structures are present until the CEAC point between *f*_+_ of 0.33 and 0.44. After the CEAC, C3Ms are formed (III), with a decreasing fraction forming micelles the further you go up the scale. From *f*_+_ = 0.98 (IV), again, no complex coacervates are formed. Illustrations are not scaled.

C3Ms are present in the 0.44 ≤ *f*_+_ ≤ 0.98 region, and even when there is an excess of colistin charge, the aggregation number, size, molecular weight, and water volume fraction of the C3Ms, and the width of the fuzzy surface (σ_in_) remain consistent (confirmed by the density profiles shown in Figure S6). However, the fraction of free colistin exhibits a negative correlation with *f*_+_. At higher *f*_+_-values, there is a sharp cutoff from C3Ms to no structures (around *f*_+_ = 0.98). This is an indication that there is no SCP^+^ region and consequently, no critical excess cationic charge (CECC) point. The further away from charge matching conditions, we either observe more aggregates and smaller particles (*f*_+_ < 0.50) or lower encapsulation efficiency (*c*_ColC3M_) (*f*_+_+ > 0.50). These findings are summarized and illustrated in Figure 3.

As illustrated in Figure 3, from left to right, four phases can be distinguished. In the first phase (I) where the polymer charge is in excess, the polymer and colistin do not form any complexes but are rather present as single chains. In the second phase (II), we have the negatively charged soluble complex particles that are smaller than C3Ms, in which the polymer charge is in excess. The third phase (III), after the critical excess anionic charge (CEAC) point is crossed, is the C3M phase. The largest number of C3Ms with the lowest free fraction of colistin can be found at charge matching at 16%. At higher *f*_+_ values, the number of C3Ms decreases while the free fraction of colistin increases. In the fourth phase (IV), the polymer and colistin do not form complexes, but in this case, the colistin charge is in excess. Considering the focus on stability and toxicity decrease, charge-matching conditions (*f*_+_ = 0.50) were taken further into characterization, as this system is stable and has the lowest free fraction of colistin (Figure 3). In addition to these observations, it has to be considered that C3Ms generally are dynamic systems.^27,34,35,68^ In the circumstances in which these C3Ms are formed, the micelles are in a kinetically arrested state. However, the metastable C3Ms could be affected by changes in pH, altering the effective charge, addition of ionic strength, or dilution of systems, which are parameters that are especially relevant in biological settings. For many different C3Ms formulations, this behavior is intrinsically different.^27,68^ To assess the effect of ionic strength changes and pH changes, we exposed the P1-colistin-C3Ms at *f*_+_ = 0.50 to increased ionic strength of physiological levels during mixing (Figure S7, Table S4) and after mixing (“salt annealing”) (Figure S8, Table S5), and mixed P1-colistin-C3Ms at several different pH values between 5.0 and 8.7 (Figure S9, Table S6). We only found a small effect of increasing ionic strength in both mixing and after mixing within physiological ionic strength levels (Figures S7 and S8). Upon gradual addition of 0.30 M of NaCl, the P1-colistin-C3Ms swells (higher *f*_w_, while *R*_tot_ increased, and *P* remained the same) and became generally more polydisperse, which was expected from the increased screening of charges.^27^ After the addition of 0.30 M NaCl and at 0.50 M NaCl, the C3Ms disintegrate into smaller micelles with lower aggregation numbers. Decreasing the pH caused an increase in polydispersity, as well as a small decrease in size and aggregation, and an increase in the free fraction of colistin (from 16% at pH = 7.4) to 60% at pH 5 and 6. Most likely because of the decreased charge of the PMAA block, which is known to have a p*K*_a_ between 4 and 5.^69^ Less charge in the system from polymer will lead to a decrease in colistin complexation. An increase in pH resulted in the same colistin-C3Ms found at physiological pH (pH = 7.4), with a slightly increased aggregation number and decreased polydispersity.

To further investigate the stability toward dilution, we investigated the (apparent) critical micelle concentration (CMC) with a pendant drop tensiometer (Figure S10).^27,70^ Above the CMC, the C3Ms are in an effectively arrested state, while below the CMC, the C3Ms may dissociate into their corresponding compounds. The CMC was found to be around 0.3 mg/mL. SAXS data confirms these findings since around concentrations of 0.3 mg/mL, micelles start dissociating (Figure S11). Since we have established the boundaries of the kinetically arrested state and found the most optimal formulation based on stability, structure, size, and compositional characteristics, we can begin assessing their therapeutic potential. Characterization of *f*_+_ = 0.50 colistin-C3Ms encompassing their antimicrobial, enzyme protection, release, serum binding, and toxicity properties was performed.

### 3.2 Antimicrobial Properties

**Figure 4:**
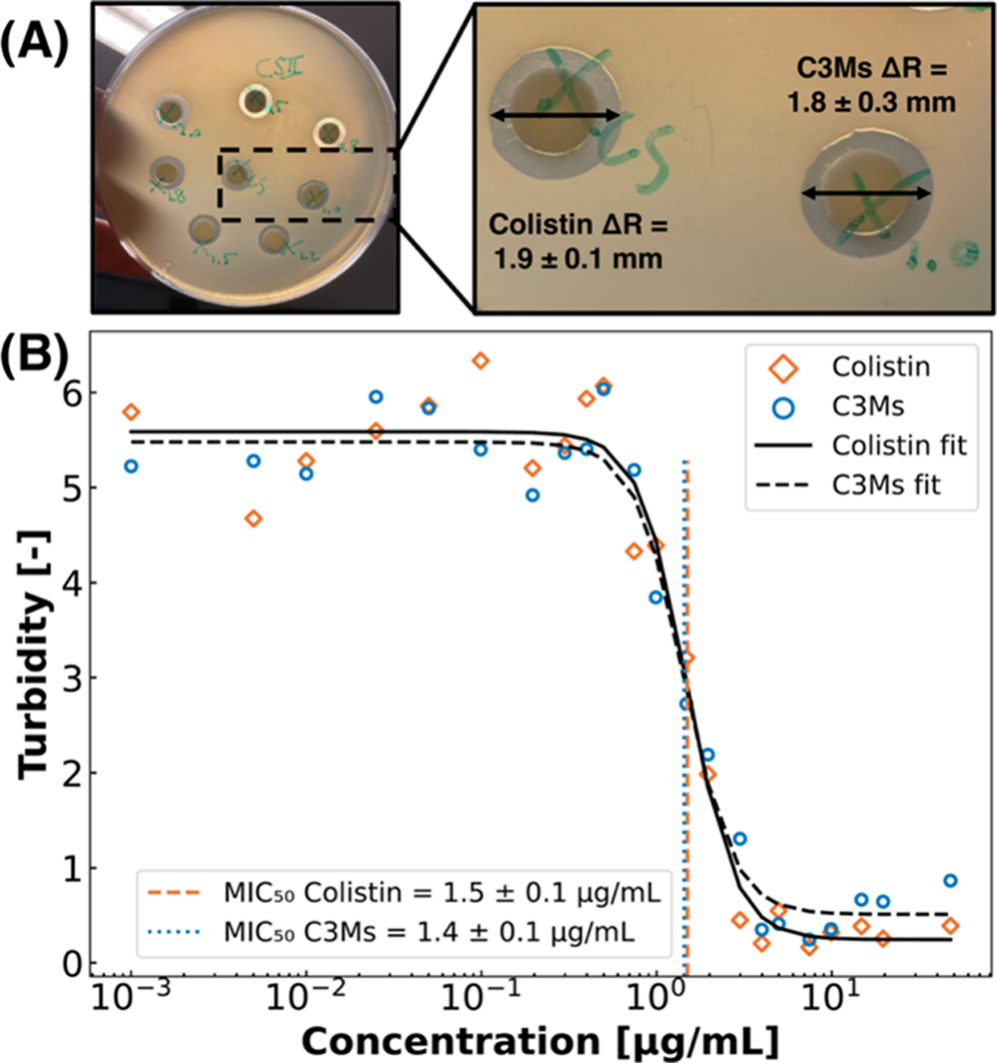
Disk diffusion assay (DDA) of colistin and colistin-C3Ms with 3.0 μg of colistin in the *E. coli* agar medium (A). The inhibition zones were measured, averaged, and compared. MIC_50_ determination of colistin (orange diamonds) and colistin-C3Ms (blue circles) on *E. coli* using the agar dilution method (B). The turbidity was plotted against the concentration, which was plotted on a logarithmic scale to improve visibility. The colistin (black line) and colistin-C3Ms curves (black dashed line) were fitted with a sigmoid function from which the MIC_50_ values were determined, which are plotted in the figure (orange dashed line for colistin and blue dotted line for colistin-C3Ms).

To compare the antibiotic properties of the C3Ms versus free colistin, first, a disk diffusion assay (DDA) was performed. In the DDA the susceptibility of *E. coli*, *Serratia indica*, *P. fluorescens*, *A. vinelandii*, and *B. subtilis* as a control (Figure S12) were tested in the presence of colistin and colistin-C3Ms.^71,72^ The best results were obtained for *E. coli* (Figure 4A) in the conditions that were assessed, since *S. indica* grew back over the inhibition zones (Figure S12), while *P. fluorescens* was not visibly affected, and *A. vinelandii* showed immeasurable small inhibition zones. Second, the MIC_50_ values (minimum inhibitory concentrations) for both formulations were determined in *E. coli* culture, to see whether there is a difference in susceptibility based on the introduction of polymer in the bacteria growth medium.^71^ To determine the MIC_50_, the turbidity was measured, with higher turbidity indicating increased bacterial proliferation, after which a sigmoid was fitted to the data (Figure 4B).

There were no significant differences between colistin and colistin-C3Ms in the susceptibility of *E. coli* for both experiments (Figure 4). In the DDA, the sizes of inhibition zones were respectively 1.9 ± 0.1 and 1.8 ± 0.3 mm for colistin and colistin-C3Ms (Figure 4A), while the MIC_50_ values were 1.5 ± 0.1 and 1.4 ± 0.1 μg/mL respectively (Figure 4B). The MIC_50_ values measured were in the expected range for colistin, as values between 0.5 and 8.0 μg/mL have been reported before in different assay conditions.^73,74^ In the MIC_50_ determination, the concentrations tested were all below the CMC, meaning no C3Ms were present in the agar samples. Hence, only the effect of the addition of polymer could be assessed, and based on Figure 4B, the polymer did not seem to affect the antimicrobial properties of colistin. Since there are no significant differences between colistin and C3Ms in the DDA, the C3Ms likely disintegrate, releasing the colistin in the process, resulting in no losses in antimicrobial activity. However, another option is that the micelles stay intact and exert their antimicrobial effect as particles, even though this is less likely since C3M systems are susceptible to dilution over the whole agar plate. In addition, Kourmouli et al.^72^ found that nanoparticles of similar size containing antibiotics have a significantly decreased diffusivity, and if the antibiotics are not released, no antimicrobial effect could be observed.

### 3.3 Enzymatic Breakdown Susceptibility

**Figure 5:**
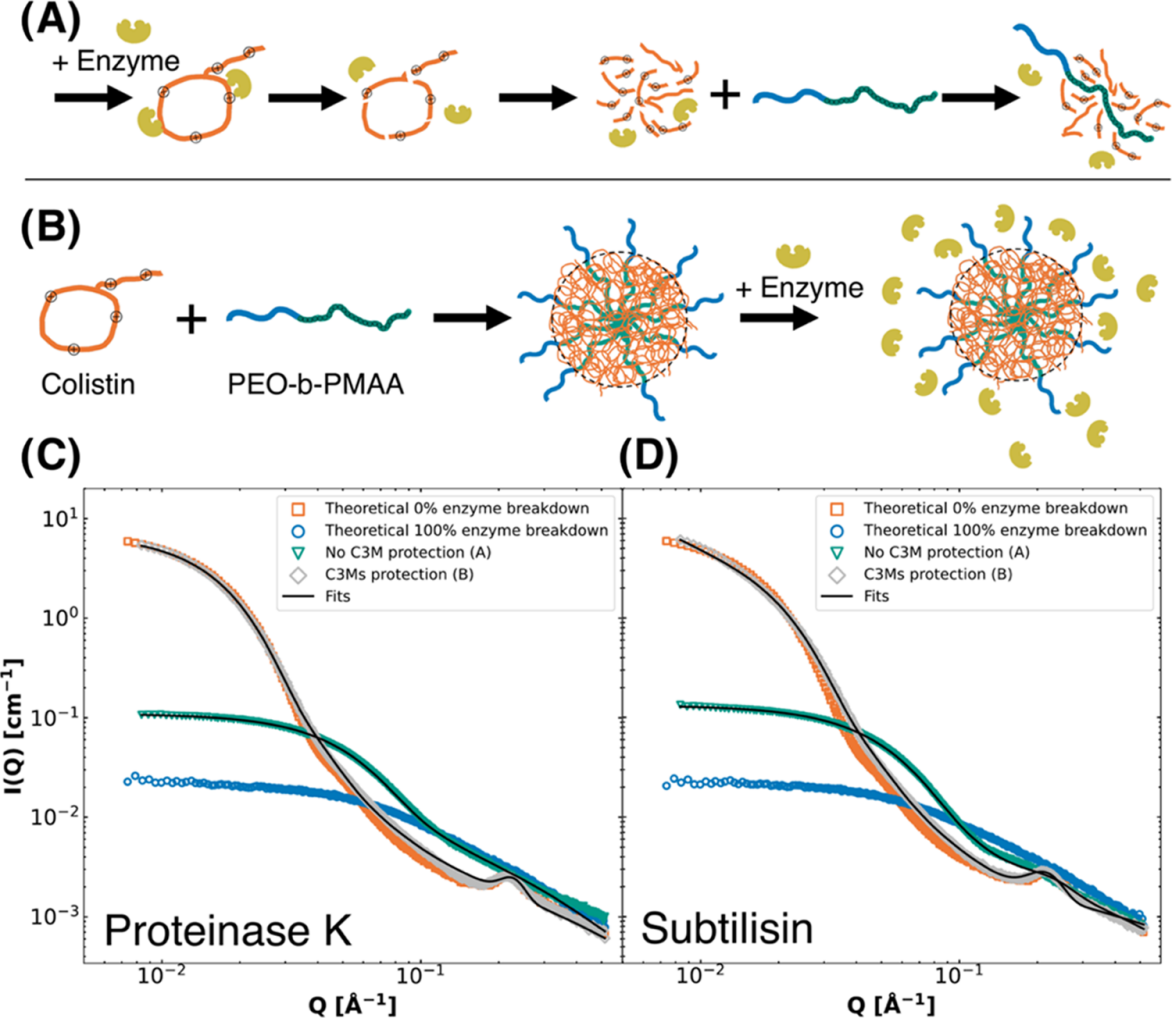
Illustrations of hypothesized enzymatic breakdown comparisons (A, enzyme attacks colistin and then undergoes complexation, and (B) enzyme attacks after complexation) and SAXS patterns of enzymatic breakdown of colistin (3.4 mg/mL) versus colistin-C3Ms (total concentration of 5.0 mg/mL) in the presence of proteinase K (C) and subtilisin (D). Theoretical SAXS patterns were calculated based on either the added scattering from C3Ms and enzymes (0% enzyme breakdown, orange squares) or colistin, polymer, and enzyme separately summed up (100% breakdown, blue circles) (C, D). The effect of enzymatic degradation after 24 h at 37 °C was measured by either adding enzyme to colistin, followed by complexation with polymer (illustrated in A, and the graphs in C and D, indicated by green triangles) or addition of enzyme to C3Ms (illustrated in B, and the graphs in C and D, indicated by gray diamonds). The SAXS patterns were fitted using the fuzzy-surface complex coacervate model for complex coacervates.

Generally, AMPs, and therefore also colistin, are known for their low *in vivo* stability due to protease degradation.^26,75^ The complexation of colistin could be advantageous if it improves the retainability and stability, by protecting the drug from degradation by enzymes. To assess the enzymatic susceptibility of colistin versus colistin-C3Ms in concentrations above the CMC, two peptidases with a broad specificity were used: proteinase K and subtilisin. Trypsin was also assessed but was found ineffective in breaking down colistin (Figure S13). To visualize the hypothesized effect and experimental design of colistin breakdown for colistin and colistin-C3Ms, an illustration was made (Figure 5A,B). The enzymes were either added to colistin, then incubated, and then complexed with polymer (Figure 5A), or the enzymes were added to the C3Ms and then incubated (Figure 5B). The SAXS patterns are shown in Figure 5C,D and then compared to theoretical, noninteracting (calculated) enzymatic breakdown SAXS patterns. These theoretical SAXS patterns were calculated by simple addition of the scattering of each component separately (polymer, colistin, and the small contribution of enzyme) to resemble 100% enzyme breakdown, called “No C3M protection (A)”. Equivalently, the theoretical scattering curves from C3Ms added to the negligible enzyme scattering (resembling 0% enzyme breakdown) are called “C3M protection (B)” (Figure 5C,D). The data from both methods was analyzed using the fuzzy sphere complex coacervate model and compared to regular C3Ms (Table S7).

From the fits, for proteinase K degradation, the molecular weight of C3Ms changed from *M*_w_ = 2.3 ± 0.4 MDa to *M*_w_ = 0.13 MDa without C3M protection and *M*_w_ = 2.1 MDa with C3M protection. For subtilisin degradation, the decrease in molecular weight of the complex coacervates was more extensive. The molecular weight dropped from *M*_w_ = 2.3 ± 0.4 MDa to *M*_w_ = 0.042 MDa without C3M protection and to *M*_w_ = 1.1 MDa with C3M protection. Therefore, it is apparent that the C3Ms protect colistin from enzymatic breakdown, while in a noncomplexed state, colistin is prone to enzymatic degradation by both proteinase K and subtilisin (Figure 5). Proteinase K can cleave colistin such that the average molecular weight of colistin complex coacervates was reduced by a factor of ≈ 20, while subtilisin breakdown results in a molecular weight reduction of ≈ 50 times. Subsequently, C3Ms seem unaffected by proteinase K, while subtilisin caused a reduction of approximately factor two in molecular weight. In conclusion, C3Ms seem to have a comparable protection effect for both enzymes, compared to noncomplexed colistin: enzymatic breakdown reduction by a factor of ≈20 for its molecular weight. Furthermore, complexation was found to be beneficial to the enzymatic stability and potentially the retainability of colistin. Nevertheless, it must be noted that these studies do not indicate the colistin activity after enzymatic degradation, even though the C3Ms stay intact. However, the retainability of colistin, if improved, can reduce the quantities of colistin needed for treatment, lowering its dose, and reducing the side effects of colistin.

### 3.4 Human Serum Albumin (HSA) Binding

**Figure 6:**
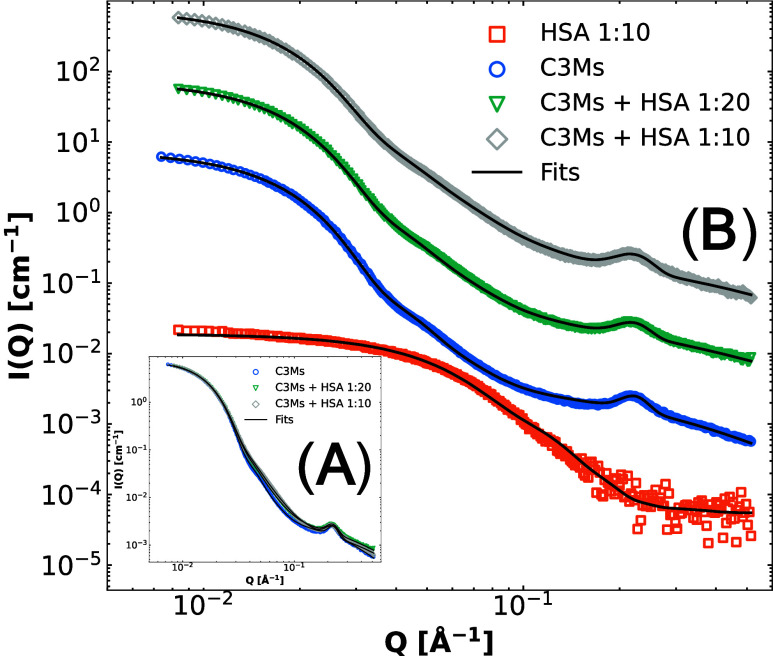
SAXS patterns of C3Ms (blue circles) and C3Ms with added HSA at a ratio of 1:10 HSA:colistin (green triangles) and 1:20 HSA:colistin (gray diamonds) are presented in (A). To better visualize the effect of HSA addition, we show HSA by themselves (orange squares) and C3Ms by themselves (blue circles) in (B), as well as the scattering patterns in which HSA is combined with C3Ms separated by multiplication of the *I*(*Q*) with a factor of 10 for 1:20 HSA:colistin (green triangles) and 100 for 1:10 HSA:colistin (gray diamonds) to increase the visibility of structural changes. The SAXS patterns were fitted using the fuzzy-surface complex coacervate model for complex coacervates with added prolate/oblate ellipsoidal scattering of HSA if present.

Next to enzymatic destabilization causing low retainability, (serum) protein binding is another factor that can negatively affect colistin’s *in vivo* stability.^26,27,62,68^ Since colistin-C3Ms would be transported in the bloodstream, it can interact with several different (small) proteins that can bind to colistin or the PEO-*b*-PMAA and affect the encapsulation effectivity of the colistin-C3Ms. To investigate whether the C3Ms are structurally affected by the presence (of high levels) of serum proteins, HSA was added in two different molar ratios of 1:10 and 1:20 (HSA:colistin) and measured using SAXS (Figure 6). Fits were again performed using the fuzzy-surface complex coacervate model, in which the scattering of HSA was included by adding the form factor of a prolate/oblate ellipsoid to the model.

Through the model fits, it was possible to extract the effect of HSA showing only a minor effect on the structure of C3Ms. The total radius was reduced from 15.7 ± 0.3 to 13.8 ± 0.3 nm, while the polydispersity index was essentially the same, 18% and 20% before and after exposure of HSA up to 1:10 (mole ratio). The colistin-C3Ms thus stay intact with a similar aggregation number (*P*) and molecular weight, while the free fraction of colistin also remained around 16% (Table S8). A possible reason for the size reduction could be the increase of total charged polyelectrolytes in the system, leading to a reduction of water in the core because of osmotic flow.^76,77^ This results in a slight condensation of the colistin and polymers in the core.^27,77^ Even though the size of the C3Ms was changed significantly, the micelles stayed intact with the same composition, showing promising drug delivery characteristics.

### 3.5 Toxicity

**Figure 7:**
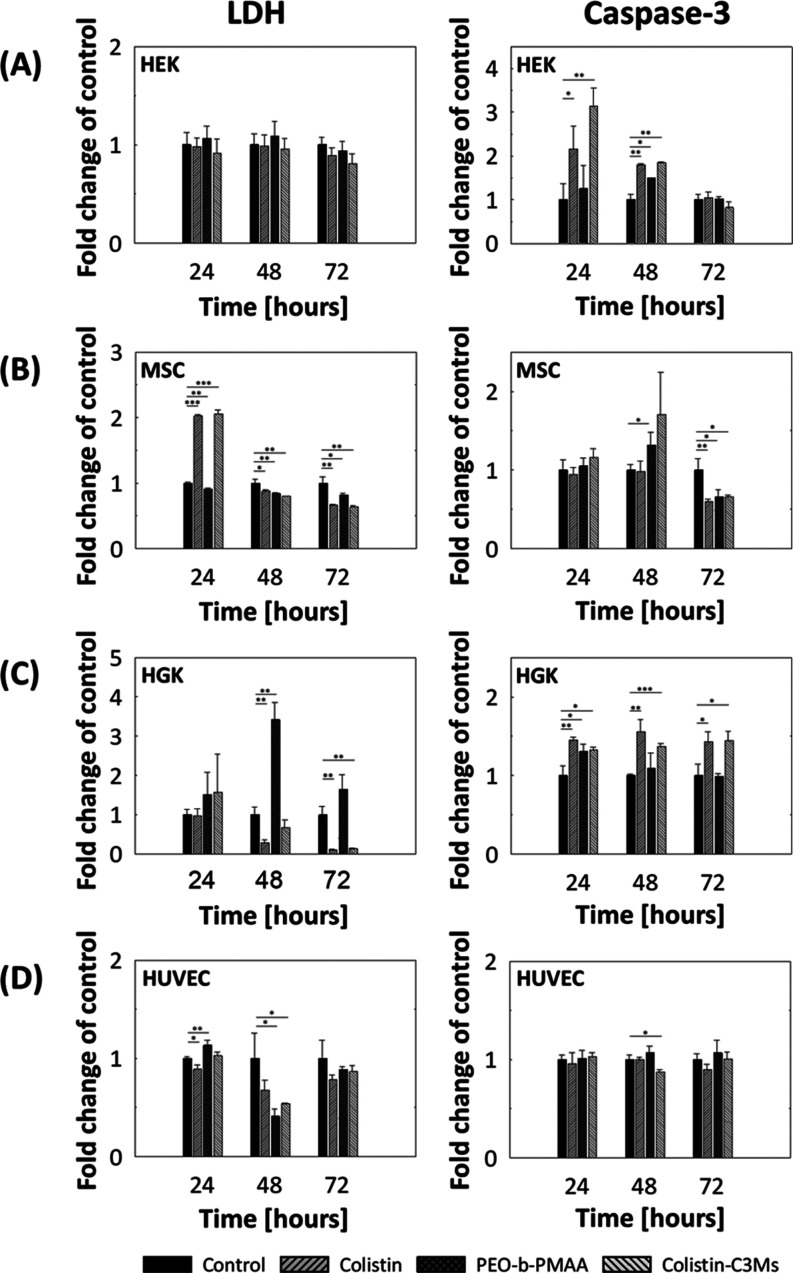
Cell viability of human embryonic kidney 293 (HEK) cells (A), mesenchymal stem cells (MSCs) (B), human gingival keratinocytes (HGKs) (C), or human umbilical cord endothelial cells (HUVECs) (D) treated with colistin, PEO-*b*-PMAA, or colistin-C3Ms. Lactate dehydrogenase (LDH) and caspase-3 activity were measured in the corresponding cell culture medium, expressed as fold change of control at 24, 48, and 72 h. Data are presented as the mean effect of 3 parallel cellular experiments for each stimulation at each time point. Significantly different from untreated control cells at each time point at *p < 0.05, **p < 0.01, and ***p < 0.001.

The level of cytotoxicity indicates the availability of colistin in cellular environments and is an indication of the protection level of the cargo.^28,78^ With the quantification of the toxicity level of colistin and colistin-C3Ms, we could find out what cell types are most prone to colistin toxicity, as well as compare how colistin and encapsulated colistin affect the cytotoxicity of living cells. To measure the cytotoxicity of colistin-C3Ms and noncomplexed colistin, four different cell types were investigated that all have relevance to the nephrotoxic properties of colistin. We investigated human embryonic kidney 293 cells (HEK), an immortalized cell line derived from human embryonic kidney cells. Second, we investigated human mesenchymal stem cells (MSC), multipotent cells that can be isolated from several tissues, including the kidney.^79^ The third cell type was human gingival keratinocytes (HGK). They play an important role in promoting strong epithelial bonds, which are highly relevant for renal physiology.^80^ Lastly, human umbilical cord endothelial cells (HUVEC) were tested, which are generally used as a model for vascularized tissues, like the kidneys.^81^ MSC, HGK, and HUVEC were exposed to freeze-dried colistin-C3Ms to facilitate addition to cell cultures (freeze-drying effect: Figure S14) and noncomplexed colistin. To assess the toxicity, the lactate dehydrogenase (LDH) level and caspase-3 level were measured with two colorimetric bioassays (Details in the Experimental Section). LDH is a biomarker for necrosis,^82^ whereas caspase-3 is a well-described protease that plays a role in programmed cell death (apoptosis).^83^ LDH and caspase-3 were measured after 24, 48, and 72 h of exposure (Figure 7), and light microscopy was used to image the cell morphology (Figure S15).

Based on LDH and caspase-3 measurements, colistin, and colistin-C3Ms were found to have a different effect on the various cell types tested, but in general to be mildly toxic for all cell types (Figure 7). For HEK 293, the caspase-3 levels were up to 3-fold increased on day one and 2-fold increased on day two for both colistin and colistin-C3Ms, while there were no significant differences in LDH activity (Figure 7A). For MSC, the LDH activity was found to be 2-fold increased after 24 h and reduced in the later days because of necrosis-induced dead cell detachment,^82,84^ while there was no significant difference in caspase-3 levels after 24 h (Figure 7B). Subsequently, MSC cell death was likely caused by increased necrosis from colistin and colistin-C3M treatments. Colistin, and colistin-C3M both caused necrosis (LDH) and apoptosis (caspase-3) of HGK after 24 h (Figure 7C). Notably, PEO-*b*-PMAA caused an unexpectedly high spike in LDH level after 48 h as PEO and PMAA are generally found to have low toxicities^85,86^ (Figure S15). Lastly, in HUVEC cells, there was no significant effect for any treatment in the first 24 h (Figure 7D). However, after 48 h, a decrease in both LDH and caspase-3 values was observed. These findings are most likely a result of lower cell proliferation or a result in cell arrest of the treated HUVEC cells compared to untreated cells, rather than a toxic effect. This might indicate a higher resistance of this cell type, compared to MSC and HGK.

Generally, for all cell types, we found that the cytotoxicity of colistin-C3M was not lower than that of colistin itself. Most likely this is caused by cell-drug interactions with colistin and C3Ms′ colistin release during the 24 h. Even though no direct reduction of toxicity was found from complexation, it is another indication, next to the antimicrobial effect that the colistin is still active. In any case, more testing needs to be considered on cytotoxicity, *in vivo* stability, and drug effectivity to validate the viability of colistin-C3Ms for actual *in vivo* purposes.

## 4 Conclusions

In this work, we demonstrated the preparation of PEO-*b*-PMAA-colistin-C3Ms that are highly stable and reproducible. Scattering techniques (DLS and SAXS) were employed to analyze the structure and stability of colistin complex coacervates at different charge fractions. Using a detailed SAXS model, built up of three scattering contributions: complex coacervate scattering, free component scattering, and an internal structure factor from the polyelectrolyte core, it was possible to quantify the complex coacervates on their structure, molecular weight, size, and composition. Based on the fit analysis the highest therapeutic potential of colistin complex coacervates was found to be at polyelectrolyte charge-matching conditions. C3Ms at this charge fraction exhibited the highest colistin complexation efficiency while being long-term stable. Subsequently, this formulation was assessed on several properties that are important for clinical purposes, like ionic strength effect, pH dependency, CMC, antibiotic properties, (enzymatic) stability, serum protein binding, and toxicity. The C3Ms demonstrated similar antibiotic effectiveness and equivalent toxicity compared to noncomplexed colistin, while the complexation of colistin into C3Ms was found to improve the protection against enzymatic degradation. Moreover, the presence of HSA had a negligible impact on C3Ms, which remained intact upon HSA addition. Comparing C3Ms and noncomplexed colistin, we found a similar antimicrobial/toxic effect, low HSA binding, and increased enzymatic stability. We believe the complexation of colistin in C3Ms could still be a viable strategy to reduce the total toxicity since the retention of colistin in complexed form is improved providing the possibility of reducing the concentration of the administered drug. Consequently, our study suggests that C3Ms incorporating colistin might offer a novel addition to the array of antibiotic formulations suitable for e.g., topical treatments in clinical applications.

## Supporting Information Available

Details of the theoretical SAXS data modeling; DLS, including autocorrelation functions, SAXS data showing the concentration dependence of C3Ms, density profiles obtained from SAXS, the effect of ionic strength and pH on C3Ms, CMC determination, additional antimicrobial effects, trypsin breakdown effect, freeze-drying of C3Ms, morphology changes upon cellular treatment, and tables of fit parameter (PDF)

## Abbreviations

AMP: antimicrobial peptides
C3Ms: complex coacervate core micelles
CEAC: critical excess anionic charge
CECC: critical excess cationic charge
CMC: critical micelle concentration
DDA: disc diffusion assay
DLS: dynamic light scattering
HSA: human serum albumin
MIC: minimum inhibitory concentration
PEO-*b*-PMAA: poly(ethylene oxide)-*b*-poly(methacrylic acid)
PDI: polydispersity index
SAXS: small-angle X-ray scattering
SCP: soluble complex particles

## References

[ref1] Tacconelli E.; Carrara E.; Savoldi A.; Harbarth S.; Mendelson M.; Monnet D. L.; Pulcini C.; Kahlmeter G.; Kluytmans J.; Carmeli Y.; Ouellette M.; Outterson K.; Patel J.; Cavaleri M.; Cox E. M.; Houchens C. R.; Grayson M. L.; Hansen P.; Singh N.; Theuretzbacher U.; Magrini N.; Aboderin A. O.; Al-Abri S. S.; Awang Jalil N.; Benzonana N.; Bhattacharya S.; Brink A. J.; Burkert F. R.; Cars O.; Cornaglia G.; Dyar O. J.; Friedrich A. W.; Gales A. C.; Gandra S.; Giske C. G.; Goff D. A.; Goossens H.; Gottlieb T.; Guzman Blanco M.; Hryniewicz W.; Kattula D.; Jinks T.; Kanj S. S.; Kerr L.; Kieny M.-P.; Kim Y. S.; Kozlov R. S.; Labarca J.; Laxminarayan R.; Leder K.; Leibovici L.; Levy-Hara G.; Littman J.; Malhotra-Kumar S.; Manchanda V.; Moja L.; Ndoye B.; Pan A.; Paterson D. L.; Paul M.; Qiu H.; Ramon-Pardo P.; Rodríguez-Baño J.; Sanguinetti M.; Sengupta S.; Sharland M.; Si-Mehand M.; Silver L. L.; Song W.; Steinbakk M.; Thomsen J.; Thwaites G. E.; van der Meer J. W.; Van Kinh N.; Vega S.; Villegas M. V.; Wechsler-Fördös A.; Wertheim H. F. L.; Wesangula E.; Woodford N.; Yilmaz F. O.; Zorzet A. Discovery, Research, and Development of New Antibiotics: The WHO Priority List of Antibiotic-Resistant Bacteria and Tuberculosis. Lancet Infect. Dis. 2018, 18 (3), 318–327. 10.1016/S1473-3099(17)30753-3. 29276051

[ref2] Moretta A.; Scieuzo C.; Petrone A. M.; Salvia R.; Manniello M. D.; Franco A.; Lucchetti D.; Vassallo A.; Vogel H.; Sgambato A.; Falabella P. Antimicrobial Peptides: A New Hope in Biomedical and Pharmaceutical Fields. Front. Cell. Infect. Microbiol. 2021, 11, 668632 10.3389/fcimb.2021.668632. 34195099 PMC8238046

[ref3] Luong H. X.; Thanh T. T.; Tran T. H. Antimicrobial Peptides—Advances in Development of Therapeutic Applications. Life Sci. 2020, 260, 118407 10.1016/j.lfs.2020.118407. 32931796 PMC7486823

[ref4] Mahlapuu M.; Björn C.; Ekblom J. Antimicrobial Peptides as Therapeutic Agents: Opportunities and Challenges. Crit. Rev. Biotechnol. 2020, 40 (7), 978–992. 10.1080/07388551.2020.1796576. 32781848

[ref5] Bahar A. A.; Ren D. Antimicrobial Peptides. Pharmaceuticals 2013, 6 (12), 1543–1575. 10.3390/ph6121543. 24287494 PMC3873676

[ref6] Haney E. F.; Mansour S. C.; Hancock R. E. W. Antimicrobial Peptides: An Introduction. In Antimicrobial Peptides: Methods and Protocols; Hansen P. R., Ed.; Methods in Molecular Biology; Springer: New York, NY, 2017; pp 3–22. 10.1007/978-1-4939-6737-7_1. 28013493

[ref7] Rima M.; Rima M.; Fajloun Z.; Sabatier J.-M.; Bechinger B.; Naas T. Antimicrobial Peptides: A Potent Alternative to Antibiotics. Antibiotics 2021, 10 (9), 1095 10.3390/antibiotics10091095. 34572678 PMC8466391

[ref8] Falagas M. E.; Kasiakou S. K.; Saravolatz L. D. Colistin: The Revival of Polymyxins for the Management of Multidrug-Resistant Gram-Negative Bacterial Infections. Clin. Infect. Dis. 2005, 40 (9), 1333–1341. 10.1086/429323. 15825037

[ref9] Trimble M. J.; Mlynárčik P.; Kolář M.; Hancock R. E. W. Polymyxin: Alternative Mechanisms of Action and Resistance. Cold Spring Harbor Perspect. Med. 2016, 6 (10), a025288 10.1101/cshperspect.a025288. PMC5046685 27503996

[ref10] Dijkmans A. C.; Wilms E. B.; Kamerling I. M. C.; Birkhoff W.; Ortiz-Zacarías N. V.; van Nieuwkoop C.; Verbrugh H. A.; Touw D. J. Colistin: Revival of an Old Polymyxin Antibiotic. Ther. Drug Monit. 2015, 37 (4), 419 10.1097/FTD.0000000000000172. 25549206

[ref11] Liao F.-H.; Wu T.-H.; Yao C.-N.; Kuo S.-C.; Su C.-J.; Jeng U.-S.; Lin S.-Y. A Supramolecular Trap to Increase the Antibacterial Activity of Colistin. Angew. Chem., Int. Ed. 2020, 59 (4), 1430–1434. 10.1002/anie.201912137. PMC7687082 31729106

[ref12] Ordooei Javan A.; Shokouhi S.; Sahraei Z. A Review on Colistin Nephrotoxicity. Eur. J. Clin. Pharmacol. 2015, 71 (7), 801–810. 10.1007/s00228-015-1865-4. 26008213

[ref13] Wallace S. J.; Li J.; Nation R. L.; Boyd B. J. Drug Release from Nanomedicines: Selection of Appropriate Encapsulation and Release Methodology. Drug Delivery Transl. Res. 2012, 2 (4), 284–292. 10.1007/s13346-012-0064-4. PMC3482165 23110256

[ref14] Nation R. L.; Li J. Colistin in the 21st Century. Curr. Opin. Infect. Dis. 2009, 22 (6), 535 10.1097/QCO.0b013e328332e672. 19797945 PMC2869076

[ref15] Dubashynskaya N. V.; Bokatyi A. N.; Gasilova E. R.; Dobrodumov A. V.; Dubrovskii Y. A.; Knyazeva E. S.; Nashchekina Y. A.; Demyanova E. V.; Skorik Y. A. Hyaluronan-Colistin Conjugates: Synthesis, Characterization, and Prospects for Medical Applications. Int. J. Biol. Macromol. 2022, 215, 243–252. 10.1016/j.ijbiomac.2022.06.080. 35724903

[ref16] Liao W.-C.; Wang C.-H.; Sun T.-H.; Su Y.-C.; Chen C.-H.; Chang W.-T.; Chen P.-L.; Shiue Y.-L. The Antimicrobial Effects of Colistin Encapsulated in Chelating Complex Micelles for the Treatment of Multi-Drug-Resistant Gram-Negative Bacteria: A Pharmacokinetic Study. Antibiotics 2023, 12 (5), 836 10.3390/antibiotics12050836. 37237739 PMC10215448

[ref17] Landa G.; Alejo T.; Sauzet T.; Laroche J.; Sebastian V.; Tewes F.; Arruebo M. Colistin-Loaded Aerosolizable Particles for the Treatment of Bacterial Respiratory Infections. Int. J. Pharm. 2023, 635, 122732 10.1016/j.ijpharm.2023.122732. 36803926

[ref18] Vairo C.; Villar Vidal M.; Maria Hernandez R.; Igartua M.; Villullas S. Colistin- and Amikacin-Loaded Lipid-Based Drug Delivery Systems for Resistant Gram-Negative Lung and Wound Bacterial Infections. Int. J. Pharm. 2023, 635, 122739 10.1016/j.ijpharm.2023.122739. 36801363

[ref19] Sans-Serramitjana E.; Jorba M.; Pedraz J. L.; Vinuesa T.; Viñas M. Determination of the Spatiotemporal Dependence of Pseudomonas Aeruginosa Biofilm Viability after Treatment with NLC-Colistin. Int. J. Nanomed. 2017, 12, 4409–4413. 10.2147/IJN.S138763. PMC5476584 28652741

[ref20] Shah S. R.; Henslee A. M.; Spicer P. P.; Yokota S.; Petrichenko S.; Allahabadi S.; Bennett G. N.; Wong M. E.; Kasper F. K.; Mikos A. G. Effects of Antibiotic Physicochemical Properties on Their Release Kinetics from Biodegradable Polymer Microparticles. Pharm. Res. 2014, 31 (12), 3379–3389. 10.1007/s11095-014-1427-y. 24874603 PMC4225168

[ref21] Liu Y.-H.; Kuo S.-C.; Yao B.-Y.; Fang Z.-S.; Lee Y.-T.; Chang Y.-C.; Chen T.-L.; Hu C.-M. J. Colistin Nanoparticle Assembly by Coacervate Complexation with Polyanionic Peptides for Treating Drug-Resistant Gram-Negative Bacteria. Acta Biomater. 2018, 82, 133–142. 10.1016/j.actbio.2018.10.013. 30316023

[ref22] Ezike T. C.; Okpala U. S.; Onoja U. L.; Nwike C. P.; Ezeako E. C.; Okpara O. J.; Okoroafor C. C.; Eze S. C.; Kalu O. L.; Odoh E. C.; Nwadike U. G.; Ogbodo J. O.; Umeh B. U.; Ossai E. C.; Nwanguma B. C. Advances in Drug Delivery Systems, Challenges and Future Directions. Heliyon 2023, 9 (6), e17488 10.1016/j.heliyon.2023.e17488. 37416680 PMC10320272

[ref23] Ban E.; Kim A. Coacervates: Recent Developments as Nanostructure Delivery Platforms for Therapeutic Biomolecules. Int. J. Pharm. 2022, 624, 122058 10.1016/j.ijpharm.2022.122058. 35905931

[ref24] Blocher W. C.; Perry S. L. Complex Coacervate-Based Materials for Biomedicine. Wiley Interdiscip. Rev.: Nanomed. Nanobiotechnol. 2017, 9 (4), e1442 10.1002/wnan.1442. 27813275

[ref25] Borro B. C.; Malmsten M. Complexation between Antimicrobial Peptides and Polyelectrolytes. Adv. Colloid Interface Sci. 2019, 270, 251–260. 10.1016/j.cis.2019.07.001. 31301601

[ref26] Jenssen H.; Aspmo S. I. Serum Stability of Peptides. In Peptide-Based Drug Design; Otvos L., Ed.; Methods in Molecular Biology; Humana Press: Totowa, NJ, 2008; pp 177–186. 10.1007/978-1-59745-419-3_10. 18726574

[ref27] Voets I. K.; de Keizer A.; Cohen Stuart M. A. Complex Coacervate Core Micelles. Adv. Colloid Interface Sci. 2009, 147–148, 300–318. 10.1016/j.cis.2008.09.012. 19038373

[ref28] Magana J. R.; Sproncken C. C. M.; Voets I. K. On Complex Coacervate Core Micelles: Structure-Function Perspectives. Polymers 2020, 12 (9), 1953 10.3390/polym12091953. 32872312 PMC7565781

[ref29] Marciel A. B.; Srivastava S.; Ting J. M.; Tirrell M. V. SAXS Methods for Investigating Macromolecular and Self-Assembled Polyelectrolyte Complexes. In Methods in Enzymology; Keating C. D., Ed.; Liquid-Liquid Phase Coexistence and Membraneless Organelles; Academic Press, 2021; Chapter 8, Vol. 646; pp 223–259. 10.1016/bs.mie.2020.09.013. 33453927

[ref30] Rumyantsev A. M.; Jackson N. E.; de Pablo J. J. Polyelectrolyte Complex Coacervates: Recent Developments and New Frontiers. Annu. Rev. Condens. Matter Phys. 2021, 12 (1), 155–176. 10.1146/annurev-conmatphys-042020-113457.

[ref31] Uebbing L.; Ziller A.; Siewert C.; Schroer M. A.; Blanchet C. E.; Svergun D. I.; Ramishetti S.; Peer D.; Sahin U.; Haas H.; Langguth P. Investigation of pH-Responsiveness inside Lipid Nanoparticles for Parenteral mRNA Application Using Small-Angle X-Ray Scattering. Langmuir 2020, 36 (44), 13331–13341. 10.1021/acs.langmuir.0c02446. 33108188

[ref32] Grabbe S.; Haas H.; Diken M.; Kranz L. M.; Langguth P.; Sahin U. Translating Nanoparticulate-Personalized Cancer Vaccines into Clinical Applications: Case Study with RNA-Lipoplexes for the Treatment of Melanoma. Nanomedicine 2016, 11 (20), 2723–2734. 10.2217/nnm-2016-0275. 27700619

[ref33] Nogueira S. S.; Schlegel A.; Maxeiner K.; Weber B.; Barz M.; Schroer M. A.; Blanchet C. E.; Svergun D. I.; Ramishetti S.; Peer D.; Langguth P.; Sahin U.; Haas H. Polysarcosine-Functionalized Lipid Nanoparticles for Therapeutic mRNA Delivery. ACS Appl. Nano Mater. 2020, 3 (11), 10634–10645. 10.1021/acsanm.0c01834.

[ref34] Bos I.; Timmerman M.; Sprakel J. FRET-Based Determination of the Exchange Dynamics of Complex Coacervate Core Micelles. Macromolecules 2021, 54 (1), 398–411. 10.1021/acs.macromol.0c02387. 33456072 PMC7808214

[ref35] Amann M.; Diget J. S.; Lyngsø J.; Pedersen J. S.; Narayanan T.; Lund R. Kinetic Pathways for Polyelectrolyte Coacervate Micelle Formation Revealed by Time-Resolved Synchrotron SAXS. Macromolecules 2019, 52 (21), 8227–8237. 10.1021/acs.macromol.9b01072.

[ref36] Voets I. K.; Moll P. M.; Aqil A.; Jérôme C.; Detrembleur C.; de Waard P.; de Keizer A.; Stuart M. A. C. Temperature Responsive Complex Coacervate Core Micelles With a PEO and PNIPAAm Corona. J. Phys. Chem. B 2008, 112 (35), 10833–10840. 10.1021/jp8014832. 18698810

[ref37] Sproncken C. C. M.; Surís-Valls R.; Cingil H. E.; Detrembleur C.; Voets I. K. Complex Coacervate Core Micelles Containing Poly(Vinyl Alcohol) Inhibit Ice Recrystallization. Macromol. Rapid Commun. 2018, 39 (17), e1700814 10.1002/marc.201700814. 29635766

[ref38] Voets I. K.; van der Burgh S.; Farago B.; Fokkink R.; Kovacevic D.; Hellweg T.; de Keizer A.; Cohen Stuart M. A. Electrostatically Driven Coassembly of a Diblock Copolymer and an Oppositely Charged Homopolymer in Aqueous Solutions. Macromolecules 2007, 40 (23), 8476–8482. 10.1021/ma071356z.

[ref39] Priftis D.; Leon L.; Song Z.; Perry S. L.; Margossian K. O.; Tropnikova A.; Cheng J.; Tirrell M. Self-Assembly of α-Helical Polypeptides Driven by Complex Coacervation. Angew. Chem. 2015, 127 (38), 11280–11284. 10.1002/ange.201504861. 26352023

[ref40] Choi J.-W.; Heo T.-Y.; Choi H.; Choi S.-H.; Won J.-I. Co-Assembly Behavior of Oppositely Charged Thermoresponsive Elastin-like Polypeptide Block Copolymers. J. Appl. Polym. Sci. 2022, 139 (38), e52906 10.1002/app.52906.

[ref41] Lim C.; Roeck Won W.; Moon J.; Sim T.; Shin Y.; Chang Kim J.; Seong Lee E.; Seok Youn Y.; Taek Oh K. Co-Delivery of d -(KLAKLAK) 2 Peptide and Doxorubicin Using a pH-Sensitive Nanocarrier for Synergistic Anticancer Treatment. J. Mater. Chem. B 2019, 7 (27), 4299–4308. 10.1039/C9TB00741E.

[ref42] Lindhoud S.; Voorhaar L.; de Vries R.; Schweins R.; Cohen Stuart M. A.; Norde W. Salt-Induced Disintegration of Lysozyme-Containing Polyelectrolyte Complex Micelles. Langmuir 2009, 25 (19), 11425–11430. 10.1021/la901591p. 19691276

[ref43] Obermeyer A. C.; Mills C.; Dong X.-H.; Flores R.; Olsen B. Complex Coacervation of Supercharged Proteins with Polyelectrolytes. Soft Matter 2016, 12 (15), 3570–3581. 10.1039/C6SM00002A. 26965053

[ref44] Xu A. Y.; Kizilay E.; Madro S. P.; Vadenais J. Z.; McDonald K. W.; Dubin P. L. Dilution Induced Coacervation in Polyelectrolyte-Micelle and Polyelectrolyte-Protein Systems. Soft Matter 2018, 14 (12), 2391–2399. 10.1039/C7SM02293J. 29503995

[ref45] Marras A. E.; Ting J. M.; Stevens K. C.; Tirrell M. V. Advances in the Structural Design of Polyelectrolyte Complex Micelles. J. Phys. Chem. B 2021, 125 (26), 7076–7089. 10.1021/acs.jpcb.1c01258. 34160221 PMC9282648

[ref46] Abbas M.; Lipiński W.; Wang J.; Spruijt E. Peptide-Based Coacervates as Biomimetic Protocells. Chem. Soc. Rev. 2021, 50 (6), 3690–3705. 10.1039/D0CS00307G. 33616129

[ref47] Brancaccio D.; Pizzo E.; Cafaro V.; Notomista E.; De Lise F.; Bosso A.; Gaglione R.; Merlino F.; Novellino E.; Ungaro F.; Grieco P.; Malanga M.; Quaglia F.; Miro A.; Carotenuto A. Antimicrobial Peptide Temporin-L Complexed with Anionic Cyclodextrins Results in a Potent and Safe Agent against Sessile Bacteria. Int. J. Pharm. 2020, 584, 119437 10.1016/j.ijpharm.2020.119437. 32447024

[ref48] Insua I.; Majok S.; Peacock A. F. A.; Krachler A. M.; Fernandez-Trillo F. Preparation and Antimicrobial Evaluation of Polyion Complex (PIC) Nanoparticles Loaded with Polymyxin B. Eur. Polym. J. 2017, 87, 478–486. 10.1016/j.eurpolymj.2016.08.023. 28280277 PMC5327956

[ref49] Răileanu M.; Lonetti B.; Serpentini C.-L.; Goudounèche D.; Gibot L.; Bacalum M. Encapsulation of a Cationic Antimicrobial Peptide into Self-Assembled Polyion Complex Nano-Objects Enhances Its Antitumor Properties. J. Mol. Struct. 2022, 1249, 131482 10.1016/j.molstruc.2021.131482.

[ref50] Wang C.; Feng S.; Qie J.; Wei X.; Yan H.; Liu K. Polyion Complexes of a Cationic Antimicrobial Peptide as a Potential Systemically Administered Antibiotic. Int. J. Pharm. 2019, 554, 284–291. 10.1016/j.ijpharm.2018.11.029. 30439489

[ref51] Niece K. L.; Vaughan A. D.; Devore D. I. Graft Copolymer Polyelectrolyte Complexes for Delivery of Cationic Antimicrobial Peptides. J. Biomed. Mater. Res., Part A 2013, 101 (9), 2548–2558. 10.1002/jbm.a.34555. 23364909

[ref52] Tully M. D.; Kieffer J.; Brennich M. E.; Cohen Aberdam R.; Florial J. B.; Hutin S.; Oscarsson M.; Beteva A.; Popov A.; Moussaoui D.; Theveneau P.; Papp G.; Gigmes J.; Cipriani F.; McCarthy A.; Zubieta C.; Mueller-Dieckmann C.; Leonard G.; Pernot P. BioSAXS at European Synchrotron Radiation Facility—Extremely Brilliant Source: BM29 with an Upgraded Source, Detector, Robot, Sample Environment, Data Collection and Analysis Software. J. Synchrotron Radiat. 2023, 30 (1), 258–266. 10.1107/S1600577522011286. 36601945 PMC9814054

[ref53] Berndt I.; Pedersen J. S.; Lindner P.; Richtering W. Influence of Shell Thickness and Cross-Link Density on the Structure of Temperature-Sensitive Poly-N-Isopropylacrylamide–Poly-N-Isopropylmethacrylamide Core–Shell Microgels Investigated by Small-Angle Neutron Scattering. Langmuir 2006, 22 (1), 459–468. 10.1021/la052463u. 16378460

[ref54] Berndt I.; Pedersen J. S.; Richtering W. Temperature-Sensitive Core–Shell Microgel Particles with Dense Shell. Angew. Chem. 2006, 118 (11), 1769–1773. 10.1002/ange.200503888. 16470901

[ref55] Pedersen J. S.; Svaneborg C. Scattering from Block Copolymer Micelles. Curr. Opin. Colloid Interface Sci. 2002, 7 (3), 158–166. 10.1016/S1359-0294(02)00044-4.

[ref56] Lund R.; Willner L.; Richter D. Kinetics of Block Copolymer Micelles Studied by Small-Angle Scattering Methods. In Controlled Polymerization and Polymeric Structures: Flow Microreactor Polymerization, Micelles Kinetics, Polypeptide Ordering, Light Emitting Nanostructures; Abe A.; Lee K.-S.; Leibler L.; Kobayashi S., Eds.; Advances in Polymer Science; Springer International Publishing: Cham, 2013; pp 51–158. 10.1007/12_2012_204.

[ref57] Pedersen J. S. Structure Factors Effects in Small-Angle Scattering from Block Copolymer Micelles and Star Polymers. J. Chem. Phys. 2001, 114 (6), 2839–2846. 10.1063/1.1339221.

[ref58] Lund R.; Willner L.; Stellbrink J.; Radulescu A.; Richter D. Role of Interfacial Tension for the Structure of PEP–PEO Polymeric Micelles. A Combined SANS and Pendant Drop Tensiometry Investigation. Macromolecules 2004, 37 (26), 9984–9993. 10.1021/ma035633n.

[ref59] Fang Y. N.; Rumyantsev A. M.; Neitzel A. E.; Liang H.; Heller W. T.; Nealey P. F.; Tirrell M. V.; de Pablo J. J. Scattering Evidence of Positional Charge Correlations in Polyelectrolyte Complexes. Proc. Natl. Acad. Sci. U.S.A. 2023, 120 (32), e2302151120 10.1073/pnas.2302151120. 37523553 PMC10410704

[ref60] Debye P. Molecular-Weight Determination by Light Scattering. J. Phys. Chem. A 1947, 51 (1), 18–32. 10.1021/j150451a002. 20286386

[ref61] Hofs B.; Brzozowska A.; de Keizer A.; Norde W.; Cohen Stuart M. A. Reduction of Protein Adsorption to a Solid Surface by a Coating Composed of Polymeric Micelles with a Glass-like Core. J. Colloid Interface Sci. 2008, 325 (2), 309–315. 10.1016/j.jcis.2008.06.006. 18589433

[ref62] Brzozowska A. M.; Hofs B.; de Keizer A.; Fokkink R.; Cohen Stuart M. A.; Norde W. Reduction of Protein Adsorption on Silica and Polystyrene Surfaces Due to Coating with Complex Coacervate Core Micelles. Colloids Surf., A. 2009, 347 (1), 146–155. 10.1016/j.colsurfa.2009.03.036.

[ref63] Makowski M.; Silva Í. C.; Pais do Amaral C.; Gonçalves S.; Santos N. C. Advances in Lipid and Metal Nanoparticles for Antimicrobial Peptide Delivery. Pharmaceutics 2019, 11 (11), 588 10.3390/pharmaceutics11110588. 31717337 PMC6920925

[ref64] Eftekhari A.; Arjmand A.; Asheghvatan A.; Švajdlenková H.; Šauša O.; Abiyev H.; Ahmadian E.; Smutok O.; Khalilov R.; Kavetskyy T.; Cucchiarini M. The Potential Application of Magnetic Nanoparticles for Liver Fibrosis Theranostics. Front. Chem. 2021, 9, 674786 10.3389/fchem.2021.674786. 34055744 PMC8161198

[ref65] Weldick P. J.; Wang A.; F. Halbus A.; N. Paunov V. Emerging Nanotechnologies for Targeting Antimicrobial Resistance. Nanoscale 2022, 14 (11), 4018–4041. 10.1039/D1NR08157H. 35234774

[ref66] Rajchakit U.; Sarojini V. Recent Developments in Antimicrobial-Peptide-Conjugated Gold Nanoparticles. Bioconjugate Chem. 2017, 28 (11), 2673–2686. 10.1021/acs.bioconjchem.7b00368. 28892365

[ref67] Da Vela S.; Svergun D. I. Methods, Development and Applications of Small-Angle X-Ray Scattering to Characterize Biological Macromolecules in Solution. Curr. Res. Struct. Biol. 2020, 2, 164–170. 10.1016/j.crstbi.2020.08.004. 34235476 PMC8244429

[ref68] Nolles A.; Hooiveld E.; Westphal A. H.; van Berkel W. J. H.; Kleijn J. M.; Borst J. W. FRET Reveals the Formation and Exchange Dynamics of Protein-Containing Complex Coacervate Core Micelles. Langmuir 2018, 34 (40), 12083–12092. 10.1021/acs.langmuir.8b01272. 30212214 PMC6209312

[ref69] Tian B.; Liu S.; Lu W.; Jin L.; Li Q.; Shi Y.; Li C.; Wang Z.; Du Y. Construction of pH-Responsive and up-Conversion Luminescent NaYF4:Yb3+/Er3+@SiO2@PMAA Nanocomposite for Colon Targeted Drug Delivery. Sci. Rep. 2016, 6 (1), 21335 10.1038/srep21335. 26891778 PMC4759527

[ref70] Bernett M. K.; Zisman W. A. Relation of Wettability by Aqueous Solutions to the Surface Constitution of Low-Energy Solids. J. Phys. Chem. A 1959, 63 (8), 1241–1246. 10.1021/j150578a006.

[ref71] Balouiri M.; Sadiki M.; Ibnsouda S. K. Methods for in Vitro Evaluating Antimicrobial Activity: A Review. J. Pharm. Anal. 2016, 6 (2), 71–79. 10.1016/j.jpha.2015.11.005. 29403965 PMC5762448

[ref72] Kourmouli A.; Valenti M.; van Rijn E.; Beaumont H. J. E.; Kalantzi O.-I.; Schmidt-Ott A.; Biskos G. Can Disc Diffusion Susceptibility Tests Assess the Antimicrobial Activity of Engineered Nanoparticles?. J. Nanopart. Res. 2018, 20 (3), 62 10.1007/s11051-018-4152-3. 29527123 PMC5834581

[ref73] Bardet L.; Okdah L.; Le Page S.; Baron S. A.; Rolain J.-M. Comparative Evaluation of the UMIC Colistine Kit to Assess MIC of Colistin of Gram-Negative Rods. BMC Microbiol. 2019, 19 (1), 60 10.1186/s12866-019-1424-8. 30885126 PMC6421643

[ref74] Matuschek E.; Åhman J.; Webster C.; Kahlmeter G. Antimicrobial Susceptibility Testing of Colistin—Evaluation of Seven Commercial MIC Products against Standard Broth Microdilution for *Escherichia coli*, Klebsiella Pneumoniae, Pseudomonas Aeruginosa, and Acinetobacter Spp. Clin. Microbiol. Infect. 2018, 24 (8), 865–870. 10.1016/j.cmi.2017.11.020. 29221995

[ref75] Matzneller P.; Gobin P.; Lackner E.; Zeitlinger M. Feasibility of Microdialysis for Determination of Protein Binding and Target Site Pharmacokinetics of Colistin in Vivo. J. Clin. Pharmacol. 2015, 55 (4), 431–437. 10.1002/jcph.419. 25359520

[ref76] Shen S. I.; Jasti B.; Li X. Design of Controlled Release Drug Delivery Systems, McGraw-Hill Chemical Engineering; McGraw-Hill: New York, 2006.

[ref77] Voets I. K. Electrostatically Driven Assembly of Polyelectrolytes. In Fluorescence Studies of Polymer Containing Systems; Procházka K., Ed.; Springer Series on Fluorescence; Springer International Publishing: Cham, 2016; pp 65–89. 10.1007/978-3-319-26788-3_3.

[ref78] El-Andaloussi S.; Järver P.; Johansson H. J.; Langel Ü. Cargo-Dependent Cytotoxicity and Delivery Efficacy of Cell-Penetrating Peptides: A Comparative Study. Biochem. J. 2007, 407 (2), 285–292. 10.1042/BJ20070507. 17627607 PMC2049024

[ref79] Peired A. J.; Sisti A.; Romagnani P. Mesenchymal Stem Cell-Based Therapy for Kidney Disease: A Review of Clinical Evidence. Stem Cells Int. 2016, 2016, 4798639 10.1155/2016/4798639. 27721835 PMC5046016

[ref80] Eaton D. C. Frontiers in Renal and Epithelial Physiology – Grand Challenges. Front. Physiol. 2012, 3, 2 10.3389/fphys.2012.00002. 22275903 PMC3258550

[ref81] McGinn S.; Poronnik P.; Gallery E. D. M.; Pollock C. A. A Method for the Isolation of Glomerular and Tubulointerstitial Endothelial Cells and a Comparison of Characteristics with the Human Umbilical Vein Endothelial Cell Model. Nephrology 2004, 9 (4), 229–237. 10.1111/j.1440-1797.2004.00254.x. 15363055

[ref82] Chan F. K.-M.; Moriwaki K.; De Rosa M. J. Detection of Necrosis by Release of Lactate Dehydrogenase Activity. In Immune Homeostasis: Methods and Protocols; Snow A. L.; Lenardo M. J., Eds.; Methods in Molecular Biology; Humana Press: Totowa, NJ, 2013; pp 65–70. 10.1007/978-1-62703-290-2_7. PMC3763497 23397389

[ref83] Porter A. G.; Jänicke R. U. Emerging Roles of Caspase-3 in Apoptosis. Cell Death Differ. 1999, 6 (2), 99–104. 10.1038/sj.cdd.4400476. 10200555

[ref84] Zhivotovsky B. Apoptosis, Necrosis and Between. Cell Cycle 2004, 3 (1), 63–65. 10.4161/cc.3.1.606. 14657668

[ref85] Torres-Lugo M.; García M.; Record R.; Peppas N. A. Physicochemical Behavior and Cytotoxic Effects of p(Methacrylic Acid–g-Ethylene Glycol) Nanospheres for Oral Delivery of Proteins. J. Controlled Release 2002, 80 (1), 197–205. 10.1016/S0168-3659(02)00027-5. 11943398

[ref86] Miatmoko A. Physical Characterization and Biodistribution of Cisplatin Loaded in Surfactant Modified-Hybrid Nanoparticles Using Polyethylene Oxide-b-Polymethacrylic Acid. Adv. Pharm. Bull. 2020, 11 (4), 765–771. 10.34172/apb.2021.086. 34888224 PMC8642799

