# Supplementary material for: Crafting Stable Antibiotic Nanoparticles via Complex Coacervation of Colistin with Block Copolymers
Source: Biomacromolecules. 2024 Jun 17;25(7):4267–80. doi: 10.1021/acs.biomac.4c00337 (PMC11238337; doi:10.1021/acs.biomac.4c00337)
Supplement: Supplementary file 1 — bm4c00337_si_001.pdf [file bm4c00337_si_001.pdf]

Supporting Information for

Crafting Stable Antibiotic Nanoparticles via Complex Coacervation of Colistin with Block Copolymers

Thomas D. Vogelaar[1], Anne E. Agger[2], Janne E. Reseland[2], Dirk Linke[3], Håvard Jenssen[4], and Reidar Lund[1,5]*

[1]Department of Chemistry, University of Oslo, P.O. Box 1033 Blindern, NO-0315 Oslo, Norway

[2]Department of Biomaterials, Institute of Clinical Dentistry, University of Oslo, P.O. Box 1109 Blindern, NO-0317 Oslo, Norway

[3]Department of Biosciences, University of Oslo, P.O. Box 1066 Blindern, NO-0316 University of Oslo, Oslo, Norway

[4]Department of Science and Environment, Roskilde University, 4000 Roskilde, Denmark

[5]Hylleraas Centre for Quantum Molecular Sciences, University of Oslo, NO-0315 Oslo, Norway

Table of Contents

1. SAXS modeling fuzzy-surface complex coacervate model in detail
    1.1. Mass balance contributions
    1.2. Scattering contributions
    1.3. Complex coacervate scattering
    1.4. Free component scattering
    1.5. Internal structure scattering
    1.6. Combining all scattering components
2. Results and Discussion
    2.1. DLS measurements at different charge ratios (f_+) of complex coacervates made from different block lengths of PEO-b-PMAA
    2.2. SAXS concentration independence
    2.3. Varying ionic strength and pH for P1-colistin-C3Ms at $f_+ = 0.50$
    2.4. CMC determination
    2.5. Antimicrobial properties
    2.6. Trypsin breakdown
    2.7. Freeze-drying C3Ms
    2.8. Cell morphology changes due to toxicity
    2.9. SAXS model fits
3. References

1. SAXS modeling, fuzzy-surface complex coacervate model in detail

1.1. Mass balance contributions

Based on the zeta potential measurements, we assumed that there is stoichiometric charge-matching inside the core, reducing the number of parameters and fixing the f_{mix0} parameter, which was defined as the molar fraction of polymer in the polyelectrolyte core. To explain the model, we defined several parameters for the mass balances of SCP and C3M structures. With a sound mass balance, it is possible to get more information from the scattering curves since there are many characteristics from the scattering contributions from complex coacervates, free peptide and polymer, clusters, internal structures, and blob scattering among the whole Q-range. First, the volumes of the core (V_{cp}) and the fuzzy-interface PEO-shell (V_{sp}), the total volume (V_{tot}), and the volume fraction (ϕ) are calculated (Eq. S1-4).

$$V_{cp} = \frac{\left((1-f_{mix0})\cdot\frac{M_{Col0}}{d_{Col}}+f_{mix0}\cdot\frac{M_{PMAA}}{d_{PMAA}}\right)}{N_A} \qquad \text{(S1)}$$

In which V_{cp} is the mean core block volume from one average complex of colistin and the PMAA block in Å^3, f_{mix0} is the molar fraction of polymer inside the core, M_{Col0} the molecular weight of colistin excluding sulfate salt, d_{Col} the density of colistin, M_{PMAA}, the molecular weight of the anionic block of the polymer, d_{PMAA} the density of the anionic block of the polymer and N_A Avogadro's number.

$$V_{sp} = \frac{f_{mix0}\cdot\frac{M_{PEO}}{d_{PEO}}}{N_A} \qquad \text{(S2)}$$

In which V_{sp} is the effective volume of the PEO block of one chain in Å^3, M_{PEO} is the molecular weight of the neutral PEO block of the polymer, and d_{PEO} is the density of the PEO block of the polymer.

$$V_{tot} = V_{cp} + V_{sp} \qquad \text{(S3)}$$

V_{tot} is the total effective volume from both contributions from one average chain in the complex coacervate.

$$\varphi = \frac{c_{Col}+c_{Poly}}{d_{average}} \qquad \text{(S4)}$$

In which ϕ is the volume fraction, c_{Col}, the concentration of colistin and c_{Poly}, the concentration of polymer and $d_{average}$, the average specific density of the components making up the system. When going off the charge-matching, we assume that the internal charge ratio remains constant while the external charge ratio will change in the aqueous environment around the complex coacervates, causing a mismatch in molar fractions inside and outside the core. To grasp the effect of these changes the molar fraction of polymer in the aqueous phase is calculated (f_{mix}) (Eq. S5).

$$f_{mix} = \frac{\frac{\frac{c_{Poly}}{M_{PMAA}+M_{PEO}} - \frac{f_{mix0}}{1-f_{mix0}} \cdot \frac{(1-f_{Col}) \cdot c_{Col}}{M_{Col}}}{\frac{f_{Col} \cdot c_{Col}}{M_{Col}}}}{1+\frac{\frac{c_{Poly}}{M_{PMAA}+M_{PEO}} - \frac{f_{mix0}}{1-f_{mix0}} \cdot \frac{(1-f_{Col}) \cdot c_{Col}}{M_{Col}}}{\frac{f_{Col} \cdot c_{Col}}{M_{Col}}}} \qquad \text{(S5)}$$

In which f_{mix} is the molar fraction of polymer in the aqueous phase surrounding the complex coacervates, f_{Col} is the free fraction of colistin, which is the first fitting parameter in our model. M_{Col} is the molecular weight of colistin, including the sulfate salt. Based on the f_{mix} fraction, the free fraction of polymer (f_{Poly}) and the fraction of complex coacervates (f_{Coa}) can be calculated (Eq. S6, S7).

$$f_{Poly} = \frac{f_{mix} \cdot (M_{PMAA}+M_{PEO}) + f_{Col} \cdot \frac{c_{Col}}{M_{Col}}}{c_{Poly} \cdot (1-f_{mix})} \qquad \text{(S6)}$$

$$f_{Coa} = \frac{(1-f_{Poly}) \cdot c_{Poly} + (1-f_{Col}) \cdot c_{Col}}{c_{Col} + c_{Poly}} \qquad \text{(S7)}$$

f_{Poly}, the free fraction of polymer not incorporated into micelles, and f_{Coa}, the fraction of complex coacervates inside the whole system, were calculated from terms already explained previously. The C3Ms are treated as having an inhomogeneous internal structure with a fuzzy surface. Therefore, the density distribution is described by a profile with graded interfaces[1]. Based on these fuzzy surfaces, the radii of the complex coacervates are explained as follows (Eq. S8-10).

$$R_{core} = |R_{in}| + |\sigma_{in}| \qquad \text{(S8)}$$

$$R_{out} = |R_{in}| + |\sigma_{in}| + |\sigma_{out}| \qquad \text{(S9)}$$

$$R_{tot} = |R_{out}| + |\sigma_{out}| \qquad \text{(S10)}$$

R_{core} (the radius of the core), R_{out} (the outer radius of the coacervate), and R_{tot} (the total complex coacervate radius) are calculated with these formulas. In these, the σ_{in} and σ_{out} are Gaussian core-shell interface smearing and Gaussian shell-solvent interface smearing, respectively, while R_{in} is the core radius. These three parameters are fitting parameters.

1.2. Scattering contributions

For the calculations of the scattering contributions, the scattering length densities (SLDs) need to be calculated based on the mass balances. First, the SLDs of the separate components were calculated using the Thomson radius. For polymer blocks, the respective monomers were taken to calculate the SLDs (Eq. S11).

$$\rho = \frac{Z \cdot N_A \cdot d \cdot r_e}{M_w} \qquad \text{(S11)}$$

The SLD (ρ) can be calculated from the number of electrons (Z), the density (d), the classical Thomson radius (r_e), and the molecular weight (M_w). With those SLDs calculated for colistin, PMAA, and PEO, averaged SLDs can be calculated, in this case (ρ_{Core}), after which the average scattering length difference ($\Delta\rho_{average}$) was calculated (Eq. S12, S13).

$$\rho_{Core} = \frac{(1-f_{mix0})\cdot \rho_{Col}\cdot \frac{M_{Col0}}{d_{Col}} + f_{mix0}\cdot \rho_{PMAA}\cdot \frac{M_{0PMAA}}{d_{PMAA}}}{(1-f_{mix0})\cdot \frac{M_{Col0}}{d_{Col}} + f_{mix0}\cdot \frac{M_{0PMAA}}{d_{PMAA}}} \quad \text{(S12)}$$

$$\Delta\rho_{average} = \frac{V_{cp}\cdot \rho_{Core} + V_{sp}\cdot \rho_{PEO}}{V_{cp} + V_{sp}} - \rho_{Solvent} \quad \text{(S13)}$$

In which the M_{0PMAA} is the monomer mass of PMAA. The SLDs were all fixed parameters, including the solvent. We can divide the scattering model into three scattering contributions: complex coacervate scattering, free component scattering, and an internal structure factor from the internal anionic spacing inside the polyelectrolyte core. The three components are combined into one formula for the scattering intensity.

1.3. Complex coacervate scattering

The complex coacervate scattering is based on the scattering formula of centrosymmetric particles, which can generally be written as follows (Eq. S14).

$$I(Q) = n \cdot \Delta\rho^2 \cdot {V_p}^2 \cdot P(Q) \cdot S(Q) \quad \text{(S14)}$$

In which n is the number density of scatterers, the $\Delta\rho$ the SLD contrast, the V_p is the volume of the particle, $P(Q)$ is the form factor of the particle, and $S(Q)$ is the structure factor of the particle. In the case of complex coacervate scattering, n can be written as follows (Eq. S15).

$$n = \frac{\varphi}{V_{Coa}} \quad \text{(S15)}$$

V_{coa} is the volume of one coacervate complex (several chains), excluding the water incorporated. The scattering amplitude of a fuzzy sphere (A_{core}), which is the form factor if it is squared, can be described as follows[2,3] and integrated into the formula for centrosymmetric particles, excluding the structure factor and volume fraction, as these are added later in the total equation (Eq. S16-20).

$$F_0 = \left(\frac{R}{\sigma^2} + \frac{1}{\sigma}\right) \cdot \frac{\cos(Q\cdot(R+\sigma))}{Q^4} - \frac{3\cdot \sin\,(Q\cdot(R+\sigma))}{Q^5\cdot\sigma^2} + \left(\frac{R}{\sigma^2} - \frac{1}{\sigma}\right) \cdot \frac{\cos\,(Q(R-\sigma))}{Q^4} \quad \text{(S16)}$$

$$F_1 = \frac{-3\cdot \sin\big(Q\cdot(R+\sigma)\big) + 6\cdot \sin(Q * R)}{Q^5\cdot\sigma^2} - \frac{2\cdot R\cdot \cos\,(Q\cdot R)}{Q^4\cdot\sigma^2} \quad \text{(S17)}$$

$$V_n = \frac{R^3}{3} + \frac{R \cdot \sigma^2}{6} \quad \text{(S18)}$$

$$A_{core}(Q) = \frac{F_0 + F_1}{V_n} \quad \text{(S19)}$$

$$I_{coa}(Q) = \frac{\Delta\rho_{average}{}^2 \cdot P^2 \cdot V_{tot}{}^2 \cdot A_{core}(Q)^2}{V_{Coa}} \quad \text{(S20)}$$

In which the I_{Coa}(Q) is the scattering contribution from the complex coacervates, P is the aggregation number, the number of molecules that make up one micelle, which is a fitting parameter, and $A_{core}(Q)$ is the scattering amplitude of the fuzzy core. Other parameters were mentioned previously. This scattering equation is not yet complete. For the complex coacervates, if there is any cluster formation, a structure factor is needed to explain the scattering. The structure factor for clusters, $S(Q)_{cluster}$, is described by the following equations[4] (Eq. S21-26).

$$p = N_{clu} - floor(N_{clu}) \quad \text{(S21)}$$

$$D = 2 \cdot R_{tot} \cdot f_{dist} \quad \text{(S22)}$$

$$S_x = \frac{\sin(Q \cdot D)}{Q \cdot D} \quad \text{(S23)}$$

$$S_n = \frac{2}{1 - S_x} - 1 - \frac{2\left(1 - S_x{}^{floor(N_{clu})}\right) \cdot S_x}{floor(N_{clu}) \cdot (1 - S_x)^2} \quad \text{(S24)}$$

$$S_{n1} = \frac{2}{1 - S_x} - 1 - \frac{2\left(1 - S_x{}^{(floor(N_{clu})+1)}\right) \cdot S_x}{(floor(N_{clu})+1) \cdot (1 - S_x)^2} \quad \text{(S25)}$$

$$S(Q)_{cluster} = (1 - p) \cdot S_n + p \cdot S_{n1} \quad \text{(S26)}$$

In which N_{clu} is the number of clusters and f_{dist} is the distance correlation of the clusters. If necessary for fitting, these two parameters were fitting parameters. Apart from the structure factor, there is also a scattering contribution from the blob scattering from the polyelectrolytes in the core. The blob scattering is described in the following equation (Eq. S27).

$$blob\,(Q) = V_{tot}{}^2 \cdot P \cdot \Delta\rho_{average}{}^2 \cdot \frac{|f_{blob}|}{(1 + Q^2 \cdot \xi^2)} \quad \text{(S27)}$$

In which the f_{blob} is the fraction of blob scattering, and ξ is the correlation length of the blobs. Both these parameters are fitting parameters in the model.

1.4. Free component scattering

Outside the complex coacervate surroundings there is a free component scattering fraction for either polymer or colistin and potentially even added components like serum proteins or enzymes. Again, the equation for centrosymmetric particles could be used. In this case, we use a form factor instead of a scattering amplitude. In the free component scattering, the Debye

form factor $P(Q)$ for polymers and polyelectrolytes describes the scattering[5] (Eq. S28). In the case of the presence of globular proteins, an ellipsoid form factor is used[6,7] (Eq. S29-S31).

$$P(Q)_{Debye} = \frac{2(e^{-Q^2 \cdot R_g{}^2} - 1 + Q^2 \cdot R_g{}^2)}{(Q^2 \cdot R_g{}^2)^2} \qquad \text{(S28)}$$

$$r = R(sin^2\alpha + \varepsilon^2 cos^2\alpha)^{1/2} \qquad \text{(S29)}$$

$$A_{sph}(x) = 3[\sin(x) - xcos(x)]/x^3 \qquad \text{(S30)}$$

$$P(Q)_{Ellipsoid} = \int_0^{\pi} A_{sph}(Qr)^2 \sin(\alpha) d\alpha \qquad \text{(S31)}$$

In which the R_g is the radius of gyration of the polymer/polyelectrolyte/globular protein. The ellipsoid of revolution has two minor core radii of R and major axis εR, respectively. For the free chains of polymer and colistin, the Debye form factor was taken and implemented in their scattering equation $P(Q)_{Poly}$ and $P(Q)_{Col}$. Additionally, $P(Q)_{Ellipsoid}$ was implemented for the scattering of additional scattering from enzymes and HSA. The scattering contributions can be summarized as the following equations, assuming $S(Q)$ = 1 and excluding the volume fraction from this equation (Eq. S32-35).

$$I(Q)_{Poly,free} = \frac{(\frac{M_{PMAA}}{d_{PMAA}} + \frac{M_{PEO}}{d_{PEO}}) \cdot \Delta\rho_{Poly}{}^2 \cdot P(Q)_{Debye,\ Poly}}{N_A} \qquad \text{(S32)}$$

$$I(Q)_{Col,free} = \frac{(\frac{M_{Col0}}{d_{Col}}) \cdot \Delta\rho_{Col}{}^2 \cdot P(Q)_{Debye,\ Col}}{N_A} \qquad \text{(S33)}$$

$$I(Q)_{Enzymes} = \frac{(\frac{M_{Enzymes}}{d_{Enzymes}}) \cdot \Delta\rho_{Enzymes}{}^2 \cdot P(Q)_{Ellipsoid}}{N_A} \qquad \text{(S34)}$$

$$I(Q)_{HSA} = \frac{(\frac{M_{HSA}}{d_{HSA}}) \cdot \Delta\rho_{HSA}{}^2 \cdot P(Q)_{Ellipsoid}}{N_A} \qquad \text{(S35)}$$

The scattering from the free chains of polymer, colistin, and other added components are described here, including each a form factor specific to the chain itself. For the added components, to obtain the SLD and R_g for the form factor, the contributions were first fitted by the Debye model using the least-squares fit routines. The Debye model in the general form can be written as follows (Eq. S36).

$$I(Q) = \varphi \cdot \frac{\frac{M}{d} \cdot \Delta\rho^2 \cdot P(Q)_{Debye}}{N_A} \quad \text{(S36)}$$

For the added scattering components, these two equations were used in which the R_g and $\Delta\rho$ were fitted, after which they were used in the fuzzy-surface complex coacervate model.

1.5. Internal structure scattering

The model up until now describes the complex coacervates already decently apart from the high Q area. In the complex coacervates, we see an internal structure from the bump in scattering around 0.22 $Å^{-1}$, which can be described by positional charges in polyelectrolyte complexes. To add that to the scattering model, we have taken a scattering contribution as a pseudo-structure factor $S(Q)_{internal}$ on top of the existing scattering contributions, which was written as follows (Eq. S37).

$$S(Q)_{internal} = \frac{|C| \cdot e^{-(Q-Q_{local})^2}}{W \cdot Q_{local} \cdot \sqrt{2\pi} \cdot 2 \cdot (W \cdot Q_{local})^2} \quad \text{(S37)}$$

In which $S(Q)_{internal}$ is the pseudo-structure factor, C fractal scattering, Q_{local} is the position of the structure peak, and W is the relative width of the local Q. These three parameters were fitted in the model.

1.6. Combining all scattering components

Combining all these separate components results in the following scattering equation (Eq. S38).

$$I\,(Q) = \varphi \cdot f_{Coa}\,\left(f_{clu} \cdot S(Q)_{cluster} \cdot I_{Coa}(Q) + (1 - f_{clu}) \cdot I_{Coa}(Q) + \frac{blob\,(Q)}{V_{Coa}}\right) + \varphi\left(f_{Poly} \cdot I_{Poly,free}(Q) \cdot f_{mix} + f_{Col} \cdot I_{Col,free}(Q) \cdot (1 - f_{mix})\right) + f_{Coa} \cdot S(Q)_{internal} + \varphi_{Enzymes} \cdot I_{Enzymes}(Q) + \varphi_{HSA} \cdot I_{HSA}(Q) \quad \text{(S38)}$$

The first part of the scattering equation considers the complex coacervate scattering for the volume fraction and fraction of complex coacervates and includes the cluster structure factor if it is necessary to explain the data. The second part takes the free scattering of the polymer and colistin, considering the volume fraction and the fractions of free fractions in the aqueous environment around the complex coacervates. The last part is additional scattering from the pseudo-structure factor, factored by the fraction of complex coacervates. If there is another component added (enzyme or serum protein), the volume fraction of this component and additional Debye scattering are added on top of the existing scattering.

Since the mass balance and the scattering equation are complete for fitting, we can calculate the molecular weights of the complex coacervates. First, the molecular weights (M_w) of the complex coacervates could be calculated with the following formula (Eq. S39).

$$M_w = P \cdot \,(f_{mix0} \cdot (M_{PMAA} + M_{PEO}) + (1 - f_{mix0}) \cdot M_{Col0}) \quad \text{(S39)}$$

In which M_w is the molecular weight of one complex coacervate structure. In addition, the concentration of colistin taken up by the complex coacervates (c_{ColC3M}) can be calculated (Eq. S40).

$$c_{ColC3M} = (1 - f_{Col}) \cdot c_{Col} \qquad \text{(S40)}$$

The c_{ColC3M} is calculated to get an idea of the efficiency in the encapsulation of colistin for the charge ratios, where a bigger value means a higher encapsulation efficiency. However, the volume fraction of water in these complexes is also of importance. To get more information about the volume fractions of water inside of the complex coacervates, we calculate the water volume fraction (f_w) (Eq. S41).

$$f_w = 1 - \frac{P \cdot V_{tot}}{\frac{4}{3}\pi \cdot \left(R_{in}{}^3 + \frac{R_{in} \cdot \sigma_{in}{}^2}{2}\right)} \qquad \text{(S41)}$$

2. Results and Discussion

2.1. DLS measurements at different charge ratios (f_+) of complex coacervates made from different block lengths of PEO-b-PMAA

The DLS measurements of the sizes of the complex coacervates can be found in Figures S1, S2, and S3. In addition, the autocorrelation function of representative DLS measurements is shown (Figure S4).

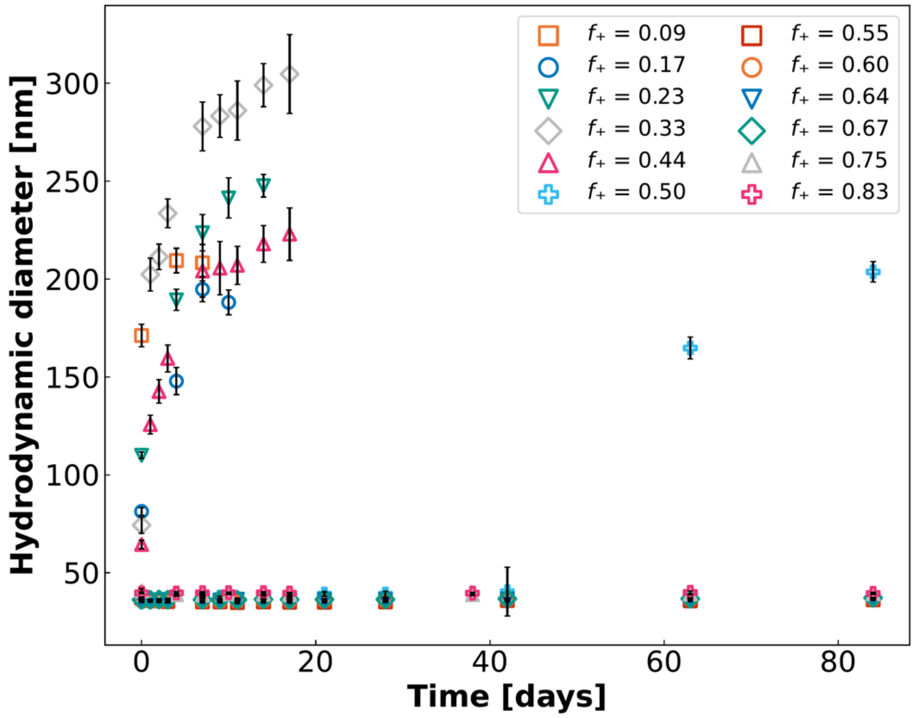

Figure S1. DLS measurements over time of P1-colistin coacervate complexes at several ratios at $0.09 \leq f_+ \leq 0.83$. Aggregation occurs below a f_+ of 0.55. In other cases, coacervate complexes are stable for more than 80 days.

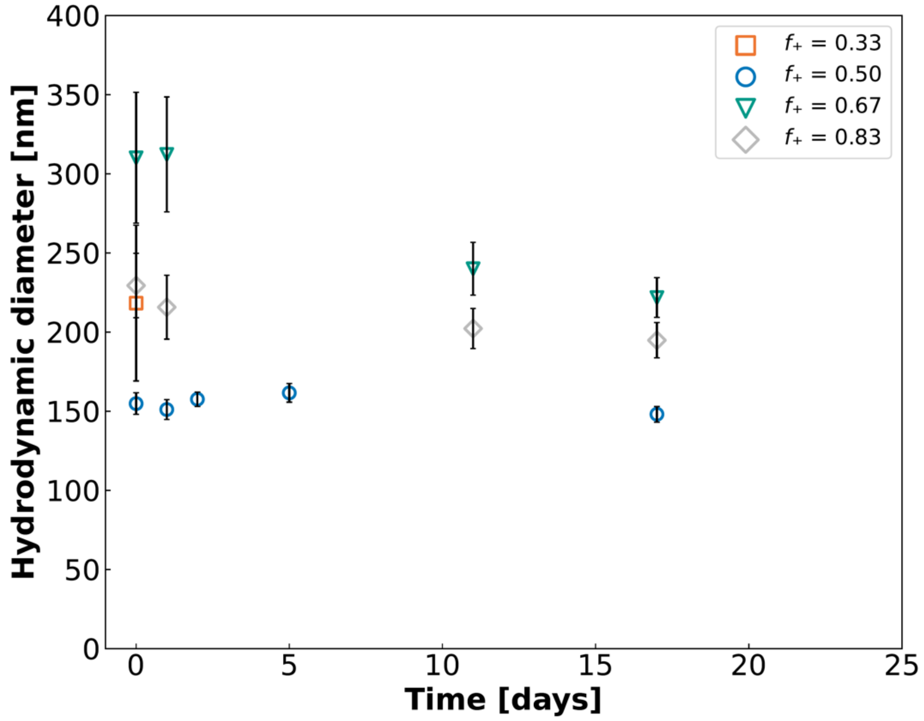

Figure S2. DLS measurements over time of P2-colistin coacervate complexes at several ratios at $0.33 \leq f_+ \leq 0.83$. $f_+ < 0.33$ and $f_+ > 0.83$ were not possible to be measured because of instability and immediate aggregation.

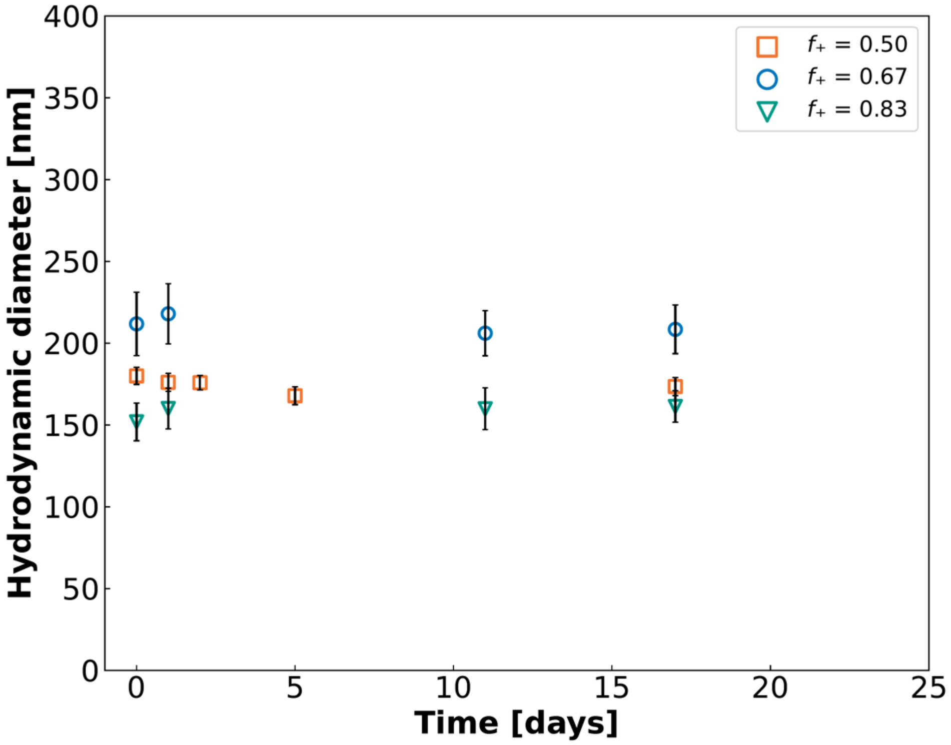

Figure S3. DLS measurements over time of P3-colistin coacervate complexes at several ratios at $0.33 \leq f_+ \leq 0.83$. $f_+ < 0.33$ and $f_+ > 0.83$ were not possible to be measured because of instability and immediate aggregation.

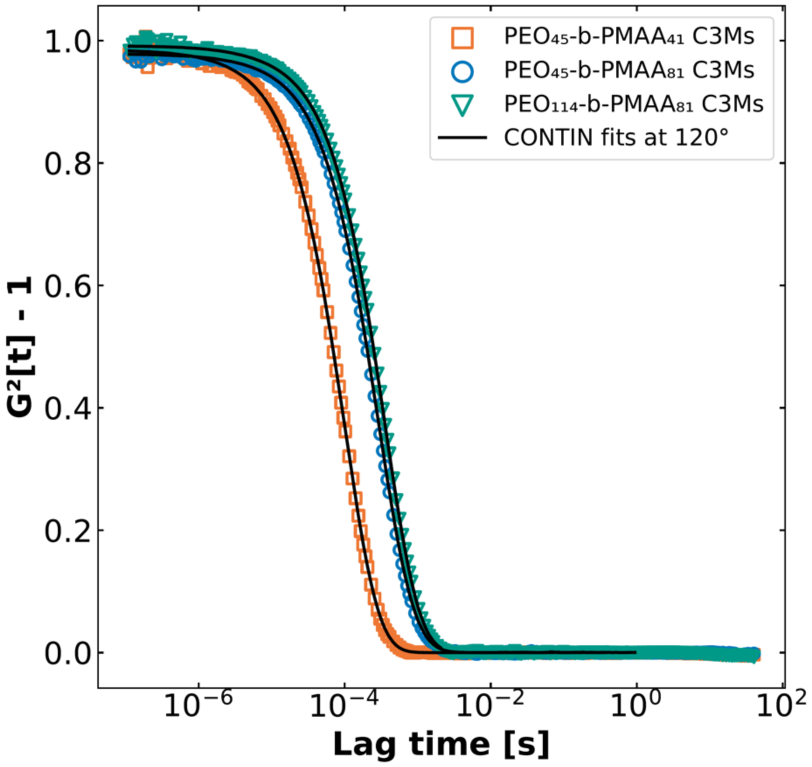

Figure S4. Autocorrelation function including CONTIN fits of a representative sample of DLS measurements performed on several PEO-b-PMAA block length complex coacervates at $f_+ = 0.50$.

2.2. SAXS concentration independence and density profiles of charge ratios

The concentration independence of $0.33 \leq f_+ \leq 0.83$ for P1-colistin-C3Ms (Figure S5) and density profiles of different charge ratios of P1-colistin-C3Ms (Figure S6) are shown.

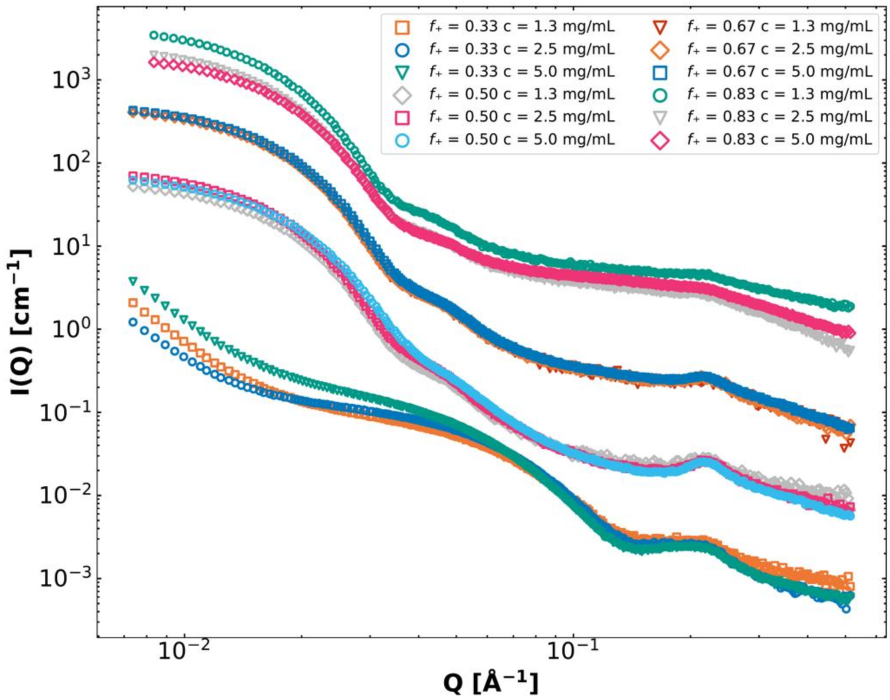

Figure S5. SAXS curves of P1-colistin-C3Ms to show concentration independence between 5.0 mg/mL, 2.5 mg/ml, and 1.3 mg/mL. Four different ratios are plotted, of which the three concentrations are all scaled to 5.0 mg/mL, and then scaled again to improve visuals: $f_+ = 0.33$ is scaled by a factor 1, $f_+ = 0.50$ by a factor 10, $f_+ = 0.67$ by a factor 100 and $f_+ = 0.83$ by a factor 1000. Slight differences can be observed in the scattering pattern but within experimental differences.

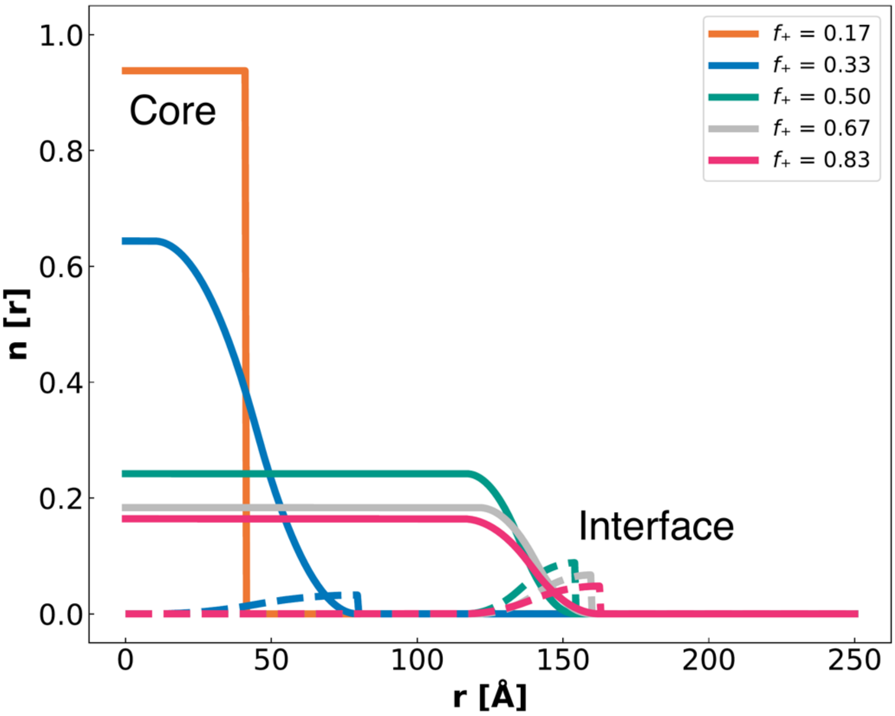

Figure S6. Density profiles obtained from the fuzzy-surface complex coacervate model. Several charge ratios are shown, including their fuzzy-surface parameters where the core transforms into the fuzzy-interface shell. Larger f_+ values cause a decrease in the density of the core, especially in the SCP$^-$-region ($f_+ < 0.40$).

2.3. Varying ionic strength and pH for P1-colistin-C3Ms at $f_+ = 0.50$

The ionic strength is varied in two experimental setups. In the first, the colistin-C3Ms at $f_+ = 0.50$ are prepared in a stopped-flow mixer at a mixing speed of 6.7 mL/s in either regular TRIS

buffer (0.05M, pH = 7.4) or TRIS buffer with added 0.15 M NaCl (to mimic physiological conditions) (Figure S7). Secondly, the annihilation effect of salt was assessed by preparing C3Ms before adding salt (Figure S8). Lastly, the preparation of colistin-C3Ms at different pH values was investigated (Figure S9).

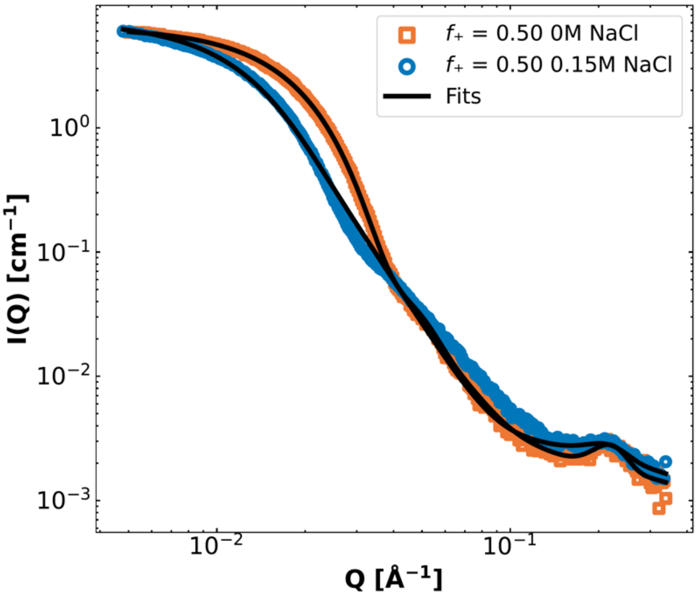

Figure S7. X-ray scattering patterns to demonstrate ionic strength dependency in mixing for P1-colistin coacervate complexes at 5.0 mg/mL, using a stopped-flow device at a mixing rate of 6.7 mL/s. Preparation of P1-colistin C3Ms in the presence of higher ionic strength results in swollen lower-defined particles (increased size, water volume fraction, and polydispersity index). All other properties, like encapsulation, aggregation number, and free colistin fraction are similar (Table S4).

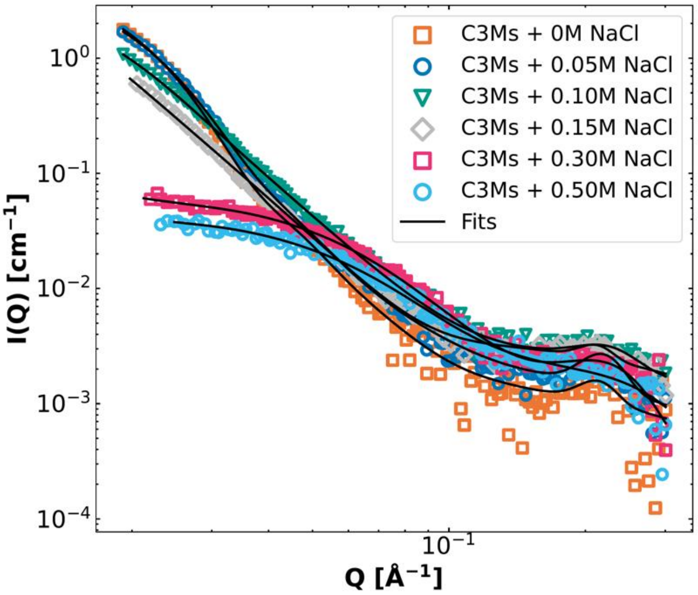

Figure S8. X-ray scattering patterns to indicate the ionic strength dependency in annihilation caused by salt addition for P1-colistin coacervate complexes at 5.0 mg/mL, measured on an in-house SAXS at the University of Oslo (RECX). Until physiological levels of NaCl (until 0.15 NaCl), the C3Ms become less defined (increased *PDI*) and start swelling (R_{tot}, higher f_w). After physiological levels, the complex coacervates are smaller and comprised of a very small number of molecules (Table S5).

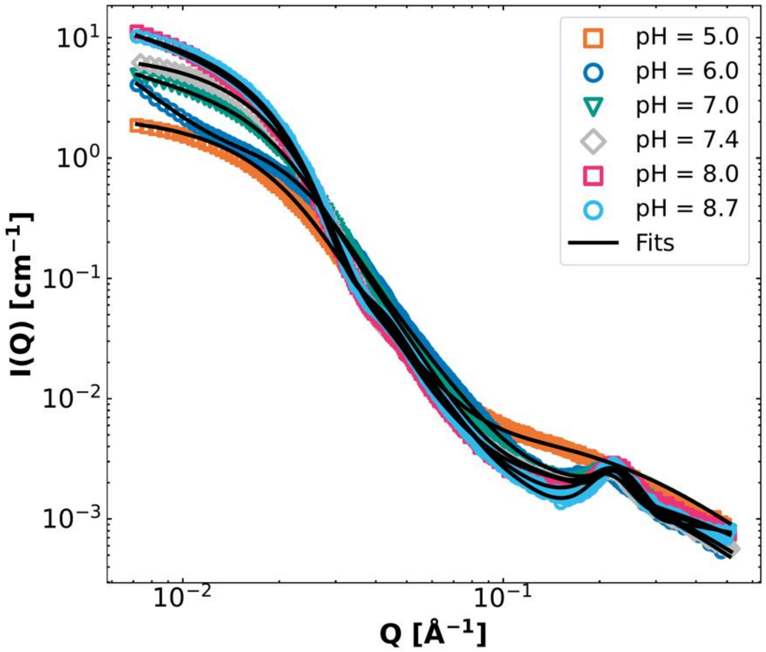

Figure S9. X-ray scattering patterns showcasing the pH dependency of P1-colistin coacervate complexes at 5.0 mg/mL. A decrease in pH leads to a decreased I0, an increase in PDI, and a loss of internal peak structure (Table S6).

2.4. CMC determination

Two ways of CMC determination are shown using surface tension to measure a concentration series (Figure S10) and SAXS (Figure S11).

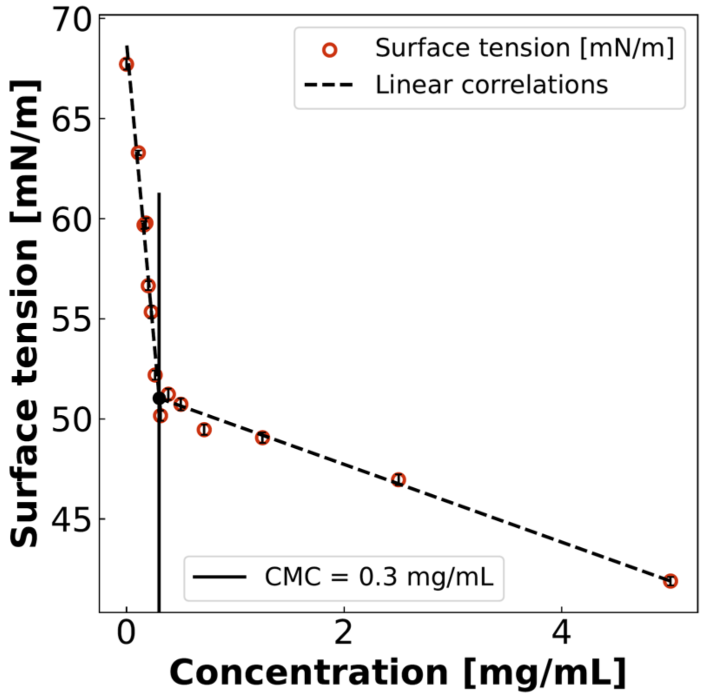

Figure S5. CMC determination of P1-colistin coacervate complexes at $f_+ = 0.50$. With the pendant drop method, the surface tension was determined at several concentrations of P1-colistin coacervate complexes, by which two regions could be determined: high slope and low slope. The intercept is the CMC of the coacervate complexes, which was determined to be 0.3 mg/mL by linear regression of both lines.

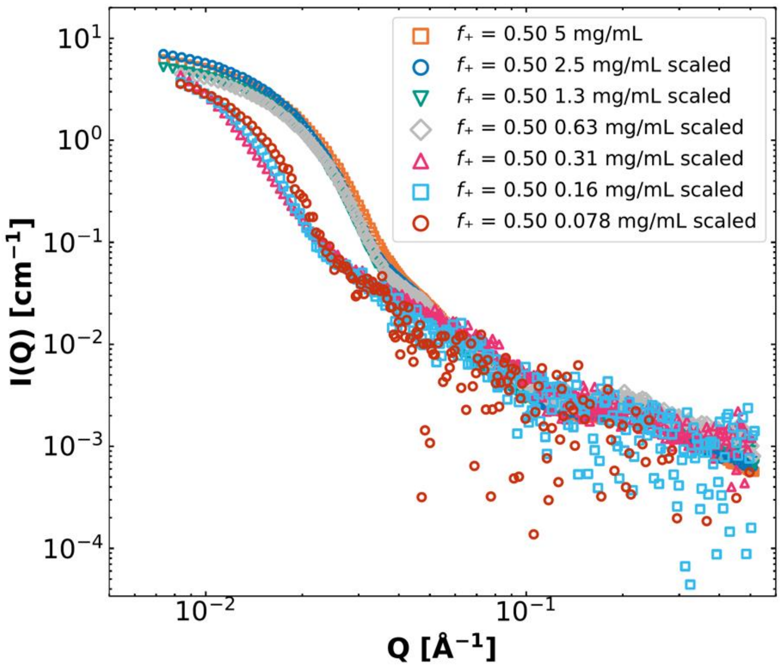

Figure S6. Determination of CMC at f_+ = 0.50 by using SAXS. SAXS patterns are shown at seven different concentrations and scaled to 5.0 mg/mL. The SAXS pattern starts deviating between 0.63 mg/mL and 0.31 mg/mL since the distinguishable features are lost. Therefore, the CMC can be assumed to be around 0.3-0.6 mg/mL.

2.5. Antimicrobial properties

Other bacteria were assessed for antimicrobial properties during the experiment (Figure S12A), while the negative control did not show any inhibition (Figure S12B).

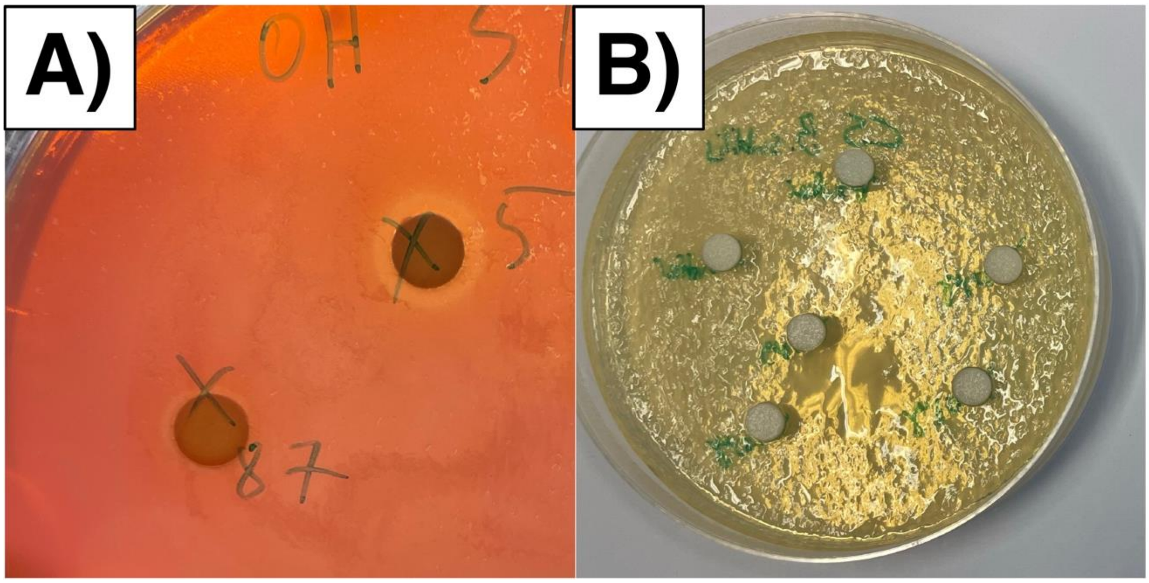

Figure S7. Other bacteria were found to be unsuitable for accurate antimicrobial activity testing. *E. coli* was found to give the clearest results (Fig. 4A). *S. indica* grew over the inhibition zones, making precise measurements difficult (A), and *B. subtilis* served as a control, no inhibition zones could be observed (B).

2.6. Enzymatic breakdown of trypsin

Colistin and colistin-C3Ms were also analyzed for breakdown with trypsin. It was found that trypsin did not break down colistin sufficiently (Figure S13).

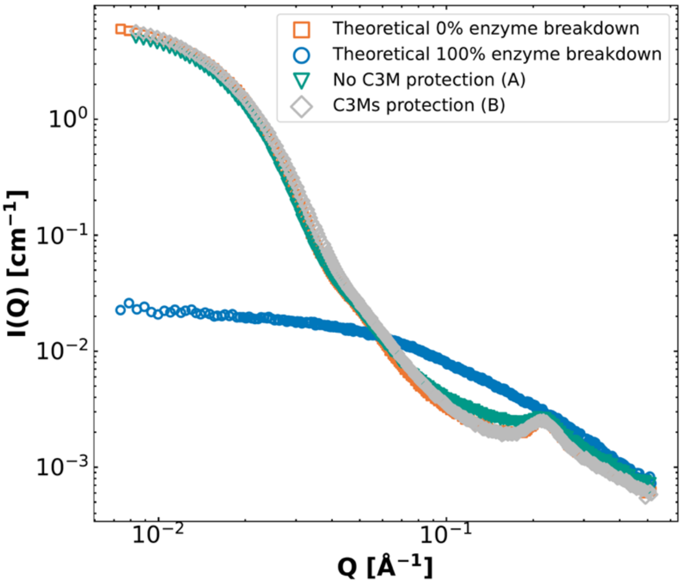

Figure S8. Small-angle X-ray scattering patterns of enzymatic breakdown of colistin (3.4 mg/mL) versus colistin C3Ms (total concentration 5.0 mg/mL) in the presence of trypsin. Theoretical SAXS patterns were calculated based on either the added scattering from C3Ms and enzymes (0% enzyme breakdown, orange squares) or colistin, polymer, and enzyme separately summed up (100% break down, blue circles). The effect of enzymatic degradation after 24 hours at 37°C was measured by either adding Trypsin to colistin followed by complexation with polymer (indicated by green triangles) or the addition of enzyme to C3Ms (indicated by grey diamonds). The SAXS patterns were fitted by using the fuzzy-surface complex coacervate model for complex coacervates with added Debye scattering of the corresponding enzymes. It can be observed that trypsin does not seem to break down colistin.

2.7. Freeze-drying C3Ms

Freeze-dried colistin C3Ms at $f_+ = 0.50$ were compared to non-freeze-dried counterparts using SAXS to check if freeze-drying is a viable method for sterilization (Figure S14).

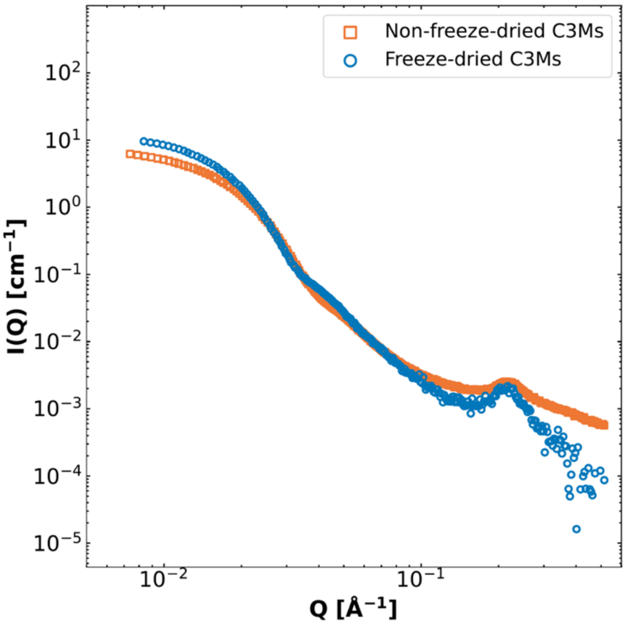

Figure S9. Compared freeze-dried C3Ms (blue circles) versus ordinary C3Ms (orange squares) at $f_+ = 0.50$ at 5.0 mg/mL. Even though the curve changes slightly, the SAXS pattern is similar, especially considering the structure, showing that freeze-drying is a viable method to acquire sterile samples for cell testing.

2.8. Cell morphology changes due to toxicity

The cell morphologies of all analyzed cell types are imaged every 24 hours for all treatments (Figure S15).

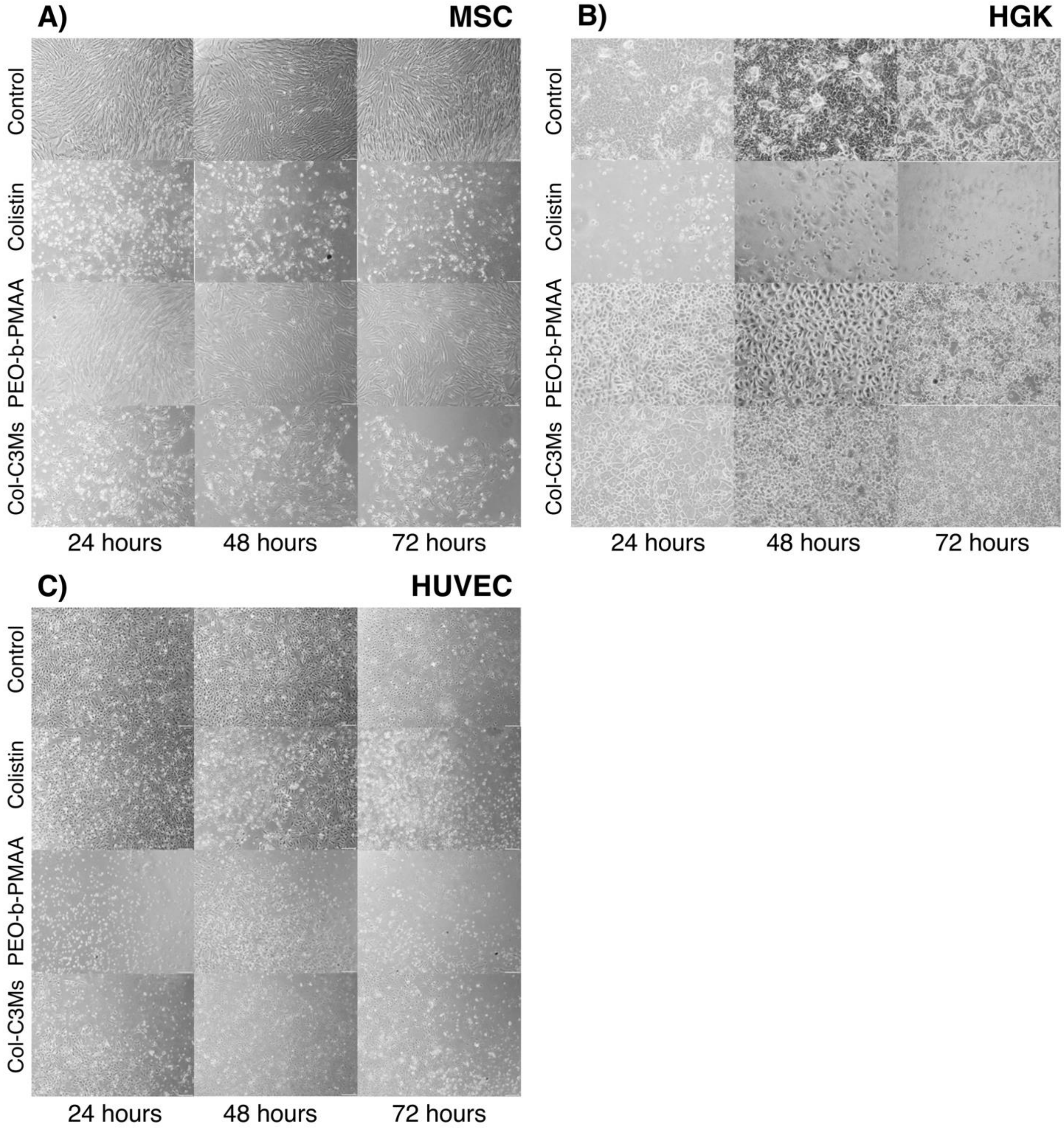

Figure S10. Cell morphologies of MSC (A), HGK (B), and HUVEC (C) after exposure to treatments of colistin, PEO-b-PMAA, and Colistin-C3Ms. Pictures are taken at 1, 2, and 3 days. Lower densities in images can be attributed to cell death, causing cell detachment. The scale bar is 200 μm.

2.9. SAXS fits

All SAXS fits for three concentrations (Table S1-S3), ionic strength dependency (Table S4, S5), pH dependency (Table S6), the enzymatic breakdown experiment (Table S7), and the addition of the protein binding experiment with HSA (Table S8).

Table S1. Ratio experiment fitting values for P1-colistin coacervate complexes at 5.0 mg/mL.

**Model parameters / f_+**	**0.01**	**0.09**	**0.17**	**0.23**	**0.33**	**0.44**	**0.50**	**0.55**	**0.60**	**0.64**	**0.67**	**0.75**	**0.83**	**0.91**	**0.98**
M_{Col} (Da)	1405.65	1405.65	1405.65	1405.65	1405.65	1405.65	1405.65	1405.65	1405.65	1405.65	1405.65	1405.65	1405.65	1405.65	1405.65
$\Delta\rho_{Col}$ (cm^{-2})	$1.24 \cdot 10^{11}$	$1.24 \cdot 10^{11}$	$1.24 \cdot 10^{11}$	$1.24 \cdot 10^{11}$	$1.24 \cdot 10^{11}$	$1.24 \cdot 10^{11}$	$1.24 \cdot 10^{11}$	$1.24 \cdot 10^{11}$	$1.24 \cdot 10^{11}$	$1.24 \cdot 10^{11}$	$1.24 \cdot 10^{11}$	$1.24 \cdot 10^{11}$	$1.24 \cdot 10^{11}$	$1.24 \cdot 10^{11}$	$1.24 \cdot 10^{11}$
$\Delta\rho_{PMAA}$ (cm^{-2})	$1.09 \cdot 10^{11}$	$1.09 \cdot 10^{11}$	$1.09 \cdot 10^{11}$	$1.09 \cdot 10^{11}$	$1.09 \cdot 10^{11}$	$1.09 \cdot 10^{11}$	$1.09 \cdot 10^{11}$	$1.09 \cdot 10^{11}$	$1.09 \cdot 10^{11}$	$1.09 \cdot 10^{11}$	$1.09 \cdot 10^{11}$	$1.09 \cdot 10^{11}$	$1.09 \cdot 10^{11}$	$1.09 \cdot 10^{11}$	$1.09 \cdot 10^{11}$
$\Delta\rho_{PEO}$ (cm^{-2})	$1.11 \cdot 10^{11}$	$1.11 \cdot 10^{11}$	$1.11 \cdot 10^{11}$	$1.11 \cdot 10^{11}$	$1.11 \cdot 10^{11}$	$1.11 \cdot 10^{11}$	$1.11 \cdot 10^{11}$	$1.11 \cdot 10^{11}$	$1.11 \cdot 10^{11}$	$1.11 \cdot 10^{11}$	$1.11 \cdot 10^{11}$	$1.11 \cdot 10^{11}$	$1.11 \cdot 10^{11}$	$1.11 \cdot 10^{11}$	$1.11 \cdot 10^{11}$
$\Delta\rho_{Core}$ (cm^{-2})	$1.24 \cdot 10^{11}$	$1.24 \cdot 10^{11}$	$1.24 \cdot 10^{11}$	$1.24 \cdot 10^{11}$	$1.24 \cdot 10^{11}$	$1.24 \cdot 10^{11}$	$1.24 \cdot 10^{11}$	$1.24 \cdot 10^{11}$	$1.24 \cdot 10^{11}$	$1.24 \cdot 10^{11}$	$1.24 \cdot 10^{11}$	$1.24 \cdot 10^{11}$	$1.24 \cdot 10^{11}$	$1.24 \cdot 10^{11}$	$1.24 \cdot 10^{11}$
$\Delta\rho_{Solvent}$ (cm^{-2})	$9.43 \cdot 10^{10}$	$9.43 \cdot 10^{10}$	$9.43 \cdot 10^{10}$	$9.43 \cdot 10^{10}$	$9.43 \cdot 10^{10}$	$9.43 \cdot 10^{10}$	$9.43 \cdot 10^{10}$	$9.43 \cdot 10^{10}$	$9.43 \cdot 10^{10}$	$9.43 \cdot 10^{10}$	$9.43 \cdot 10^{10}$	$9.43 \cdot 10^{10}$	$9.43 \cdot 10^{10}$	$9.43 \cdot 10^{10}$	$9.43 \cdot 10^{10}$
f_{mix0} (-)	0.09296	0.09296	0.09296	0.09296	0.09296	0.09296	0.09296	0.09296	0.09296	0.09296	0.09296	0.09296	0.09296	0.09296	0.09296
c_{Poly} (mg/mL)	4.90	4.14	3.53	3.08	2.45	1.88	1.62	1.43	1.21	1.05	0.97	0.69	0.44	0.23	0.05
c_{Col} (mg/mL)	0.10	0.86	1.47	1.92	2.55	3.12	3.38	3.57	3.79	3.95	4.03	4.31	4.56	4.77	4.95
P (-)	$2.8 \cdot 10^{2}$	$1.6 \cdot 10^{2}$	$1.5 \cdot 10^{2}$	$1.5 \cdot 10^{2}$	$1.7 \cdot 10^{2}$	$1.3 \cdot 10^{3}$	$1.4 \cdot 10^{3}$	$9.5 \cdot 10^{2}$	$1.2 \cdot 10^{3}$	$1.1 \cdot 10^{3}$	$1.2 \cdot 10^{3}$	$1.1 \cdot 10^{3}$	$1.0 \cdot 10^{3}$	$1.1 \cdot 10^{3}$	$1.4 \cdot 10^{3}$
σ_{in} (nm)	0.0	0.0	0.0	0.0	3.4	1.4	1.8	1.9	1.8	1.9	1,9	2.2	2.3	2.9	3.8
σ_{out} (nm)	0.0	0.0	0.0	0.0	0.0	0.0	0.0	0.0	0.0	0.0	0.0	0.0	0.0	0.0	0.0
R_{in} (nm)	2.1	3.8	4.1	4.2	4.5	11.7	13.5	13.9	13.9	13.9	14.0	13.7	13.9	14.2	15.3
R_{tot} (nm)	2.1	3.8	4.1	4.2	8.0	13.1	15.4	15.7	15.7	15.8	16.0	15.9	16.3	17.1	19.1
f_{clu} (-)	0.03	0.04	0.55	0.35	0.99	0.29	1.0	1.0	1.0	1.0	1.0	1.0	1.0	1.0	1.0
f_{dist} (-)	7.1	3.1	2.9	2.0	1.1	1.0	1.0	1.0	1.0	1.0	1.0	1.0	1.0	1.0	1.0
f_{coa} (-)	0.03	0.25	0.41	0.53	0.71	0.81	0.84	0.85	0.67	0.61	0.54	0.38	0.25	0.13	0.02
$R_{g,Col}$ (nm)	0.643	0.643	0.643	0.643	0.643	0.643	0.643	0.643	0.643	0.643	0.643	0.643	0.643	0.643	0.643
$R_{g,Poly}$ (nm)	2.433	2.433	2.433	2.433	2.433	2.433	2.433	2.433	2.433	2.433	2.433	2.433	2.433	2.433	2.433
C (-)	$8.1 \cdot 10^{-3}$	$6.4 \cdot 10^{-4}$	$1.8 \cdot 10^{-4}$	$1.7 \cdot 10^{-4}$	$1.1 \cdot 10^{-4}$	$7.6 \cdot 10^{-5}$	$5.6 \cdot 10^{-5}$	$4.9 \cdot 10^{-5}$	$6.0 \cdot 10^{-5}$	$6.0 \cdot 10^{-5}$	$6.0 \cdot 10^{-5}$	$8.0 \cdot 10^{-5}$	$6.3 \cdot 10^{-5}$	$1.9 \cdot 10^{-3}$	$4.8 \cdot 10^{-4}$
f_{blob} (-)	11	1.7	0.94	0.97	0.97	0.43	0.38	0.34	0.24	0.21	0.21	0.28	0.43	0.01	0.99
ξ (-)	2.1	2.1	2.1	1.8	3.3	3.2	3.8	4.1	3.9	3.8	3.7	5.3	3.8	3.8	3.8
W (-)	10	0.55	0.19	0.17	0.16	0.11	0.09	0.10	0.10	0.10	0.10	0.12	0.10	2.5	0.37
Q_{local} (Å^{-1})	0.11	0.13	0.20	0.20	0.21	0.22	0.22	0.22	0.22	0.22	0.22	0.22	0.22	0.06	0.13
M_{Col0} (Da)	1165.5	1165.5	1165.5	1165.5	1165.5	1165.5	1165.5	1165.5	1165.5	1165.5	1165.5	1165.5	1165.5	1165.5	1165.5
f_{Col} (-)	0.01	0.02	0.07	0.07	0.06	0.12	0.16	0.20	0.40	0.48	0.54	0.71	0.82	0.91	0.98
f_{Poly} (-)	0.99	0.90	0.81	0.72	0.53	0.30	0.16	0.04	0.10	0.06	0.09	0.11	0.08	0.09	0.18
f_{mix} (-)	1.00	0.98	0.86	0.79	0.65	0.24	0.09	0.02	0.02	0.01	0.01	0.01	0.00	0.00	0.00
M_w (Da)	$4.7 \cdot 10^{5}$	$2.6 \cdot 10^{5}$	$2.5 \cdot 10^{5}$	$2.6 \cdot 10^{5}$	$2.8 \cdot 10^{5}$	$2.2 \cdot 10^{6}$	$2.3 \cdot 10^{6}$	$1.6 \cdot 10^{6}$	$2.0 \cdot 10^{6}$	$1.9 \cdot 10^{6}$	$2.0 \cdot 10^{6}$	$1.9 \cdot 10^{6}$	$1.7 \cdot 10^{6}$	$1.8 \cdot 10^{6}$	$2.4 \cdot 10^{6}$
c_{ColC3M} (mg/mL)	0.10	0.84	1.37	1.79	2.40	2.75	2.84	2.86	2.27	2.07	1.84	1.27	0.84	0.43	0.08
f_w (-)	0.0	0.0	0.0	0.0	0.35	0.65	0.76	0.84	0.81	0.81	0.82	0.81	0.84	0.83	0.76
PDI (%)	29	29	27	24	54	19	18	17	17	17	17	16	16	17	18

Table S2. Ratio experiment fitting values for P1-colistin coacervate complexes at 2.5 mg/mL[a].

**Model parameters / f_+**	**0.33**	**0.44**	**0.50**	**0.55**	**0.60**	**0.64**	**0.67**	**0.75**	**0.83**	**0.91**
M_{Col} (Da)	1405.65	1405.65	1405.65	1405.65	1405.65	1405.65	1405.65	1405.65	1405.65	1405.65
$\Delta\rho_{Col}$ (cm^{-2})	$1.24\cdot10^{11}$	$1.24\cdot10^{11}$	$1.24\cdot10^{11}$	$1.24\cdot10^{11}$	$1.24\cdot10^{11}$	$1.24\cdot10^{11}$	$1.24\cdot10^{11}$	$1.24\cdot10^{11}$	$1.24\cdot10^{11}$	$1.24\cdot10^{11}$
$\Delta\rho_{PMAA}$ (cm^{-2})	$1.09\cdot10^{11}$	$1.09\cdot10^{11}$	$1.09\cdot10^{11}$	$1.09\cdot10^{11}$	$1.09\cdot10^{11}$	$1.09\cdot10^{11}$	$1.09\cdot10^{11}$	$1.09\cdot10^{11}$	$1.09\cdot10^{11}$	$1.09\cdot10^{11}$
$\Delta\rho_{PEO}$ (cm^{-2})	$1.11\cdot10^{11}$	$1.11\cdot10^{11}$	$1.11\cdot10^{11}$	$1.11\cdot10^{11}$	$1.11\cdot10^{11}$	$1.11\cdot10^{11}$	$1.11\cdot10^{11}$	$1.11\cdot10^{11}$	$1.11\cdot10^{11}$	$1.11\cdot10^{11}$
$\Delta\rho_{Core}$ (cm^{-2})	$1.24\cdot10^{11}$	$1.24\cdot10^{11}$	$1.24\cdot10^{11}$	$1.24\cdot10^{11}$	$1.24\cdot10^{11}$	$1.24\cdot10^{11}$	$1.24\cdot10^{11}$	$1.24\cdot10^{11}$	$1.24\cdot10^{11}$	$1.24\cdot10^{11}$
$\Delta\rho_{Solvent}$ (cm^{-2})	$9.43\cdot10^{10}$	$9.43\cdot10^{10}$	$9.43\cdot10^{10}$	$9.43\cdot10^{10}$	$9.43\cdot10^{10}$	$9.43\cdot10^{10}$	$9.43\cdot10^{10}$	$9.43\cdot10^{10}$	$9.43\cdot10^{10}$	$9.43\cdot10^{10}$
f_{mix0} (-)	0.09296	0.09296	0.09296	0.09296	0.09296	0.09296	0.09296	0.09296	0.09296	0.09296
c_{Poly} (mg/mL)	1.23	0.94	0.81	0.72	0.61	0.53	0.48	0.35	0.22	0.11
c_{Col} (mg/mL)	1.27	1.56	1.69	1.78	1.89	1.97	2.02	2.15	2.28	2.39
P (-)	87	$2.6\cdot10^{3}$	$1.6\cdot10^{3}$	$1.1\cdot10^{3}$	$1.0\cdot10^{3}$	$1.1\cdot10^{3}$	$1.1\cdot10^{3}$	$1.4\cdot10^{3}$	$1.3\cdot10^{3}$	$1.4\cdot10^{3}$
σ_{in} (nm)	2.7	0.8	1.6	1.7	2.1	1.9	2.2	1.8	2.3	2.9
σ_{out} (nm)	0.0	0.0	0.0	0.0	0.0	0.0	0.0	0.0	0.0	0.0
R_{in} (nm)	3.7	14.2	14.4	13.8	14.0	14.1	14.1	14.7	14.6	15.2
R_{tot} (nm)	6.4	15.0	16.0	15.5	16.1	16.0	16.4	16.5	16.8	18.0
f_{clu} (-)	0.99	0.01	1.0	1.0	1.0	1.0	1.0	1.0	1.0	1.0
f_{dist} (-)	1.67	0.35	1.0	1.0	1.0	1.0	1.0	1.0	1.0	1.0
f_{coa} (-)	0.69	0.86	0.84	0.83	0.72	0.58	0.55	0.35	0.25	0.13
$R_{g,Col}$ (nm)	0.643	0.643	0.643	0.643	0.643	0.643	0.643	0.643	0.643	0.643
$R_{g,Poly}$ (nm)	2.433	2.433	2.433	2.433	2.433	2.433	2.433	2.433	2.433	2.433
C (-)	$7.8\cdot10^{-5}$	$3.3\cdot10^{-5}$	$3.0\cdot10^{-5}$	$2.6\cdot10^{-5}$	$2.9\cdot10^{-5}$	$2.4\cdot10^{-5}$	$3.8\cdot10^{-5}$	$1.7\cdot10^{-5}$	$3.1\cdot10^{-5}$	$4.5\cdot10^{-5}$
f_{blob} (-)	0.79	0.57	0.36	0.23	0.27	0.22	0.29	0.17	0.00	0.64
ξ (-)	3.1	2.2	3.3	4.0	5.4	2.9	6.9	3.8	3.8	3.8
W (-)	0.19	0.10	0.10	0.09	0.11	0.09	0.11	0.10	0.10	0.10
Q_{local} (Å^{-1})	0.20	0.22	0.22	0.22	0.22	0.22	0.22	0.22	0.22	0.22
M_{Col0} (Da)	1165.5	1165.5	1165.5	1165.5	1165.5	1165.5	1165.5	1165.5	1165.5	1165.5
f_{Col} (-)	0.08	0.07	0.16	0.22	0.36	0.50	0.54	0.73	0.81	0.91
f_{Poly} (-)	0.54	0.26	0.16	0.06	0.04	0.10	0.07	0.18	0.08	0.08
f_{mix} (-)	0.58	0.31	0.09	0.02	0.01	0.01	0.01	0.01	0.00	0.00
M_w (Da)	$1.5\cdot10^{5}$	$3.2\cdot10^{6}$	$2.7\cdot10^{6}$	$1.8\cdot10^{6}$	$1.7\cdot10^{6}$	$1.8\cdot10^{6}$	$1.8\cdot10^{6}$	$2.3\cdot10^{6}$	$2.2\cdot10^{6}$	$2.4\cdot10^{6}$
c_{ColC3M} (mg/mL)	1.17	1.45	1.42	1.40	1.21	0.98	0.93	0.59	0.32	0.22
f_w (-)	0.41	0.60	0.76	0.82	0.84	0.83	0.83	0.81	0.82	0.82
PDI (%)	56	18	17	17	17	16	17	18	17	16

[a]It was not possible to fit SAXS curves at this concentration at $f_+ < 0.33$ and $f_+ > 0.91$.

Table S3. Ratio experiment fitting values for P1-colistin coacervate complexes at 1.3 mg/mL[a].

**Model parameters / f_+**	**0.33**	**0.44**	**0.50**	**0.55**	**0.60**	**0.64**	**0.67**	**0.75**	**0.83**	**0.91**
M_{Col} (Da)	1405.65	1405.65	1405.65	1405.65	1405.65	1405.65	1405.65	1405.65	1405.65	1405.65
$\Delta\rho_{Col}$ (cm^{-2})	$1.24 \cdot 10^{11}$	$1.24 \cdot 10^{11}$	$1.24 \cdot 10^{11}$	$1.24 \cdot 10^{11}$	$1.24 \cdot 10^{11}$	$1.24 \cdot 10^{11}$	$1.24 \cdot 10^{11}$	$1.24 \cdot 10^{11}$	$1.24 \cdot 10^{11}$	$1.24 \cdot 10^{11}$
$\Delta\rho_{PMAA}$ (cm^{-2})	$1.09 \cdot 10^{11}$	$1.09 \cdot 10^{11}$	$1.09 \cdot 10^{11}$	$1.09 \cdot 10^{11}$	$1.09 \cdot 10^{11}$	$1.09 \cdot 10^{11}$	$1.09 \cdot 10^{11}$	$1.09 \cdot 10^{11}$	$1.09 \cdot 10^{11}$	$1.09 \cdot 10^{11}$
$\Delta\rho_{PEO}$ (cm^{-2})	$1.11 \cdot 10^{11}$	$1.11 \cdot 10^{11}$	$1.11 \cdot 10^{11}$	$1.11 \cdot 10^{11}$	$1.11 \cdot 10^{11}$	$1.11 \cdot 10^{11}$	$1.11 \cdot 10^{11}$	$1.11 \cdot 10^{11}$	$1.11 \cdot 10^{11}$	$1.11 \cdot 10^{11}$
$\Delta\rho_{Core}$ (cm^{-2})	$1.24 \cdot 10^{11}$	$1.24 \cdot 10^{11}$	$1.24 \cdot 10^{11}$	$1.24 \cdot 10^{11}$	$1.24 \cdot 10^{11}$	$1.24 \cdot 10^{11}$	$1.24 \cdot 10^{11}$	$1.24 \cdot 10^{11}$	$1.24 \cdot 10^{11}$	$1.24 \cdot 10^{11}$
$\Delta\rho_{Solvent}$ (cm^{-2})	$9.43 \cdot 10^{10}$	$9.43 \cdot 10^{10}$	$9.43 \cdot 10^{10}$	$9.43 \cdot 10^{10}$	$9.43 \cdot 10^{10}$	$9.43 \cdot 10^{10}$	$9.43 \cdot 10^{10}$	$9.43 \cdot 10^{10}$	$9.43 \cdot 10^{10}$	$9.43 \cdot 10^{10}$
f_{mix0} (-)	0.09296	0.09296	0.09296	0.09296	0.09296	0.09296	0.09296	0.09296	0.09296	0.09296
c_{Poly} (mg/mL)	0.61	0.47	0.41	0.36	0.30	0.26	0.24	0.17	0.11	0.06
c_{Col} (mg/mL)	0.64	0.78	0.84	0.89	0.95	0.99	1.01	1.08	1.14	1.19
P (-)	79	$1.9 \cdot 10^{3}$	$1.2 \cdot 10^{3}$	$9.3 \cdot 10^{2}$	$1.3 \cdot 10^{3}$	$1.3 \cdot 10^{3}$	$1.1 \cdot 10^{3}$	$1.3 \cdot 10^{3}$	$2.2 \cdot 10^{3}$	$1.5 \cdot 10^{3}$
σ_{in} (nm)	3.0	1.5	1.7	1.7	1.7	1.9	1.9	2.3	1.8	0.0
σ_{out} (nm)	0.0	0.0	0.0	0.0	0.0	0.0	0.0	0.0	0.0	0.0
R_{in} (nm)	3.4	14.0	14.0	14.0	14.0	15.0	14.3	14.4	14.7	15.6
R_{tot} (nm)	6.4	15.5	15.8	15.8	15.7	16.9	16.2	16.7	16.4	15.6
f_{clu} (-)	0.99	1.0	1.0	1.0	1.0	1.0	1.0	1.0	1.0	1.0
f_{dist} (-)	1.38	1.0	1.0	1.0	1.0	1.0	1.0	1.0	1.0	1.0
f_{coa} (-)	0.70	0.82	0.84	0.83	0.69	0.64	0.56	0.37	0.25	0.14
$R_{g,Col}$ (nm)	0.643	0.643	0.643	0.643	0.643	0.643	0.643	0.643	0.643	0.643
$R_{g,Poly}$ (nm)	2.433	2.433	2.433	2.433	2.433	2.433	2.433	2.433	2.433	2.433
C (-)	$4.7 \cdot 10^{-5}$	$1.0 \cdot 10^{-5}$	$1.1 \cdot 10^{-5}$	$9.3 \cdot 10^{-6}$	$1.4 \cdot 10^{-5}$	$1.4 \cdot 10^{-5}$	$1.3 \cdot 10^{-5}$	$1.5 \cdot 10^{-5}$	$3.8 \cdot 10^{-5}$	$4.3 \cdot 10^{-5}$
f_{blob} (-)	0.78	0.65	0.44	0.31	0.31	0.25	0.16	0.02	0.92	0.66
ξ (-)	1.9	2.7	3.1	2.8	2.0	2.8	3.3	3.8	1.8	1.9
W (-)	0.20	0.10	0.10	0.10	0.10	0.10	0.10	0.10	0.14	0.19
Q_{local} (Å^{-1})	0.21	0.22	0.22	0.22	0.22	0.22	0.22	0.22	0.21	0.22
M_{Col0} (Da)	1165.5	1165.5	1165.5	1165.5	1165.5	1165.5	1165.5	1165.5	1165.5	1165.5
f_{Col} (-)	0.08	0.11	0.16	0.22	0.38	0.45	0.53	0.71	0.81	0.90
f_{Poly} (-)	0.54	0.29	0.16	0.06	0.07	0.01	0.06	0.121	0.07	0.01
f_{mix} (-)	0.59	0.25	0.09	0.02	0.01	0.00	0.01	0.01	0.00	0.00
M_w (Da)	$1.3 \cdot 10^{5}$	$3.2 \cdot 10^{6}$	$2.0 \cdot 10^{6}$	$1.6 \cdot 10^{6}$	$2.1 \cdot 10^{6}$	$2.2 \cdot 10^{6}$	$1.8 \cdot 10^{6}$	$2.1 \cdot 10^{6}$	$3.6 \cdot 10^{6}$	$2.5 \cdot 10^{6}$
c_{ColC3M} (mg/mL)	0.59	0.69	0.71	0.70	0.59	0.54	0.47	0.32	0.21	0.12
f_w (-)	0.37	0.70	0.81	0.85	0.80	0.83	0.84	0.81	0.70	0.82
PDI (%)	68	17	17	17	17	17	17	16	17	16

[a]It was not possible to fit SAXS curves at this concentration at $f_+ < 0.33$ and $f_+ > 0.91$.

Table S4. Model parameters for the ionic strength dependency in mixing for P1-colistin coacervate complexes at 5.0 mg/mL, using a stopped-flow device at a mixing rate of 6.7 mL/s.

**Model parameters**	C3Ms	C3Ms + 0.15M NaCl
M_{Col} (Da)	1405.65	1405.65
$\Delta\rho_{Col}$ (cm^{-2})	$1.24 \cdot 10^{11}$	$1.24 \cdot 10^{11}$
$\Delta\rho_{PMAA}$ (cm^{-2})	$1.09 \cdot 10^{11}$	$1.09 \cdot 10^{11}$
$\Delta\rho_{PEO}$ (cm^{-2})	$1.11 \cdot 10^{11}$	$1.11 \cdot 10^{11}$
$\Delta\rho_{Core}$ (cm^{-2})	$1.24 \cdot 10^{11}$	$1.24 \cdot 10^{11}$
$\Delta\rho_{Solvent}$ (cm^{-2})	$9.43 \cdot 10^{10}$	$9.46 \cdot 10^{10}$
f_{mix0} (-)	0.09296	0.09296
c_{Poly} (mg/mL)	1.62	1.55
c_{Col} (mg/mL)	3.38	3.22
P (-)	$1.2 \cdot 10^{3}$	$1.3 \cdot 10^{3}$
σ_{in} (nm)	0.3	0.0
σ_{out} (nm)	0.0	0.0
R_{in} (nm)	12.4	17.8
R_{tot} (nm)	12.7	17.8
f_{clu} (-)	1.0	1.0
f_{dist} (-)	1.0	1.0
f_{coa} (-)	0.86	0.86
$R_{g,Col}$ (nm)	0.643	0.643
$R_{g,Poly}$ (nm)	2.433	2.433
C (-)	$7.9 \cdot 10^{-5}$	$6.1 \cdot 10^{-5}$
f_{blob} (-)	0.30	0.40
ξ (-)	1.8	2.0
W (-)	0.12	0.16
Q_{local} (Å^{-1})	0.22	0.21
M_{Col0} (Da)	1165.5	1165.5
f_{Col} (-)	0.14	0.14
f_{Poly} (-)	0.14	0.14
f_{mix} (-)	0.093	0.093
M_w (Da)	$2.0 \cdot 10^{6}$	$2.3 \cdot 10^{6}$
c_{ColC3M} (mg/mL)	2.9	2.9
f_w (-)	0.71	0.88
PDI (%)	17	27

Table S5. Model parameters for the ionic strength dependency after mixing (salt annihilation) for P1-colistin coacervate complexes at 5.0 mg/mL.

**Model parameters**	C3Ms	C3Ms + 0.05M NaCl	C3Ms + 0.10M NaCl	C3Ms + 0.15M NaCl	C3Ms + 0.30M NaCl	C3Ms + 0.50M NaCl
M_{Col} (Da)	1405.65	1405.65	1405.65	1405.65	1405.65	1405.65
$\Delta\rho_{Col}$ (cm^{-2})	$1.24\cdot10^{11}$	$1.24\cdot10^{11}$	$1.24\cdot10^{11}$	$1.24\cdot10^{11}$	$1.24\cdot10^{11}$	$1.24\cdot10^{11}$
$\Delta\rho_{PMAA}$ (cm^{-2})	$1.09\cdot10^{11}$	$1.09\cdot10^{11}$	$1.09\cdot10^{11}$	$1.09\cdot10^{11}$	$1.09\cdot10^{11}$	$1.09\cdot10^{11}$
$\Delta\rho_{PEO}$ (cm^{-2})	$1.11\cdot10^{11}$	$1.11\cdot10^{11}$	$1.11\cdot10^{11}$	$1.11\cdot10^{11}$	$1.11\cdot10^{11}$	$1.11\cdot10^{11}$
$\Delta\rho_{Core}$ (cm^{-2})	$1.24\cdot10^{11}$	$1.24\cdot10^{11}$	$1.24\cdot10^{11}$	$1.24\cdot10^{11}$	$1.24\cdot10^{11}$	$1.24\cdot10^{11}$
$\Delta\rho_{Solvent}$ (cm^{-2})	$9.43\cdot10^{10}$	$9.43\cdot10^{10}$	$9.45\cdot10^{10}$	$9.46\cdot10^{10}$	$9.51\cdot10^{10}$	$9.54\cdot10^{10}$
f_{mix0} (-)	0.09296	0.09296	0.09296	0.09296	0.09296	0.09296
c_{Poly} (mg/mL)	1.62	1.62	1.62	1.62	1.62	1.62
c_{Col} (mg/mL)	3.38	3.38	3.38	3.38	3.38	3.38
P (-)	$1.3\cdot10^{3}$	$1.2\cdot10^{3}$	$0.9\cdot10^{3}$	$1.4\cdot10^{3}$	$1\cdot10^{1}$	9
σ_{in} (nm)	1.8	1.3	1.2	3.2	0.0	0.0
σ_{out} (nm)	0.0	0.0	0.0	0.0	0.0	0.0
R_{in} (nm)	13.1	13.0	14.3	19.0	4.0	3.9
R_{tot} (nm)	14.9	14.3	15.6	22.2	4.0	3.9
f_{clu} (-)	1.0	1.0	1.0	1.0	0.25	0.31
f_{dist} (-)	1.0	1.0	1.0	1.0	2.4	2.6
f_{coa} (-)	0.85	0.85	0.85	0.85	0.86	0.85
$R_{g,Col}$ (nm)	0.643	0.643	0.643	0.643	0.643	0.643
$R_{g,Poly}$ (nm)	2.433	2.433	2.433	2.433	2.433	2.433
C (-)	$3.1\cdot10^{-5}$	$8.9\cdot10^{-5}$	$9.1\cdot10^{-5}$	$3.8\cdot10^{-5}$	$2.0\cdot10^{-4}$	$3.1\cdot10^{-4}$
f_{blob} (-)	0.18	0.25	0.53	0.58	0.0	0.0
ξ (-)	3.3	3.3	3.3	3.3	3.3	3.3
W (-)	0.09	0.10	0.15	0.08	0.20	0.47
Q_{local} ($Å^{-1}$)	0.21	0.22	0.22	0.22	0.22	0.23
M_{Col0} (Da)	1165.5	1165.5	1165.5	1165.5	1165.5	1165.5
f_{Col} (-)	0.15	0.15	0.15	0.15	0.14	0.15
f_{Poly} (-)	0.15	0.15	0.15	0.15	0.14	0.15
f_{mix} (-)	0.093	0.093	0.093	0.093	0.093	0.093
M_w (Da)	$2.1\cdot10^{6}$	$1.9\cdot10^{6}$	$1.5\cdot10^{6}$	$2.3\cdot10^{6}$	$2.0\cdot10^{5}$	$1.0\cdot10^{5}$
c_{ColC3M} (mg/mL)	2.9	2.9	2.9	2.9	2.9	2.9
f_w (-)	0.71	0.73	0.85	0.90	0.90	0.92
PDI (%)	19	20	28	30	30	30

Table S6. Model parameters for pH dependency experiment (Fig. 5) for P1-colistin coacervate complexes at 5.0 mg/mL.

**Model parameters**	pH = 5.0	pH = 6.0	pH = 7.0	pH = 7.4	pH = 8.0	pH = 8.7
M_{Col} (Da)	1405.65	1405.65	1405.65	1405.65	1405.65	1405.65
$\Delta\rho_{Col}$ (cm^{-2})	$1.24\cdot10^{11}$	$1.24\cdot10^{11}$	$1.24\cdot10^{11}$	$1.24\cdot10^{11}$	$1.24\cdot10^{11}$	$1.24\cdot10^{11}$
$\Delta\rho_{PMAA}$ (cm^{-2})	$1.09\cdot10^{11}$	$1.09\cdot10^{11}$	$1.09\cdot10^{11}$	$1.09\cdot10^{11}$	$1.09\cdot10^{11}$	$1.09\cdot10^{11}$
$\Delta\rho_{PEO}$ (cm^{-2})	$1.11\cdot10^{11}$	$1.11\cdot10^{11}$	$1.11\cdot10^{11}$	$1.11\cdot10^{11}$	$1.11\cdot10^{11}$	$1.11\cdot10^{11}$
$\Delta\rho_{Core}$ (cm^{-2})	$1.24\cdot10^{11}$	$1.24\cdot10^{11}$	$1.24\cdot10^{11}$	$1.24\cdot10^{11}$	$1.24\cdot10^{11}$	$1.24\cdot10^{11}$
$\Delta\rho_{Solvent}$ (cm^{-2})	$9.43\cdot10^{10}$	$9.43\cdot10^{10}$	$9.43\cdot10^{10}$	$9.43\cdot10^{10}$	$9.43\cdot10^{10}$	$9.43\cdot10^{10}$
f_{mix0} (-)	0.09296	0.09296	0.09296	0.09296	0.09296	0.09296
c_{Poly} (mg/mL)	1.62	1.62	1.62	1.62	1.62	1.62
c_{Col} (mg/mL)	3.38	3.38	3.38	3.38	3.38	3.38
P (-)	$0.8\cdot10^{3}$	$0.9\cdot10^{3}$	$1.1\cdot10^{3}$	$1.4\cdot10^{3}$	$1.7\cdot10^{3}$	$1.8\cdot10^{3}$
σ_{in} (nm)	0.0	0.0	0.0	1.8	0.0	0.0
σ_{out} (nm)	0.0	0.0	0.0	0.0	0.0	0.0
R_{in} (nm)	13.5	12.0	13.3	13.5	14.2	14.5
R_{tot} (nm)	13.5	12.0	13.3	15.4	14.2	14.5
f_{clu} (-)	1.0	0.01	0.2	1.0	1.0	1.0
f_{dist} (-)	1.0	0.3	8.4	1.0	1.0	1.0
f_{coa} (-)	0.42	0.40	0.76	0.84	0.85	0.98
$R_{g,Col}$ (nm)	0.643	0.643	0.643	0.643	0.643	0.643
$R_{g,Poly}$ (nm)	2.433	2.433	2.433	2.433	2.433	2.433
C (-)	$3.7\cdot10^{-4}$	$9.7\cdot10^{-5}$	$5.9\cdot10^{-5}$	$5.6\cdot10^{-5}$	$1.3\cdot10^{-4}$	$1.6\cdot10^{-4}$
f_{blob} (-)	0.29	0.02	0.26	0.38	0.17	0.12
ξ (-)	2.1	1.6	2.2	3.8	1.2	0.0
W (-)	0.37	0.14	0.09	0.09	0.14	0.17
Q_{local} ($Å^{-1}$)	0.13	0.21	0.22	0.22	0.22	0.23
M_{Col0} (Da)	1165.5	1165.5	1165.5	1165.5	1165.5	1165.5
f_{Col} (-)	0.58	0.60	0.24	0.16	0.15	0.02
f_{Poly} (-)	0.58	0.60	0.24	0.16	0.15	0.02
f_{mix} (-)	0.093	0.093	0.093	0.093	0.093	0.093
M_w (Da)	$1.5\cdot10^{6}$	$1.5\cdot10^{6}$	$1.8\cdot10^{6}$	$2.3\cdot10^{6}$	$2.9\cdot10^{6}$	$3.1\cdot10^{6}$
c_{ColC3M} (mg/mL)	1.2	1.2	2.2	2.8	2.5	2.8
f_w (-)	0.81	0.72	0.76	0.76	0.69	0.69
PDI (%)	28	26	23	18	17	17

Table S7. Model parameters for the enzymatic breakdown experiment (Fig. 5) for P1-colistin coacervate complexes at 5.0 mg/mL.

**Model parameters**	C3Ms	Proteinase K + C3Ms	Proteinase K + Colistin	Subtilisin + C3Ms	Subtilisin + Colistin
M_{Col} (Da)	1405.65	1405.65	1405.65	1405.65	1405.65
$\Delta\rho_{Col}$ (cm^{-2})	$1.24 \cdot 10^{11}$	$1.24 \cdot 10^{11}$	$1.24 \cdot 10^{11}$	$1.24 \cdot 10^{11}$	$1.24 \cdot 10^{11}$
$\Delta\rho_{PMAA}$ (cm^{-2})	$1.09 \cdot 10^{11}$	$1.09 \cdot 10^{11}$	$1.09 \cdot 10^{11}$	$1.09 \cdot 10^{11}$	$1.09 \cdot 10^{11}$
$\Delta\rho_{PEO}$ (cm^{-2})	$1.11 \cdot 10^{11}$	$1.11 \cdot 10^{11}$	$1.11 \cdot 10^{11}$	$1.11 \cdot 10^{11}$	$1.11 \cdot 10^{11}$
$\Delta\rho_{Core}$ (cm^{-2})	$1.24 \cdot 10^{11}$	$1.24 \cdot 10^{11}$	$1.24 \cdot 10^{11}$	$1.24 \cdot 10^{11}$	$1.24 \cdot 10^{11}$
$\Delta\rho_{Solvent}$ (cm^{-2})	$9.43 \cdot 10^{10}$	$9.43 \cdot 10^{10}$	$9.43 \cdot 10^{10}$	$9.43 \cdot 10^{10}$	$9.43 \cdot 10^{10}$
f_{mix0} (-)	0.09296	0.09296	0.09296	0.09296	0.09296
c_{Poly} (mg/mL)	1.62	1.55	1.55	1.55	1.55
c_{Col} (mg/mL)	3.38	3.22	3.22	3.22	3.22
P (-)	$1.4 \cdot 10^{3}$	$1.3 \cdot 10^{3}$	75	$7.5 \cdot 10^{2}$	25
σ_{in} (nm)	1.8	2.2	0.0	0.0	0.0
σ_{out} (nm)	0.0	0.0	0.0	0.0	0.0
R_{in} (nm)	13.5	13.9	4.3	12.5	4.3
R_{tot} (nm)	15.4	16.1	4.3	12.5	4.3
f_{clu} (-)	1.0	1.0	1.0	1.0	1.0
f_{dist} (-)	1.0	1.0	1.0	1.0	1.0
f_{coa} (-)	0.84	0.91	0.21	0.97	0.72
$R_{g,Col}$ (nm)	0.643	0.643	0.643	0.643	0.643
$R_{g,Poly}$ (nm)	2.433	2.433	2.433	2.433	2.433
C (-)	$5.6 \cdot 10^{-5}$	$5.2 \cdot 10^{-5}$	$7.6 \cdot 10^{-11}$	$9.1 \cdot 10^{-5}$	$7.9 \cdot 10^{-5}$
f_{blob} (-)	0.38	0.17	0.01	0.19	0.21
ξ (-)	3.8	2.2	0.10	1.4	2.0
W (-)	0.093	0.10	0.23	0.14	0.23
Q_{local} ($Å^{-1}$)	0.22	0.22	0.22	0.21	0.21
M_{Col0} (Da)	1165.5	1165.5	1165.5	1165.5	1165.5
f_{Col} (-)	0.16	0.09	0.79	0.03	0.28
f_{Poly} (-)	0.16	0.09	0.79	0.03	0.28
f_{mix} (-)	0.093	0.093	0.093	0.093	0.093
M_w (Da)	$2.3 \cdot 10^{6}$	$2.1 \cdot 10^{6}$	$1.0 \cdot 10^{5}$	$1.1 \cdot 10^{6}$	$4 \cdot 10^{4}$
f_w (-)	0.76	0.76	0.51	0.83	0.63
c_{ColC3M} (mg/mL)	2.8	2.9	0.66	3.1	2.3
$R_{g,Enzymes}$ (nm)	-	2.31	2.31	4.120	4.120
$\Delta\rho_{Enzymes}$ (cm^{-2})	-	$1.23 \cdot 10^{11}$	$1.23 \cdot 10^{11}$	$1.23 \cdot 10^{11}$	$1.23 \cdot 10^{11}$
$c_{Enzymes}$ (mg/mL)	-	0.465	0.465	0.9299	0.9299
PDI (%)	18	21	22	21	23

Table S8. Model parameters for the human serum protein binding experiment (Fig. 6) for P1-colistin coacervate complexes at 5.0 mg/mL.

**Model parameters**	C3Ms	C3Ms + HSA 1:10	C3Ms + HSA 1:20
M_{Col} (Da)	1405.65	1405.65	1405.65
$\Delta\rho_{Col}$ (cm^{-2})	$1.24\cdot10^{11}$	$1.24\cdot10^{11}$	$1.24\cdot10^{11}$
$\Delta\rho_{PMAA}$ (cm^{-2})	$1.09\cdot10^{11}$	$1.09\cdot10^{11}$	$1.09\cdot10^{11}$
$\Delta\rho_{PEO}$ (cm^{-2})	$1.11\cdot10^{11}$	$1.11\cdot10^{11}$	$1.11\cdot10^{11}$
$\Delta\rho_{Core}$ (cm^{-2})	$1.24\cdot10^{11}$	$1.24\cdot10^{11}$	$1.24\cdot10^{11}$
$\Delta\rho_{Solvent}$ (cm^{-2})	$9.43\cdot10^{10}$	$9.43\cdot10^{10}$	$9.43\cdot10^{10}$
f_{mix0} (-)	0.09296	0.09296	0.09296
c_{Poly} (mg/mL)	1.62	1.57	1.60
c_{Col} (mg/mL)	3.38	3.27	3.32
P (-)	$1.4\cdot10^{3}$	$1.5\cdot10^{3}$	$1.4\cdot10^{3}$
σ_{in} (nm)	1.8	0.0	1.3
σ_{out} (nm)	0.0	0.0	0.0
R_{in} (nm)	13.5	13.8	13.5
R_{tot} (nm)	15.4	13.8	14.8
f_{clu} (-)	1.0	1.0	1.0
f_{dist} (-)	1.0	1.0	1.0
f_{coa} (-)	0.84	0.84	0.84
$R_{g,Col}$ (nm)	0.643	0.643	0.643
$R_{g,Poly}$ (nm)	2.433	2.433	2.433
C (-)	$5.6\cdot10^{-5}$	$9.0\cdot10^{-5}$	$7.2\cdot10^{-5}$
f_{blob} (-)	0.38	0.23	0.32
ξ (-)	3.8	2.3	3.1
W (-)	0.093	0.13	0.11
Q_{local} (Å^{-1})	0.22	0.22	0.22
M_{Col0} (Da)	1165.5	1165.5	1165.5
f_{Col} (-)	0.16	0.16	0.16
f_{Poly} (-)	0.16	0.16	0.16
f_{mix} (-)	0.093	0.093	0.093
M_w (Da)	$2.3\cdot10^{6}$	$2.4\cdot10^{6}$	$2.3\cdot10^{6}$
f_w (-)	0.76	0.71	0.71
c_{ColC3M} (mg/mL)	2.8	2.7	2.8
$R_{g,HSA}$ (nm)	-	5.1	5.1
$\Delta\rho_{HSA}$ (cm^{-2})	-	$1.10\cdot10^{11}$	$1.10\cdot10^{11}$
c_{HSA} (mg/mL)	-	0.316	0.165
PDI (%)	18	20	19

3. References

(1) Amann, M.; Diget, J. S.; Lyngsø, J.; Pedersen, J. S.; Narayanan, T.; Lund, R. Kinetic Pathways for Polyelectrolyte Coacervate Micelle Formation Revealed by Time-Resolved Synchrotron SAXS. *Macromolecules* **2019**, *52* (21), 8227–8237. https://doi.org/10.1021/acs.macromol.9b01072.
(2) Berndt, I.; Pedersen, J. S.; Lindner, P.; Richtering, W. Influence of Shell Thickness and Cross-Link Density on the Structure of Temperature-Sensitive Poly-N-Isopropylacrylamide−Poly-N-Isopropylmethacrylamide Core−Shell Microgels Investigated by Small-Angle Neutron Scattering. *Langmuir* **2006**, *22* (1), 459–468. https://doi.org/10.1021/la052463u.
(3) Berndt, I.; Pedersen, J. S.; Richtering, W. Temperature-Sensitive Core–Shell Microgel Particles with Dense Shell. *Angew. Chem.* **2006**, *118* (11), 1769–1773. https://doi.org/10.1002/ange.200503888.
(4) Burchard, W.; Kajiwara, K.; Whiffen, D. H. The Statistics of Stiff Chain Molecules I. The Particle Scattering Factor. *Proc. R. Soc. Lond. Math. Phys. Sci.* **1997**, *316* (1525), 185–199. https://doi.org/10.1098/rspa.1970.0074.
(5) Debye, P. Molecular-Weight Determination by Light Scattering. *J. Phys. Colloid Chem.* **1947**, *51* (1), 18–32. https://doi.org/10.1021/j150451a002.
(6) Zhang, F.; Skoda, M. W. A.; Jacobs, R. M. J.; Martin, R. A.; Martin, C. M.; Schreiber, F. Protein Interactions Studied by SAXS: Effect of Ionic Strength and Protein Concentration for BSA in Aqueous Solutions. *J. Phys. Chem. B* **2007**, *111* (1), 251–259. https://doi.org/10.1021/jp0649955.
(7) Bendedouch, D.; Chen, S. H. Structure and Interparticle Interactions of Bovine Serum Albumin in Solution Studied by Small-Angle Neutron Scattering. *J. Phys. Chem.* **1983**, *87* (9), 1473–1477. https://doi.org/10.1021/j100232a003.
